# A systematic review and critical appraisal of quality indicators to assess optimal palliative care for older people with dementia

**DOI:** 10.1177/0269216319834227

**Published:** 2019-03-11

**Authors:** Sarah Amador, Elizabeth L Sampson, Claire Goodman, Louise Robinson, SEED Research Team

**Affiliations:** 1 Marie Curie Palliative Care Research Department, Division of Psychiatry, University College London, London, UK; 2 Centre for Research in Primary and Community Care, University of Hertfordshire, Hatfield, UK; 3 Institute for Ageing and Institute of Health & Society, Newcastle University, Newcastle upon Tyne, UK

**Keywords:** Systematic review, palliative care, end-of-life care, terminal care, quality indicators, healthcare, dementia

## Abstract

**Background:**

A challenge for commissioners and providers of end-of-life care in dementia is to translate recommendations for good or effective care into quality indicators that inform service development and evaluation.

**Aim:**

To identify and critically evaluate quality indicators for end-of-life care in dementia.

**Results:**

We found 8657 references, after de-duplication. In all, 19 publications describing 10 new and 3 updated sets of indicators were included in this review. Ultimately, 246 individual indicators were identified as being relevant to dementia end-of-life care and mapped against EAPC guidelines.

**Conclusions:**

We systematically derived and assessed a set of quality indicators using a robust framework that provides clear definitions of aspects of palliative care, which are dementia specific, and strengthens the theoretical underpinning of new complex interventions in end-of-life care in dementia.

**What is already known about the topic?**

- Dementia is becoming a leading cause of death, but end-of-life care for people with dementia may be poor with improvements needed in many areas.
- Standard measures of quality and efficacy of care used in other medical conditions may not be appropriate in the assessment of outcomes for end-of-life care in patients with dementia.
- Research for people with dementia at the end of life tends to borrow definitions of what constitutes good care from cancer models, without analysing which elements are transferable and which are not.

**What this paper adds?**

- A summary of quality indicators available to assess optimal palliative care in older people with dementia, derived using a robust framework that provides clear definitions of aspects of palliative care which are dementia specific.
- Identification of major gaps related to aspects of palliative care in dementia for which indicators remain to be developed.

**Implications for practice, theory or policy**

- Results provide a dementia-specific resource and framework for future research and the rigorous development and evaluation of complex intervention in end-of-life care in dementia.

## Background

Dementia has become the leading cause of death among women in England, and the second most common cause among men.^1^ Research has consistently shown that end-of-life care for people with dementia may be poor with improvements needed in many areas.^2–6^ People with advanced dementia experience a range of symptoms similar to those found in the terminal stages of cancer (including pain, increased risk of cachexia, aspiration, with impaired immunological function), but many lack the capacity required to make decisions about their care and treatment. This has a profound impact on providing the vital components of good end-of-life care.^7^ End-of-life care for this group is further complicated by the fact that many people with dementia are cared for in the community by relatives, homecare workers or care home staff and the general practitioner, resulting in the involvement of multiple care professionals; also, people with dementia often have multiple comorbidities and frailty, meaning that no one specialist is best suited or has a clearly defined role to care for the person with dementia at the end of life, thus potentially undermining continuity of care.^8^

In England, the trend towards increasing hospital deaths among people with a death certificate mention of dementia has reversed with a reciprocal increase in care home deaths, and home and inpatient hospice deaths in dementia are rare.^9^ The European Association for Palliative Care (EAPC) recently published its official position paper defining palliative care for people with dementia as distinct from palliative care for other patient groups,^10^ an important step in the progress of research and service delivery in dementia at the end of life. The White Paper introduced a definition of palliative care that is specific to dementia, through the development of core domains with salient recommendations for each domain. One of the challenges for commissioners and providers of care as well as researchers is how to translate these recommendations into measurable and practically relevant indicators of good or effective care that can inform the development of services and their evaluation. Other recent reviews^11–13^ have focused on the identification of outcome measures for the evaluation of end-of-life care in dementia and in long-term care settings (e.g. QUALIDEM, ADRQL, DQoL), rather than quality indicators. A quality indicator is a measurable aspect of care, generally expressed as a number or percentage and expressed at an aggregate level, often the level of care organisations.^14^ A quality indicator further requires explicit and defined components, including a numerator (e.g. number of patients with improvement in pain score between admission and <48 h), a denominator (e.g. total number of patients for whom pain is scored at admission/48 h) and finally a norm or standard (e.g. at least 50% report improved pain).^15^ Where outcome measures assess how much of a difference we are making, quality indicators assess how ‘good’ a job we are doing;^15^ in this way, quality indicators infer a judgement about the quality of care provided.^16^ It is useful to further differentiate between structure-, process- and outcome-related quality indicators, whereby structure denotes the setting in which care occurs, process denotes what is actually done in giving and receiving the care, and outcome denotes changes in health status or quality of life that can be attributed to the preceding care (including patient and family satisfaction with healthcare, Quality of life of patient, Quality of Life of Family and Loved ones and Quality of Dying Patient).^17–19^

The Supporting Excellence in End of life care in Dementia (SEED) research programme (https://research.ncl.ac.uk/seed/) comprises a series of studies focused on facilitating professionals to deliver better quality care in this area, with the ultimate aim of developing an effective and feasible intervention to support providers and commissioners of community-based end-of-life care in dementia. The aim of this paper is to inform the rigorous evaluation of these types of complex interventions in end-of-life care in dementia by systematically identifying and critically appraising existing quality indicators for palliative care to EAPC domains, thereby using a dementia lens to identify which palliative care indicators have most relevance for people living and dying with dementia in a range of settings.

## Methods

### Data sources and searches

To identify quality indicators with the potential to assess optimal palliative care in older people with dementia, we updated a systematic review of quality indicators for palliative care performed by de Roo et al.^20^ in 2011. We used the search strategy developed by de Roo et al. that identified publications by means of searches in computerised bibliographic databases (i.e. Medline via Ovid, EMBASE via Ovid, PsycINFO via Ovid, and CINAHL via EBSCO) with no limitations with regard to language or year of publication, using keywords and medical-subject headings for palliative care with keywords and medical-subject headings for quality indicators. For Medline search strategy see Supplementary file 1, Appendix 1); the search strategies performed in other databases were similar and are available on request. The search period ran from the inception of the databases to 21 January 2018.

### Inclusion criteria and study selection

Papers were eligible for inclusion if they met the following inclusion criteria developed by de Roo et al.:^20^ (1) the publication describes the development process and/or characteristics of quality indicators developed specifically for palliative care provided by care organisations or professionals and (2) numerators and denominators are defined for the quality indicators, or can be deduced directly from the description of the quality indicators, or performance standards given. English translations of indicators described in the non-English literature could be included if available. Excluded references were (1) editorials, letters to the editor, comments and narrative case reports, (2) indicators focusing on national palliative care policy or the organisation of palliative care at the national level and (3) publications describing the application of existing quality indicators in clinical practice or reviews of several sets of quality indicators without any new developments. All references were screened by two reviewers independently in a two-stage inclusion process. In the first stage, references were screened by two reviewers independently (N.K. and S.A.) by title and abstract. All references deemed eligible for inclusion proceeded to the second selection stage, in which two reviewers (E.L.S. and S.A.) independently examined the remaining references by reading the full texts. Where references selected in the second stage related to conference abstracts and proceedings, we sought to identify any available full publications.

### Methodological assessment

As in de Roo, the quality indicators were assessed methodologically using the Appraisal of Indicators through Research and Evaluation (AIRE) instrument,^21^ by two of the authors (E.L.S. and S.A.) independently for the entire sets of indicators rather than for each quality indicator separately. Standardised scores for each of the three AIRE categories range between 0% and 100%, with a higher score indicating a higher methodological level (see de Roo et al.^20^ for further details regarding score calculation).

### Screening and mapping unique indicators against EAPC framework

To establish indicators of relevance to people dying with or from dementia, we extracted the relevant data from the included literature, including if available, the numerator, denominator, exclusion(s), performance standards, measurement question and/or item, and the type of indicator (i.e. whether the indicator describes a structure, process or outcome of care). We screened and then mapped newly identified indicators as well as indicators previously identified by de Roo et al.,^10^ against the EAPC framework for optimal palliative care in older people with dementia. The framework comprises 11 key domains determined through a rigorous international consensus process, which are (1) applicability of palliative care, (2) person-centred care, communication and shared decision-making, (3) setting care goals and advance planning, (4) continuity of care, (5) prognostication and timely recognition of dying, (6) avoiding overly aggressive, burdensome or futile treatment, (7) optimal treatment of symptoms and providing comfort, (8) psychosocial and spiritual support, (9) family care and involvement, (10) education of the healthcare team and (11) societal and ethical issues. The review was carried out by a multidisciplinary team of experts in palliative and dementia care, with clinical and research expertise in community nursing, old age psychiatry, psychology and primary care. First, indicators were screened by three reviewers independently (E.L.S., C.G. and S.A.) using the exclusion criteria detailed in Table 2. Any discrepancies between reviewers’ exclusions were discussed until agreement was reached. The remaining indicators were then mapped blinded and independently against EAPC domains by two reviewers (E.L.S., S.A.). Any disagreements between reviewers’ classifications were adjudicated by a third reviewer (C.G.). L.R. oversaw the work and provided feedback at each stage of the review process.

## Results

### Search results

A total of 8657 potentially relevant references were found in this 2018 update, after de-duplication. Of these, 12 were publications included in the de Roo update; the remaining de Roo references were not found through the computerised searches, but all figured in the identified publications’ reference lists. We reference tracked all 29 publications included in the de Roo review for any updates, which resulted in the inclusion of 5 new publications^22–26^ describing 3 updated sets of indicators. We also included one of the 12 publications previously identified by de Roo, to assess one of the updated sets.^27^

**Figure 1.**
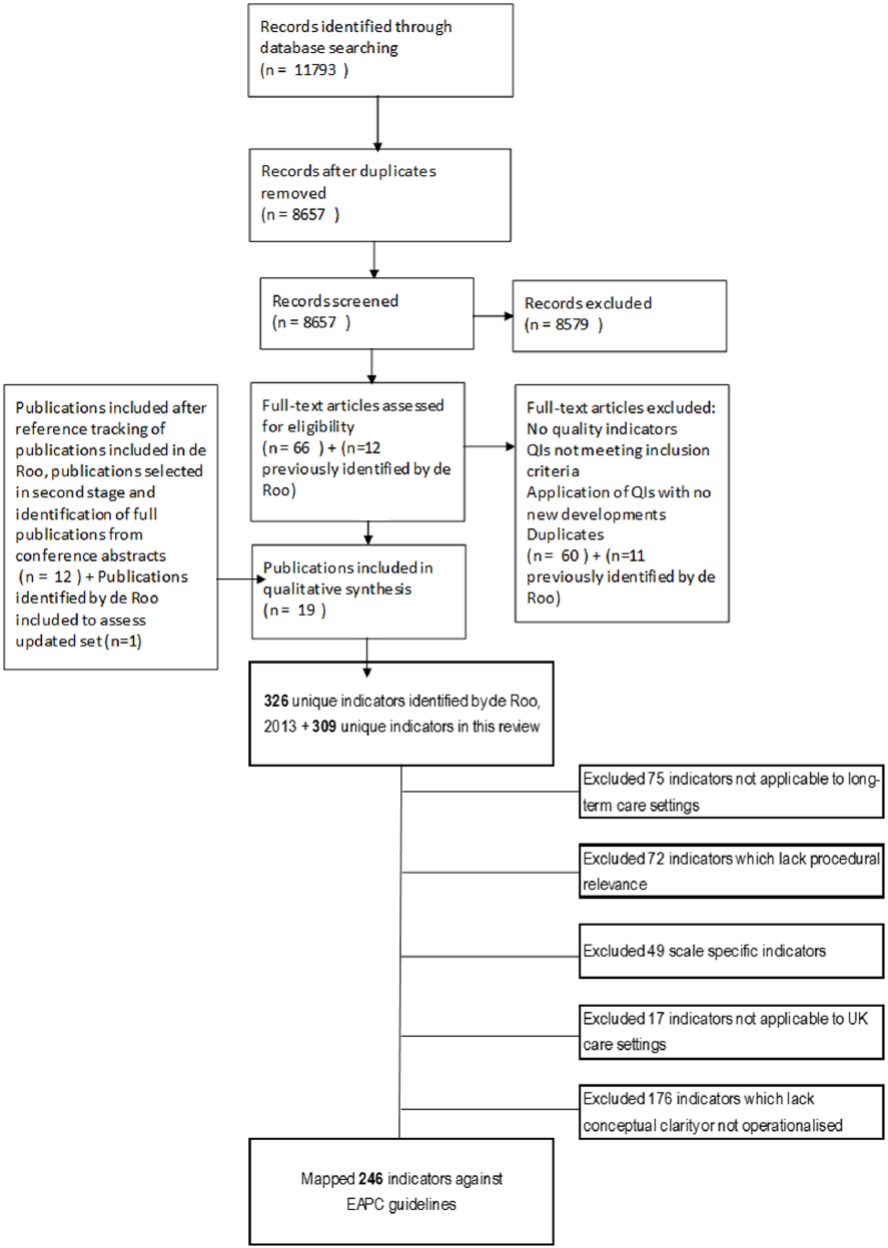
Study selection and screening flow chart.

Furthermore, 66 new publications were retained for a full text read, following screening of titles and abstracts. Six publications that had not previously been identified by de Roo met the inclusion criteria.^28–33^ Seven additional publications^34–40^ not found in the computerised searches were included after reference tracking publications selected for full text review, and identification of full publications from conference proceedings. Finally, two publications were duplicates not picked up by the reference manager software. Hence, a total of 19 publications have been included in this review (see Supplementary file 1, Appendix 3). These 19 publications describe a total of 13 sets of indicators, 10 of which had not been previously identified in the de Roo update; the remaining three are updates of sets previously identified by de Roo (see flow chart presented in Figure 1).

### Methodological characteristics of quality indicators

Publications varied widely as to the level of detail regarding the development of indicator sets. Three sets were attributed the maximum score for the ‘Stakeholder involvement’ and ‘Scientific evidence’ domains’.^22,23,25,36^ Overall, the sets performed less well on the ‘Additional evidence, formulation and usage’ domain; the quality indicators for palliative care ^29^ performed best on this domain with a maximum score of 85%. Individual items on which sets performed poorly included ‘the supporting evidence has been critically appraised’ and ‘a strategy for risk adjustment has been considered and described’.

### Screening

The original review identified 17 sets of quality indicators for palliative care containing 326 unique indicators. We identified a further 10 sets as well as 3 updated sets previously identified by de Roo, containing an additional 309 unique indicators. In total, we screened and mapped a total of 635 indicators using the methods described above.

After screening using the criteria detailed in Table 1, we excluded over 60% of indicators (*n* = 389; 61.3%) because they were not operationalised or lacked conceptual clarity (*n* = 176; 45.2% of excluded indicators), were not applicable to long-term care settings (*n* = 75; 19.3%), lacked procedural relevance (*n* = 72; 18.5%), were specific to a particular scale (*n* = 49; 12.6%) or were not applicable to UK care settings (*n* = 17; 4.4%). Table 2 provides a summary of excluded indicators by exclusion criteria with examples for each (a complete list of indicators is collated in Supplementary file 1, Appendix 2).

**Table 1.** Methodological characteristics of sets of quality indicators using AIRE.

Methodological characteristics	Category 1: Stakeholder involvement (%)	Category 2: Scientific evidence (%)	Category 3: Additional evidence, formulation and usage (%)
CAHPS^22,23,26^	100	100	80
PCOC^24,27^	17	0	41
NICE^25^	100	100	83
Hui et al.^28^	50	78	11
Leemans et al.^29^	56	83	85
QOPI^34,35^	50	6	56
Sanders et al.^36^	100	100	83
Raijmakers et al.^30^	56	83	19
Schnitker and colleagues^37–39^	44	61	70
Sinuff et al.^40^	67	11	2
Van Riet Paap et al.^31^	78	56	26
Walling et al.^32^	61	100	22
Woitha et al.^33^	67	61	11

AIRE: appraisal of indicators through research and evaluation; CAHPS: consumer assessment of healthcare providers and systems; PCOC: Palliative Care Outcomes Collaboration; NICE: national institute for clinical excellence; QOPI: quality oncology practice initiative.

**Table 2.** Summary of excluded indicators (*N* = 389) with examples by exclusion criteria.

Exclusion criteria	% (*n*)	Quality indicator ID number**	Examples (NB: numerator/denominator/performance standard included where available)
Relevance to long-term care settings (we excluded indicators which were not relevant to long-term care settings, that is, indicators which describe procedures in hospice, ICU and hospital settings; we retained hospital-specific indicators when these described procedures are at the interface between community and acute settings that could maintain continuity of care between both)	19.3 (75)	1, 2, 3, 4, 14, 15, 33, 34, 36, 61, 63, 64, 67, 68, 69, 83, 98, 99, 106, 107, 108, 109, 110, 113, 114, 140, 141, 142, 149, 150, 151, 166, 175, 178, 215, 218, 229, 237, 240, 241, 244, 265, 266, 274, 275, 294, 299, 300, 305, 319, 320, 322, 323, 333, 334, 359, 360, 361, 362, 363, 364, 365, 367, 368, 369, 370, 371, 403, 625, 626, 627, 628, 629, 630, 632	(51) Proportion admitted to hospice for less than 3 days. (33) QM for acute hospitals: they (acute hospital providers) have effective mechanisms for identifying those who are at the end of life. Proportion of front-line clinicians who have undergone formal training (99) Regular pain assessment: percentage of 4-h intervals with documentation of pain assessment. Numerator: Number of 4-h intervals for which pain was assessed and documented using an appropriate rating scale Denominator: total number of 4-h intervals on days 0 and 1 (for patients admitted to ICU within the last 24 h). Exclusion: time spent off the unit and no longer in the care of the ICU nurse (e.g. in the operating room); potential exclusions: comatose patients (e.g. Glasgow Coma Score of 2 or 3)
Procedural relevance (we excluded indicators with no procedural relevance to people with advanced dementia, i.e. indicators which describe (1) treatments likely to be considered too aggressive for people with advanced dementia (e.g. chemotherapy) or for which there is no evidence (e.g. counselling for treatment of depression), (2) procedures and investigations likely to be considered too invasive and potentially distressing for people with advanced dementia^41^ and (3) assessments not usually administered to people with advanced dementia (e.g. numeric and/or self-reported assessment tools)	18.5% (72)	59, 111, 112, 115, 119, 128, 130, 132, 133, 136, 143, 146, 155, 156, 157, 159, 160, 167, 168, 173, 174, 176, 177, 179, 180, 181, 182, 183, 187, 188, 189, 190, 199, 202, 203, 204, 205, 206, 207, 219, 222, 223, 224, 225, 261, 269, 276, 307, 315, 405, 492, 493, 494, 495, 496, 497, 498, 499, 500, 501, 502, 503, 504, 505, 506, 507, 508, 509, 510, 633, 634, 635	(132) IF a patient has advanced cancer and receives radiation treatment for painful bone metastases THEN she or he should be offered single-fraction radiation OR there should be documentation of a contraindication to single-fraction treatment. (222) Psychological aspects of care: for patients who screened positive for depression, the percent that received further assessment, counselling or medication treatment. Numerator: number of patients with depression further assessment = ‘Y’ Denominator: # patients with depression screening = Yes (190) Fatigue/anaemia IF a patient with cancer is found to have severe, symptomatic anaemia (haemoglobin <8 g/dL), THEN transfusion with packed red cells should be offered to the patient within 24 h. (146) Dyspnea assessment IF a VE is diagnosed with lung cancer, or cancer metastatic to lung, NYHA Class III–IV CHF, or oxygen-dependent pulmonary disease, THEN a self-reported assessment of dyspnea should be documented in the outpatient chart, BECAUSE dyspnea is common in these conditions, and there are effective treatments for addressing dyspnea
Indicators tied to a specific rating scale (we excluded indicators that were tied to the use of rating scales not specifically developed for use in dementia care, e.g. the Support Team Assessment Schedule (STAS), and the Therapy Impact Questionnaire (TIQ))	12.6% (49)	42, 43, 44, 45, 47, 48, 49, 50, 102, 103, 104, 105, 134, 135, 138, 139, 161, 162, 163, 164, 165, 184, 185, 186, 191, 193, 194, 195, 196, 197, 198, 208, 209, 211, 212, 213, 214, 221, 226, 227, 228, 231, 232, 233, 234, 235, 236, 249, 250	(212) Palliative care services must meet the physical, psychological, social and spiritual needs of patients. Numerator: number of patients with score for patient anxiety (STAS item) of 0–1 during final week of life Denominator: total patients × 100
Relevance to UK care settings (we excluded indicators that were not relevant to UK care settings, i.e. which describe (1) practices that are not standard in the United Kingdom, (2) tools and frameworks no longer in use or structures that have ceased to exist and (3) roles not normally associated with UK healthcare practitioners (e.g. Palliative care services operating at the specific request of the GP, i.e. a gatekeeper role))	4.4% (17)	5, 6, 7, 24, 25, 26, 27, 28, 57, 71, 72, 73, 76, 91, 248, 272, 279	(91)Time in the unstable phase*: time in the unstable phase is calculated as the difference between the phase start date and the phase end date and is analysed by episode type and then occurrence of the unstable phase during the episode. Percentage of patients in their first palliative care phase remain in the unstable phase for less than 7 days. Performance standard: 85% (76) People are treated with dignity and respect and are supported by a common care pathway management approach in the last hours or days of life. Numerator: number of deaths with LCP or equivalent in place. Denominator: total deaths for the same time period. Performance standard: implementation of LCP or equivalent across the organisation (100%) (27) QM for commissioners – Monitoring: Primary Care Trusts must: individual organisations monitor the quality and outputs of end-of-life care and submit relevant information to local and national audits, as measured by proportion of specialist palliative care inpatient facilities (e.g. hospices) which provide data in line with local agreements (87) Discharge planner arranged services required for discharge Numerator: percentage of all cases with documentation that a discharge planner or other hospital personnel arranged any home services necessary
Conceptualisation and operationalisation (i.e. indicators that are defined by more than one numerator/denominator, indicators with descriptions from which it is not possible to deduce numerators, denominators or performance standards)	45.2% (176)	75, 284, 338, 339, 343, 344, 349, 353, 354, 435, 436, 437, 438, 439, 440, 441, 442, 443, 444, 445, 446, 447, 448, 449, 450, 451, 452, 453, 454, 455, 456, 457, 458, 459, 460, 461, 462, 463, 464, 465, 466, 467, 468, 469, 470, 471, 472, 473, 474, 475, 476, 477, 478, 479, 480, 481, 482, 483, 484, 485, 486, 487, 488, 489, 490, 491, 511, 512, 513, 514, 515, 516, 517, 518, 519, 520, 521, 522, 523, 524, 525, 526, 527, 528, 529, 530, 531, 532, 533, 534, 535, 536, 537, 538, 539, 540, 541, 542, 543, 544, 545, 546, 547, 548, 549, 550, 551, 552, 553, 554, 555, 556, 557, 558, 559, 560, 561, 562, 563, 564, 565, 566, 567, 568, 569, 570, 571, 572, 573, 574, 575, 576, 577, 578, 579, 580, 581, 582, 583, 584, 585, 586, 587, 588, 589, 590, 591, 592, 593, 594, 595, 596, 597, 598, 599, 600, 601, 602, 603, 604, 605, 606, 607, 608, 609, 610, 611, 612, 613, 614, 615, 616, 617, 618, 619, 620	(75) Individuals’ preferences and choices are documented and communicated and available at all times of day to all relevant professionals. People’s advance care plans are available to professionals 24 h a day, to ensure they can respond to the wishes of individuals at all times, including out of hours. Ambulance services can routinely and quickly identify people who are known to be approaching the end of life and respect their preferences

* Numbers in parenthesis are quality indicator ID numbers.

** Quality indicator ID numbers 1–326 are indicators previously identified by de Roo, ID numbers 327–635 are indicators identified in this review.

### Mapping

The remaining indicators (*n* = 246; 38.7%) were mapped against EAPC domains 2–11. EAPC Domain 1 (i.e. applicability of palliative care) was not retained as a domain against which to map indicators due to its more conceptual nature.^10^ Two-thirds of quality indicators retained related to processes of care (*n* = 165; 67%) and very few to structures of care (*n* = 11; 4.5%). Close to 30% (*n* = 70; 28.5%) related to outcomes of care, a significant number of which developed as a part of the newly identified quality indicators for palliative care set.^29^ Below, we examine the extent to which indicators are able to form a complete reflection of the domain to which they are ascribed (see Van der Steen et al.^10^ for a detailed description of each domain) and areas in need of further development or not. Table 3 provides a summary of available indicators, key results, gaps and examples for each domain.

**Table 3.** Summary of available indicators (*N* = 246) with key results, examples and identified gaps by EAPC domain.

EAPC domain	% (*n*)	Quality indicator ID number**	Key results and example	Example	Identified gaps
Domain 2. Person-centred care, communication and shared decision-making	9.8% (24)	35, 40, 46, 51, 52, 58, 82, 253, 288, 289, 308, 312, 313, 329, 380, 381, 382, 383, 384, 385, 386, 407, 410, 411	Evidence of (1) an explanation of medical condition to patient including risks and benefits of treatment, documentation of patient’s insight into the disease, and patient/family/caregiver participation in the discussion and development of treatment goals and plans (e.g. preferred place of care), (2) discussion with family about goals of care, regular discussions within the healthcare team around the needs of those approaching the end of life and of a strategy of care, and (3) interdisciplinary meetings with patient and family, joint decisions taken by the care team and family, and discussion of medical condition and goals of treatment with a designated surrogate	(82) Percentage of patients within and among health facilities or systems where evidence exists to confirm patient/family/caregiver participation in the discussion and development of their treatment goals*	No indicators to assess evidence of the management of behaviour that challenges which acknowledges the personhood of people with dementia, recognising that behaviours are a form of communication
Domain 3. Setting care goals and advance planning	12.6% (31)	74, 230, 280, 281, 282, 283, 286, 287, 292, 293, 295, 297, 298, 301, 302, 303, 306, 309, 310, 311, 316, 317, 321, 324, 325, 326, 345, 346, 347, 348, 416	Evidence of (1) mechanisms to assess needs of those approaching end of life (e.g. GSF), (2) ongoing quality of life assessment reflected in the treatment plan, (3) mechanisms to discuss and communicate wishes and treatment preferences (e.g. DNACPR, no tube feeding, no hospital transfer), (4) documentation of a surrogate decision-maker (or lack thereof) and (5) evidence that interventions not wanted by individuals are not performed and individuals are able to die in the location of their preference	(325) Individuals have an agreed care plan. Numerator: number of deceased patients with care plan in place Denominator: total deaths for the same time period	None identified
Domain 4. Continuity of care	9.8% (24)	18, 23, 29, 65, 66, 70, 77, 78, 79, 80, 81, 84, 86, 87, 93, 94, 95, 129, 304, 328, 390, 396, 397, 398	Evidence of (1) nomination of a key worker, (2) of effective communication between services, (3) care plans implemented by all providers consistent with goals of care, (4) essential community services available 24/7, (5) locality wide end-of-life care registers, (6) recognition of care plans across care settings and (7) hospital procedures that maintain continuity of care between acute and community settings (e.g. discharge plans)	(65) Percentage of patients within and among health facilities or systems with evidence that care plan was implemented by all providers consistent with goals of care	None identified
Domain 5. Prognostication and timely recognition of dying	6% (15)	11, 30, 37, 38, 39, 41, 85, 88, 89, 267, 268, 335, 336, 340, 631	Evidence that (1) people approaching end of life are identified and referrals to palliative care made in a timely manner and (2) patients and/or family/caregivers understand and are satisfied with provider communication about prognosis, risks and benefits of treatment and their participation in the development of treatment goals	**(89)** Information and care planning: IF a patient with advanced cancer dies an expected death, THEN s/he should have been referred for palliative care before death (hospital-based or community hospice) OR there should be documentation why there was no referral	None identified
Domain 6. Avoiding overly aggressive, burdensome or futile treatment	4.9% (12)	9, 12, 13, 92, 262, 263, 264, 285, 366, 394, 395, 434	Numbers of unscheduled hospital visits (i.e. A&E or unscheduled admissions) and proportion dying in hospital or at home	(263) Frequency of ER visits: high number of emergency room visits near death may indicate poor quality care Numerator: number of cases with more than 1 ER visit in the last 30 days Denominator: entire cohort	No indicators to assess appropriateness of pharmacological interventions and other care at the end of life (e.g. administration of medication for long term conditions and comorbid diseases, use of restraints, hydration and tube nutrition, use of antibiotics) to avoid overly aggressive, burdensome or futile treatment
Domain 7. Optimal treatment of symptoms and providing comfort	33.3% (82)	20, 60, 62, 90, 96, 97, 100, 101, 116, 117, 118, 120, 121, 122, 123, 124, 125, 126, 127, 131, 137, 144, 145, 147, 148, 152, 153, 154, 158, 169, 170, 171, 172, 192, 200, 201, 210, 216, 217, 220, 318, 331, 337, 341, 342, 350, 351, 352, 355, 356, 357, 358, 372, 373, 374, 375, 376, 391, 392, 393, 406, 408, 409, 412, 413, 417, 418, 419, 421, 422, 423, 424, 425, 426, 427, 428, 432, 433, 621, 622, 623, 624	Evidence of (1) multidisciplinary input, (2) comprehensive palliative care assessments and follow-up to assess the effectiveness of interventions and evidence that symptom relief was achieved and (3) practical arrangements in place to support those dying at home or in a care home and the timely provision of medical aids to ensure preferred place of care	(97) If a terminally ill patient is reported to be in pain, this is addressed by the physician and active attempts are made to reduce pain	No indicators to assess involvement of nursing care specifically (or involvement of dementia care specialist expertise if needed), the use of non-pharmacological interventions (i.e. psycho-social interventions), or the integration of family and professional caregiver views
Domain 8. Psychosocial and spiritual support	5.3% (13)	238, 239, 254, 255, 256, 257, 258, 259, 330, 377, 378, 379, 414	Evidence of (1) assessment of religious affiliation, (2) discussion of spiritual concerns and that spiritual support was offered and (3) documentation of patients’ and families’ emotional reaction to explanation of medical condition.	(256) Spiritual Aspects of Care: percentage of patients with chart documentation of a discussion of spiritual concerns. Numerator: number of patients with spiritual discussion Denominator: Total number of patients	No indicators to assess the quality of the dying environment (e.g. level of comfort)
Domain 9. Family care and involvement	14.6% (36)	53, 54, 55, 56, 242, 243, 245, 246, 247, 251, 252, 260, 270, 271, 273, 277, 278, 290, 291, 296, 314, 327, 332, 387, 388, 389, 399, 400, 401, 402, 404, 415, 420, 429, 430, 431	Evidence (1) that family has been provided with an explanation of the medical condition, the course of disease until death and patient’s impending death, (2) of documentation of family’s preference of explanation of medical condition, of family’s insight of the disease, of configuration of family relationships and key person involved in the patient’s care, (3) of assessment and documentation of carer needs, family’s preferences or expectations, and preferred place of care and (4) a care strategy for family including referral to bereavement services	(246) QM for primary care: They have mechanisms in place to assess and document the needs of carers of those approaching the end of life (Royal College of General Practitioners’’ Supporting Carers), as measured by proportion of carers who have been referred to a carer’s assessment and whose needs have been recorded	None identified
Domain 10. Education of the healthcare team	0.8% (2)	31, 32	Available indicators include evidence of mechanisms in place to identify training needs.	(31) QM for acute hospitals: They have processes in place to identify the training needs of all workers (registered and unregistered) in the hospital that take into account the four core common requirements for workforce development (communication skills, assessment and care planning, advance care planning, and symptom management) as they apply to end of life care, as measured by proportion of workers attending educational programmes related to end of life care for registered workers	Very few indicators mapped to this domain. No indicators to assess level of skill and/or skill mix within the healthcare team
Domain 11. Societal and ethical issues	2.9% (7)	8, 10, 16, 17, 19, 21, 22	Available indicators include evidence of the availability of palliative care for people with dementia	(19) QM for commissioners: Availability of services: people approaching the end of life in care homes have the same level of access to specialist care services as for those who live at home, as measured by proportion of deceased individuals who received specialist palliative care services	Very few indicators mapped to this domain. No indicators to assess levels of collaboration between dementia and palliative care, nor economic or system incentives

* Numbers in parenthesis are quality indicator ID numbers.

** Quality indicator ID numbers 1–326 are indicators previously identified by de Roo, ID numbers 327–635 are indicators identified in this review.

#### Domain 2. Person-centred care, communication and shared decision-making

Indicators mapped to Domain 2 (*n* = 24; 9.8% of indicators mapped to EAPC guidelines) include (1) evidence of an explanation of the medical condition to the patient, the risks and benefits of treatment and documentation of the patient’s insight into the disease, as well as evidence to confirm patient/family/caregiver participation in the discussion and development of treatment goals and plans (e.g. preference for place of care and Do Not Attempt Cardiopulmonary Resuscitation order (DNACPR)). We found no indicators to assess evidence of the management of behaviour that challenges which acknowledges the personhood of people with dementia,^10^ highlighting a potential gap in measuring the application of person-centred care towards and at end of life. Other indicators mapped to this domain include (2) evidence of a discussion with family about the goals of care, as well as regular discussions within the healthcare team around the needs of those approaching end of life and a strategy of care, and finally (3) evidence of interdisciplinary meetings with patients and family, joint decisions taken by the care team and family, and discussion of the medical condition and goals for treatment with a designated surrogate.

#### Domain 3. Setting care goals and advance planning

Indicators mapped to Domain 3 (*n* = 31; 12.6%) include evidence of (1) mechanisms to assess and document the needs of those approaching end of life (e.g. Gold Standards Framework (http://www.goldstandardsframework.org.uk/) or equivalent), (2) ongoing quality-of-life assessment reflected in the treatment plan, (3) mechanisms to discuss, record and communicate the wishes and treatment preferences of those approaching end of life, including withholding or withdrawing life-sustaining treatments (e.g. DNACPR, no tube feeding, no hospital transfer), (3) documentation of a surrogate decision-maker (or lack thereof) and evidence that (5) interventions not wanted by individuals are not performed and individuals are able to die in the location of their preference.

#### Domain 4. Continuity of care

Domain 4 indicators (*n* = 24; 9.8%) include evidence (1) of the nomination of a key worker, (2) of effective communication between services (e.g. between ambulance services and GPs), (3) that care plans are implemented by all providers consistent with goals of care, (4) that essential services in the community are accessible 24/7 to enable people to live and die in the place of their choice, (5) of a locality-wide register of those approaching end of life and (6) of knowledge and recognition of advance care plans across care settings. Hospital-specific indicators that assess evidence of procedures at the interface between community and acute settings (e.g. percentage of all patients with documentation of a discharge plan including statements such as ‘likely to require health services at discharge’ or ‘not expected to survive this admission’ within 4 days of admission) are also mapped to this domain, since they may potentially maintain continuity of care between both.

#### Domain 5. Prognostication and timely recognition of dying

Indicators mapped to Domain 5 (*n* = 15; 6%) include evidence that people approaching the end of life are identified and referrals to palliative care are made in a timely manner (or evidence of documentation why there was not referral), but also evidence that patients and family/caregivers understand and are satisfied with provider communication about prognosis, communication about the risks and benefits of treatment and their participation in the development of treatment goals.

#### Domain 6. Avoiding overly aggressive, burdensome or futile treatment

Indicators mapped to Domain 6, (*n* = 12; 4.9%) measure numbers of unscheduled hospital visits (i.e. A&E or unscheduled admissions) and proportion dying in hospital and at home, which may be used to assess evidence of potentially overly aggressive treatment and of inappropriate hospital transfers at the end of life. Further development is required to fully assess recommendations related to the appropriateness of pharmacological interventions and other care at the end of life in dementia (i.e. administration of medication for long-term conditions and comorbid diseases, use of restraints, hydration and tube nutrition, use of antibiotics and transfers to hospital).

#### Domain 7. Optimal treatment of symptoms and providing comfort

Indicators mapped to Domain 7 (*n* = 82; 33.3%) include evidence of (1) multidisciplinary input, (2) comprehensive palliative care assessments (including pain, dyspnea, depression, emotional distress, delirium/agitation), and follow-up to assess the effectiveness of interventions and evidence that symptom relief was achieved and (3) practical arrangements in place (e.g. equipment and crisis boxes) to support those dying at home or in a care home and the timely provision of medical aids to ensure preferred place of care. No indicators are available to assess evidence of nursing care specifically (or involvement of dementia care specialist expertise if needed), the use of non-pharmacological interventions or the integration of family and professional caregiver views.

#### Domain 8. Psychosocial and spiritual support

Indicators mapped to Domain 8 (*n* = 13; 5.3%) include evidence of assessment of religious affiliation, discussion of spiritual concerns and that spiritual support was offered, as well as documentation of patients’ and families’ emotional reaction to explanation of medical condition. Domain 8 contains an additional recommendation related to the quality of the dying environment for which we found no indicators.

#### Domain 9. Family care and involvement

Indicators mapped to Domain 9 (*n* = 36; 14.6%) include evidence (1) that family has been provided with an explanation of the medical condition, the course of disease until death and patient’s impending death (2) of documentation of family’s preference of explanation of medical condition, of family’s insight of the disease, of configuration of family relationships and key person involved in the patient’s care, (3) evidence of assessment and documentation of carer needs, family’s preferences or expectations, and preferred place of care and (4) evidence of a care strategy for family, including referral to bereavement services.

#### Domain 10. Education of the healthcare team

Very few indicators mapped onto Domain 10 (*n* = 2; 0.8%). Two indicators assess organisational processes for identifying training needs through measurement of the proportion of workers attending educational programmes, but do not reflect recommendations related to skill mix within the healthcare team.

#### Domain 11. Societal and ethical issues

Similarly, few indicators mapped onto Domain 11 (*n* = 7, 2.9%). This is not surprising considering the criteria applied in previous reviews, which excluded indicators focusing on national palliative care policy or the organisation of palliative care at the national level, and on which mapping reported here is based. As such, indicators mapped onto Domain 11 reflect only recommendations related to the availability of palliative care for people with dementia and assess neither levels of collaboration between dementia and palliative care nor economic or systemic incentives.

## Discussion

### Main findings

In this paper, we provide a summary of the quality indicators available to assess optimal palliative care in older people with dementia as defined by the EAPC guidelines, and identify the major gaps related to recommendations for which indicators remain to be developed (see Table 2).

### Strengths and limitations

The assessment of outcomes for end-of-life care in patients with dementia is methodologically difficult, as standard measures of quality and efficacy of care, employed in other medical conditions, may not be appropriate.^42^ Too often, research for people with dementia at the end of life borrows the assumptions of what is good care from cancer models, without analysing which elements are directly transferable, which are not and which can be transferable with modification.^43^ In our study, a multidisciplinary team of experts in palliative and end-of-life care in dementia considered 635 systematically derived indicators for palliative care on an individual basis, using a robust framework that provides clear definitions of aspects of palliative care which are dementia specific. We excluded over half of the existing indicators for palliative care at the screening stage, close to 40% for lack of applicability to populations for whom dementia is the lead condition further complicating the dying trajectory.

Examining processes of care to assess the quality of care relies heavily on evidence that what is assumed to be a good process will produce a good outcome.^17,18^ The methodological assessment of selected indicator sets undertaken here (i.e. using the AIRE instrument) suggests a need for greater critical appraisal of the supporting evidence upon which indicators identified in this review are based. Only then can the presence or absence of certain procedures in specific situations be accepted as evidence of good or bad quality, without the need for further ascertainment.^17^ The assessment of optimal palliative care in older people with dementia is more likely to require a mix of all three types of indicators (i.e. structure, process and outcome) to capture the quality of care at both the service level and from the perspective of the person with dementia and their families and/or carers (see Leemans et al.^44^ for a detailed discussion).

Our work builds on indicators that reflect a biomedical approach to care for people with dementia at the end of life. It is possible that quality indicators based on other approaches, for example, of the person-centeredness of care of older people with dementia^45^ or the quality of life in dementia and/or in long-term care settings^46,47^ may be better suited to assessing optimal end-of-life care in dementia. No indicators were specifically developed for use in settings with no on-site medical and/or nursing staff, which is typical of UK residential care homes and therefore limits the number of indicators applicable to community care settings. Also, quality indicators should adhere as far as possible to some fundamental a priori characteristics,^16^ and the properties of indicators identified here (e.g. validity) remain to be assessed.

#### What this study adds

A major contribution of this study is to have rendered a comprehensive but large list of indicators into a dementia-specific resource, and a framework for future research and implementation of dementia specific end-of-life care. There have been recent calls^43^ to strengthen the theoretical development underpinning new complex interventions designed to improve end-of-life care in dementia – such an approach would benefit quality indicators used to assess the effectiveness of these types of interventions. Questions remain as to (1) the feasibility of developing a set of quality indicators that could be used across the community settings in which older people are living and dying with dementia (e.g. home settings, long-term care settings with and without on-site nursing), (2) where along the dementia trajectory quality indicators for end-of-life care should be introduced and (3) whether quality indicators that are considered important to people with dementia and their families coincide with those habitually used by commissioners (see also Leemans et al.^48^). Overall, a focus on clear and measurable indicators has not so far been able to capture how to apply these over time to reflect what is often an extended dying trajectory involving multiple patient representatives, carers and healthcare professionals at key points. Given the increasing numbers of people who will die with dementia, future work should focus on the development of quality indicators which reflect all aspects of optimal palliative care in dementia, including the use of non-pharmacological interventions, avoidance of overly aggressive, burdensome or futile treatment and skill mix within the healthcare team, potentially building upon indicators developed within person-centred approaches to care aimed at improving comfort and quality of life towards the end of life.

## Supplemental Material

834227_Supplementary_material_for_online_publication_190225_R – Supplemental material for A systematic review and critical appraisal of quality indicators to assess optimal palliative care for older people with dementia Click here for additional data file. Supplemental material, 834227_Supplementary_material_for_online_publication_190225_R for A systematic review and critical appraisal of quality indicators to assess optimal palliative care for older people with dementia by Sarah Amador, Elizabeth L Sampson, Claire Goodman and Louise Robinson in Palliative Medicine
