# Supplementary material for: A systematic review and critical appraisal of quality indicators to assess optimal palliative care for older people with dementia
Source: Palliat Med. 2019 Mar 11;33(4):415–29. doi: 10.1177/0269216319834227 (PMC6439949; doi:10.1177/0269216319834227)
Supplement: 834227_Supplementary_material_for_online_publication_190225_R – Supplemental material for A systematic review and critical appraisal of quality indicators to assess optimal palliative care for older people with dementia [file 834227_Supplementary_material_for_online_publication_190225_R.pdf]

Appendix 1 Search Strategy Medline via Ovid

Database: Ovid MEDLINE(R) Epub Ahead of Print, In-Process & Other Non-Indexed Citations, Ovid MEDLINE(R) Daily and Ovid MEDLINE(R) <1946 to Present>

Search Strategy:

----

1 (palliative or terminal or hospice).ti. (83212)

2 care.ti. (456188)

3 1 and 2 (17970)

4 Palliative Care/ (53626)

5 Terminal Care/ (27304)

6 Advance Care Planning/ (2127)

7 Resuscitation Orders/ (3811)

8 Withholding Treatment/ (11489)

9 Hospice Care/ (6502)

10 Hospices/ (5227)

11 Terminally Ill/ (6636)

12 3 or 4 or 5 or 6 or 7 or 8 or 9 or 10 or 11 (98079)

13 (quality or performance or satisf*).ti. (391398)

14 (indicator* or criter*).tw. or assess*.ti. or measur*.ti. or scale.ti. or validat*.tw. (1857093)

15 13 and 14 (80800)

16 (quality adj1 (criter* or measur* or assess*)).mp. [mp=title, abstract, original title, name of substance word, subject heading word, keyword heading word, protocol supplementary concept word, rare disease supplementary concept word, unique identifier, synonyms] (34943)

17 15 or 16 (103941)

18 Quality Indicators, Health Care/ (14626)

19 17 or 18 (112958)

20 12 and 19 (1238)

21 limit 20 to (addresses or autobiography or bibliography or biography or classical article or clinical conference or clinical study or clinical trial, all or clinical trial, phase i or clinical trial, phase ii or clinical trial, phase iii or clinical trial, phase iv or clinical trial or comparative study or congresses or consensus development conference or consensus development conference, nih or controlled clinical trial or "corrected and republished article" or dataset or dictionary or directory or duplicate publication or english abstract or evaluation studies or festschrift or government publications or guideline or historical article or interactive tutorial or interview or introductory journal article or journal article or lectures or legal cases or legislation or meta analysis or multicenter study or news or newspaper article or observational study or overall or patient education handout or periodical index or personal narratives or portraits or practice guideline or pragmatic clinical trial or published erratum or randomized controlled trial or "research support, american recovery and reinvestment act" or research support, nih, extramural or research support, nih, intramural or research support, non us gov't or research support, us gov't, non phs or research support, us gov't, phs or retracted publication or "retraction of publication" or "review" or "scientific integrity review" or systematic reviews or technical report or twin study or validation studies or video-audio media or webcasts) (1191)

****

Appendix 2 List of quality indicators – Exclusions and retained indicators per EAPC domain

NB Quality indicator ID numbers 1-326 are indicators previously identified by de Roo et al in 2011, ID numbers 327-635 are indicators identified in Amador et al in 2018

QI ID	REFERENCE	QUALITY INDICATOR	NUMERATOR	DENOMINATOR	EXCLUSION(S)	PERFORMANCE STANDARD	SCREEENING	EXCLUSION CRITERIA	EAPC DOMAIN	STRUCTURE, PROCESS or OUTCOME-RELATED
1	Mularski, 2006	Documentation of a policy that allows for unrestricted visitation by family members and friends.	Numerator: Presence of a policy in the ICU that allows for family and friends to spend time in the patient's room regardless of the time of the day. Policy may include restrictions on the number of visitors at one time or restrictions based on disturbance of other patients or family members or disturbance of the functioning of the ICU. Policies may also include provisions for asking family members or friends to wait in the waiting room during procedures	Denominator: ICU	Exclusion: -	Performance standard: -	Excluded	Relevance to long-term care settings	N/A	N/A
2	Nelson, 2006	Family meeting room: dedicated space for meetings between clinicians and ICU families.	Periodic point measurement: presence or absence of room designated for family meetings.	Denominator: -	Exclusion: -	Performance standard: -	Excluded	Relevance to long-term care settings	N/A	N/A
3	NEW ELCQuA, 2011	Families and carers can access designated quiet spaces, and are able to view the deceased in appropriate surroundings.	Numerator: Number of wards with designated quiet area per organisation	Denominator: Total number of wards per organisation	Exclusion: -	Performance standard: 100%	Excluded	Relevance to long-term care settings	N/A	N/A
4	Mularski, 2006	Documentation of a forum for ICU clinicians to review, to discuss, and to debrief the experience of caring for dying patients and their families.	Numerator: Presence of a forum for ICU clinicians to review, discuss, and debrief the experience of caring for dying patients and their families	Denominator: ICU	Exclusion: -	Performance standard: -	Excluded	Relevance to long-term care settings	N/A	N/A
5	Peruselli, 1997	Palliative care service is integrated into the local area health authority and operates at the specific request of the GP and in association with him/her.	Numerator: Number of patients for whom the PCS have had the introductory interview with GP concerning the program objectives	Denominator: Population served x 100	Exclusion: -	Performance standard: 95%	Excluded	Relevance to UK care settings	N/A	N/A

6	Peruselli, 1997	Palliative care service is integrated into the local area health authority and operates at the specific request of the GP and in association with him/her.	Numerator: Number of GPs who have requested the PCS	Denominator: Total number of GPs in area of health care authority x 100	Exclusion: -	Performance standard: 50%	Excluded	Relevance to UK care settings	N/A	N/A
7	Peruselli, 1997	Palliative care service is integrated into the local area health authority and operates at the specific request of the GP and in association with him/her.	Numerator: Number of patients for whom the PCS had have at least 2 interviews with the GP during care process (including preliminary discussion)	Denominator: Population served	Exclusion: -	Performance standard: 50%	Excluded	Relevance to UK care settings	N/A	N/A
8	NEW Quality Markers, 2009 NEW ELCQuA, 2011	QM for commissioners: Availability of services: There is appropriate provision of specialist palliative care services to meet the needs of the population: inpatient services (e.g. specialist palliative care).	Numerator: Numbers of specialist palliative care beds available	Denominator: Per 100,000 patients	Exclusion: -	Performance standard: -	Included	N/A	11-DOMAIN 11 SOCIETAL AND ETHICAL ISSUES	Structure
9	Peruselli, 1997	Home palliative care services reduce admissions to hospital during care period.	Numerator: Number of days in hospital during HPC	Denominator: Total number of days of HPC care x 100	Exclusion: -	Performance standard: 10%	Included	N/A	06-DOMAIN 6 AVOIDING OVERLY AGGRESSIVE, BURDENSOME OR FUTILE TREATMENT	Outcome
10	Peruselli, 1997	The palliative care service responds rapidly to request for care.	Numerator: Number of patients who received initial visits from a team member within 48 hours of request	Denominator: Population served x 100	Exclusion: -	Performance standard: 95%	Included	N/A	11-DOMAIN 11 SOCIETAL AND ETHICAL ISSUES	Outcome
11	Peruselli, 1997	Palliative care services are devised for terminal patients during the final stages of life.	Numerator: Number of patients receiving home care for 7 to 90 days	Denominator: Population served x 100	Exclusion: -	Performance standard: 75%	Included	N/A	05-DOMAIN 5 PROGNOSTICATIO N AND TIMELY RECOGNITION OF DYING	Outcome
12	Peruselli, 1997	Home palliative care enables patients to stay at home until death.	Numerator: Number of patients dying at home	Denominator: Total patients x 100	Exclusion: -	Performance standard: 95%	Included	N/A	06-DOMAIN 6 AVOIDING OVERLY AGGRESSIVE, BURDENSOME OR FUTILE TREATMENT	Outcome
13	Earle,2006	Proportion dying in an acute care setting.	Numerator: Patients who died from cancer in an acute care hospital	Denominator: Patients who died from cancer	Exclusion: -	Performance standard: <17%	Included	N/A	06-DOMAIN 6 AVOIDING OVERLY AGGRESSIVE, BURDENSOME OR FUTILE TREATMENT	Outcome
14	Earle, 2006	Proportion not admitted to hospice.	Numerator: Patients who died from cancer without being admitted to hospice	Denominator: Patients who died from cancer	Exclusion: -	Performance standard: <45%	Excluded	Relevance to long-term care settings	N/A	N/A

15	Earle, 2006	Proportion admitted to hospice for less than 3 days.	Numerator: Patients who died from cancer and spent fewer than three days in hospice	Denominator: Patients who died from cancer who were admitted to hospice	Exclusion: -	Performance standard: <8%	Excluded	Relevance to long-term care settings	N/A	N/A
16	NEW Grunfeld, 2008	Access to care<br>Fair and equitable access to care for patient and family, regardless of financial considerations, indicates good quality care. A high proportion may indicate poor quality care.	Numerator: Number of cases living in a rural area	Denominator: Entire cohort	Exclusion: -	Performance standard: -	Included	N/A	11-DOMAIN 11 SOCIETAL AND ETHICAL ISSUES	Outcome
17	NEW Grunfeld, 2008	Access to palliative care<br>Palliative care will be accessible to all patients and families with a cancer diagnosis, in a timely manner, throughout the entire duration of their disease. A high proportion may indicate good quality care.	Numerator: Number of cases enrolled in palliative care	Denominator: All cases	Exclusion: -	Performance standard: -	Included	N/A	11-DOMAIN 11 SOCIETAL AND ETHICAL ISSUES	Outcome
18	NEW Quality Markers, 2009	QM for commissioners: Availability of services: Essential services are available and accessible in the community 24/7 for all who need them to enable people to live and die in the place of their choice.	Proportion of the local population covered by these services	Numerator: -<br>Denominator: -	Exclusion: -	Performance standard: -	Included	N/A	04-DOMAIN 4 CONTINUITY OF CARE	Structure
19	NEW Quality Markers, 2009	QM for commissioners: Availability of services: People approaching the end of life in care homes have the same level of access to specialist care services as for those who live at home.	Proportion of deceased individuals who received specialist palliative care services	Numerator: -<br>Denominator: -	Exclusion: -	Performance standard: -	Included	N/A	11-DOMAIN 11 SOCIETAL AND ETHICAL ISSUES	Outcome
20	NEW Quality Markers, 2009	QM for district/community nursing services. They have practical arrangements in place to support those dying at home or in care home.	Proportion of cases with equipment, supplies and crisis boxes in place and out of hours sitting services available	Numerator: -<br>Denominator: -	Exclusion: -	Performance standard: -	Included	N/A	07-DOMAIN 7 OPTIMAL TREATMENT OF SYMPTOMS AND PROVIDING COMFORT	Outcome
21	NEW Eagar, 2010	Time from referral to first contact<br>Time from referral to first contact is calculated as the time in days between the referral date and the date of first contact or episode start date (whichever occurs first) and is calculated for all episodes of care and across all settings of care.	Numerator/Denominator: Percentage of patients that are contacted by a member of the clinical team (either face to face or by phone) within 48 hours of referral (including weekends)		Exclusion: -	Performance standard: 90%	Included	N/A	11-DOMAIN 11 SOCIETAL AND ETHICAL ISSUES	Process
22	NEW ELCQuA, 2011	People can access specialist palliative care as needed, regardless of diagnosis or location.	Numerator: Number of specialist palliative care consultants<br><br>(Other measurement option: see Indicator 8)	Denominator: 100,000 population	Exclusion: -	Performance standard: -	Included	N/A	11-DOMAIN 11 SOCIETAL AND ETHICAL ISSUES	Structure

23	NEW Quality Markers, 2009	QM for commissioners: Co-ordination of care across organisational boundaries: A locality-wide register of individuals approaching the end of life is maintained.	Proportion of deceased individuals who entered onto the locality-wide register	Numerator: - Denominator: -	Exclusion: -	Performance standard: -	Included	N/A	04-DOMAIN 4 CONTINUITY OF CARE	Outcome
24	NEW Quality Markers, 2009	QM for commissioners: Monitoring: Primary Care Trusts must: Individual organisations monitor the quality and outputs of end of life care and submit relevant information to local and national audits.	Proportion of general practices which provide data in line with local agreements	Numerator: - Denominator: -	Exclusion: -	Performance standard: -	Excluded	Relevance to UK care settings	N/A	N/A
25	NEW Quality Markers, 2009	QM for commissioners: Monitoring: Primary Care Trusts must: Individual organisations monitor the quality and outputs of end of life care and submit relevant information to local and national audits.	Proportion of care homes which provide data in line with local agreements	Numerator: - Denominator: -	Exclusion: -	Performance standard: -	Excluded	Relevance to UK care settings	N/A	N/A
26	NEW Quality Markers, 2009	QM for commissioners: Monitoring: Primary Care Trusts must: Individual organisations monitor the quality and outputs of end of life care and submit relevant information to local and national audits.	Proportion of hospitals which provide data in line with local agreements	Numerator: - Denominator: -	Exclusion: -	Performance standard: -	Excluded	Relevance to UK care settings	N/A	N/A
27	NEW Quality Markers, 2009	QM for commissioners: Monitoring: Primary Care Trusts must: Individual organisations monitor the quality and outputs of end of life care and submit relevant information to local and national audits.	Proportion of specialist palliative care inpatient facilities (e.g. hospices) which provide data in line with local agreements	Numerator: - Denominator: -	Exclusion: -	Performance standard: -	Excluded	Relevance to UK care settings	N/A	N/A
28	NEW Quality Markers, 2009	QM for commissioners: Monitoring: Primary Care Trusts must: Individual organisations monitor the quality and outputs of end of life care and submit relevant information to local and national audits.	Proportion of community palliative care teams which provide data in line with local agreements	Numerator: - Denominator: -	Exclusion: -	Performance standard: -	Excluded	Relevance to UK care settings	N/A	N/A
29	NEW Quality Markers, 2009	QM for acute hospitals: They ensure that relevant information on patients approaching the end of life is entered into a locality-wide register (where available) or otherwise communicated to other health and social care professionals involved in the patient's care.	Proportion of deceased patients who were recorded in the locality-wide register (locality-wide registers for end of life care are to be piloted starting in 2009)	Numerator: - Denominator: -	Exclusion: -	Performance standard: -	Included	N/A	04-DOMAIN 4 CONTINUITY OF CARE	Process

30	NEW ELCQuA, 2011<br>This indicator suits domain 1 and 8.	People approaching the end of life are identified.	Numerator: Number of deaths on register OR with identified advance care plan<br><br>Numerator: Non-cancer deaths on register OR with an advance care plan identified	Denominator: Total deaths for same catchment and time period<br><br>Denominator: Non-cancer deaths for same catchment for same time period	Exclusion: -<br><br>Exclusion: -	Performance standard: -<br><br>Performance standard: -	Included	N/A	05-DOMAIN 5 PROGNOSTICATIO N AND TIMELY RECOGNITION OF DYING	Outcome
31	NEW Quality Markers, 2009	QM for acute hospitals: They have processes in place to identify the training needs of all workers (registered and unregistered) in the hospital that take into account the four core common requirements for workforce development (communication skills, assessment and care planning, advance care planning, and symptom management) as they apply to end of life care.	Proportion of workers attending educational programs related to end of life care for registered workers	Numerator: -<br>Denominator: -	Exclusion: -	Performance standard: -	Included	N/A	10-DOMAIN 10 EDUCATION OF THE HEALTH CARE TEAM	Structure
32	NEW Quality Markers, 2009	QM for acute hospitals: They have processes in place to identify the training needs of all workers (registered and unregistered) in the hospital that take into account the four core common requirements for workforce development (communication skills, assessment and care planning, advance care planning, and symptom management) as they apply to end of life care.	Proportion of workers attending "foundation" programs in end of life care for non-registered workers who may have to deal with patients approaching the end of life or just after death, or their families and carers	Numerator: -<br>Denominator: -	Exclusion: -	Performance standard: -	Included	N/A	10-DOMAIN 10 EDUCATION OF THE HEALTH CARE TEAM	Structure
33	NEW Quality Markers, 2009	QM for acute hospitals: They (acute hospital providers) have effective mechanisms for identifying those who are at the end of life.	Proportion of front-line clinicians who have undergone formal training	Numerator: -<br>Denominator: -	Exclusion: -	Performance standard: -	Excluded	Relevance to long-term care settings	N/A	N/A
34	Mularski, 2006	Documentation of communication between a physician and a family member or friend of the patient within 24 hours of admission.	Numerator: Patients in the ICU for > 24 hours for whom there is documentation that a physician communicated with a family member or friend of the patient in person or by phone	Denominator: Total number of patients in the ICU for > 24 hours for whom a family member or friend can be identified	Exclusion: Patient for whom no family member or friend can be identified in the first 24 hours	Performance standard: -	Excluded	Relevance to long-term care settings	N/A	N/A

35	Nelson, 2006, Mularski, 2006	Interdisciplinary family meeting: Percentage of patients with documentation that an interdisciplinary family meeting was conducted on or before day 5 of ICU admission.	Numerator: Number of patients who have documentation in the medical record that an interdisciplinary meeting was held with the patient/family on or before day 5 of ICU admission	Denominator: Total number of patient with an ICU length of stay	Exclusion: > 5 days Patients who were not visited by a family member on or before day 5 of ICU admission AND who lack capacity to participate in such a meeting Patients who refused or whose family refused to participate in a family meeting	Performance standard: -	Included	N/A	02-DOMAIN 2 PERSON-CENTRED CARE, COMMUNICATION AND SHARED DECSION MAKING	Process
36	Nelson, 2006	Family information leaflet: Percentage of patients whose families received information leaflet (personally) from ICU team member on or before day 1 of ICU admission and action was documented in the medical record.	Numerator: Number of patients with documentation that family received a written information leaflet from an ICU team member	Denominator: Total number of patients with an ICU length of stay > 5 days	Exclusion: Patients discharged (or transferred out of the ICU) on or before day 1 of ICU admission Patients expired on or before day 1 of ICU admission Patients who were not visited by a family member on or before day 1 of ICU admission	Performance standard: -	Excluded	Relevance to long-term care settings	N/A	N/A
37	Yabroff, 2004	Percentage of patients and family/caregivers within health facilities or systems that understand and are satisfied with provider communication about prognosis.	Numerator: -	Denominator: -	Exclusion: -	Performance standard: -	Included	N/A	05-DOMAIN 5 PROGNOSTICATIO N AND TIMELY RECOGNITION OF DYING	Outcome
38	Yabroff, 2004	Percentage of patients and family/caregivers within and among health facilities or systems that understand and are satisfied with provider communication about risks and benefits or treatment.	Numerator: -	Denominator: -	Exclusion: -	Performance standard: -	Included	N/A	05-DOMAIN 5 PROGNOSTICATIO N AND TIMELY RECOGNITION OF DYING	Outcome
39	Yabroff, 2004	Percentage of patients within and among health facilities or systems where evidence exists to confirm accurate communication about prognosis.	Numerator: -	Denominator: -	Exclusion: -	Performance standard: -	Included	N/A	05-DOMAIN 5 PROGNOSTICATIO N AND TIMELY RECOGNITION OF DYING	Process
40	Yabroff, 2004	Percentage of patients within and among health facilities or systems where evidence exists to confirm accurate communication about risks and benefits of treatment.	Numerator: -	Denominator: -	Exclusion: -	Performance standard: -	Included	N/A	02-DOMAIN 2 PERSON-CENTRED CARE, COMMUNICATION AND SHARED DECSION MAKING	Process

41	Yabroff, 2004	Percentage of patients and family/caregivers within and among health facilities or systems that understand and are satisfied with their participation in the development of treatment goals.	Numerator: -	Denominator: -	Exclusion: -	Performance standard: -	Included	N/A	05-DOMAIN 5 PROGNOSTICATIO N AND TIMELY RECOGNITION OF DYING	Outcome
42	Peruselli, 1997	Palliative care services must meet the physical, psychological, social and spiritual needs of patients.	Numerator: Number of patients with score for patient insight (STAS item) dropped after 8 days of care (if initial score >0)	Denominator: Total patients x 100	Exclusion: -	Performance standard: 75%	Excluded	Scale specific	N/A	N/A
43	Peruselli, 1997	Palliative care services must meet the physical, psychological, social and spiritual needs of patients.	Numerator: Number of patients with score for patient insight (STAS item) of 0-1 during final week of life	Denominator: Total patients x 100	Exclusion: -	Performance standard: 75%	Excluded	Scale specific	N/A	N/A
44	Peruselli, 1997	Palliative care services must meet the physical, psychological, social and spiritual needs of patients.	Numerator: Number of patients with score for family insight (STAS item) dropped after 8 days of care (if initial score >0)	Denominator: Total patients x 100	Exclusion: -	Performance standard: 75%	Excluded	Scale specific	N/A	N/A
45	Peruselli, 1997	Palliative care services must meet the physical, psychological, social and spiritual needs of patients.	Numerator: Number of patients with score for family insight (STAS item) of 0-1 during final week of life	Denominator: Total patients x 100	Exclusion: -	Performance standard: 75%	Excluded	Scale specific	N/A	N/A
46	Peruselli, 1997	Home palliative care services are the result of a joint decision taken by the care team and family.	Numerator: Number of patients for whom the PCS had preliminary interview at the outpatient clinic together with the family	Denominator: Population served x 100	Exclusion: -	Performance standard: 95%	Included	N/A	02-DOMAIN 2 PERSON-CENTRED CARE, COMMUNICATION AND SHARED DECSION MAKING	Process
47	Peruselli, 1997	Palliative care services must meet the physical, psychological, social and spiritual needs of patients.	Numerator: Number of patients with score for communication between professionals (STAS item) dropped after 8 days of care (if initial score >0)	Denominator: Total patients x 100	Exclusion: -	Performance standard: 75%	Excluded	Scale specific	N/A	N/A
48	Peruselli, 1997	Palliative care services must meet the physical, psychological, social and spiritual needs of patients.	Numerator: Number of patients with score for communication between professionals (STAS item) of 0-1 during final week of life	Denominator: Total patients x 100	Exclusion: -	Performance standard: 75%	Excluded	Scale specific	N/A	N/A

49	Peruselli, 1997	Palliative care services must meet the physical, psychological, social and spiritual needs of patients.	Numerator: Number of patients with score for communication professional to patient and family (STAS item) dropped after 8 days of care (if initial score >0)	Denominator: Total patients x 100	Exclusion: -	Performance standard: 75%	Excluded	Scale specific	N/A	N/A
50	Peruselli, 1997	Palliative care services must meet the physical, psychological, social and spiritual needs of patients.	Numerator: Number of patients with score for communication professional to patient and family (STAS item) of 0-1 during final week of life	Denominator: Total patients x 100	Exclusion: -	Performance standard: 75%	Excluded	Scale specific	N/A	N/A
51	NEW Miyashita, 2008 (omitted in Sato, 2008)	Decision making and preference of care: Discussion of strategy of care among physicians and nurses.	Numerator: Number of patients for whom this indicator was documented on admission or within the last 2 weeks of the hospitalization	Denominator: All patients who died (retrospectively identified)	Exclusion: -	Performance standard: -	Included	N/A	02-DOMAIN 2 PERSON-CENTRED CARE, COMMUNICATION AND SHARED DECSION MAKING	Process
52	NEW Miyashita, 2008 (and split up in 2 separate indicators, one for patient and one for family by Sato, 2008)	Decision making and preference of care: Explanation of medical condition to patient.	Numerator: Number of patients for whom this indicator was documented on admission or within the last 2 weeks of the hospitalization	Denominator: All patients who died (retrospectively identified)	Exclusion: -	Performance standard: -	Included	N/A	02-DOMAIN 2 PERSON-CENTRED CARE, COMMUNICATION AND SHARED DECSION MAKING	Process
53	NEW Miyashita, 2008 (and split up in 2 separate indicators, one for patient and one for family by Sato, 2008)	Family care: Explanation of medical condition to family.	Numerator: Number of patients for whom this indicator was documented on admission or within the last 2 weeks of the hospitalization	Denominator: All patients who died (retrospectively identified)	Exclusion: -	Performance standard: -	Included	N/A	09-DOMAIN 9 FAMILY CARE AND INVOLVEMENT	Process
54	NEW Miyashita, 2008	Family care: Explanation to family about course of disease until death.	Numerator: Number of patients for whom this indicator was documented on admission or within the last 2 weeks of the hospitalization	Denominator: All patients who died (retrospectively identified)	Exclusion: -	Performance standard: -	Included	N/A	09-DOMAIN 9 FAMILY CARE AND INVOLVEMENT	Process
55	NEW Miyashita, 2008 ; Sato, 2008	Family care: Explanation to family of patient's impending death.	Numerator: Number of patients for whom this indicator was documented on admission or within the last 2 weeks of the hospitalization	Denominator: All patients who died (retrospectively identified)	Exclusion: -	Performance standard: -	Included	N/A	09-DOMAIN 9 FAMILY CARE AND INVOLVEMENT	Process

56	NEW Miyashita, 2008	Family care: Explanation to family 1 week prior to patient's death.	Numerator: Number of patients for whom this indicator was documented on admission or within the last 2 weeks of the hospitalization	Denominator: All patients who died (retrospectively identified)	Exclusion: -	Performance standard: -	Included	N/A	09-DOMAIN 9 FAMILY CARE AND INVOLVEMENT	Process
57	NEW Grunfeld, 2008	Multidisciplinary care Multidisciplinary care is considered an element of quality cancer care as it provides patients with access to practitioners with different perspectives and skill sets in order to achieve holistic care. Examples may include nursing services, social work, specialised medical services. A high proportion may indicate poor quality care.	Numerator: Sum of GP visits for the entire cohort<br><br>Numerator: Sum of non-GP visits for the entire cohort	Denominator: Sum of physician visits for the entire cohort	Exclusion: -	Performance standard: -	Excluded	Relevance to UK care settings	N/A	N/A
58	NEW Quality Markers, 2009	QM for primary care: They discuss and record the needs of those approaching the end of life at multi-disciplinary team meetings, preferably monthly but a minimum quarterly (refer to the Quality and Outcomes Framework).	Audits of the proportion of deceased individuals who were discussed at a practice-level multidisciplinary meeting in the last year of their lives (this will need to take account of the fact that not all deaths are predictable)	Numerator: -<br>Denominator: -	Exclusion: -	Performance standard: -	Included	N/A	02-DOMAIN 2 PERSON-CENTRED CARE, COMMUNICATION AND SHARED DECSION MAKING	Process
59	NEW Lorenz, 2009	Information and care planning IF a patient with cancer undergoes chemotherapy, THEN before chemotherapy, s/he should be informed about the risks and benefits of treatment, including likely symptoms and side effects, and whether the treatment intent is curative or palliative.	Numerator: -	Denominator: -	Exclusion: -	Performance standard: -	Excluded	Procedural Relevance	N/A	N/A
60	NEW ELCQuA, 2011	People benefit from multi-disciplinary specialist palliative care team input into their care.	Numerator: Number of people on palliative care register discussed in MDM	Denominator: Number of people on palliative care register	Exclusion: -	Performance standard: -	Included	N/A	07-DOMAIN 7 OPTIMAL TREATMENT OF SYMPTOMS AND PROVIDING COMFORT	Process
61	Twaddle, 2007	Documentation of patient status Percentage of all patients with documentation of prognosis, psychosocial symptoms, functional status, and overall symptom distress within 48 hours of admission.	Numerator: -	Denominator: -	Exclusion: -	Performance standard: 90%	Excluded	Relevance to long-term care settings	N/A	N/A

62	Lorenz, 2007	Comprehensive palliative assessment<br>IF a VE dies an expected death with metastatic cancer, oxygen-dependent pulmonary disease, New York Heart Association (NYHA) Class III to IV congestive heart failure (CHF), end-stage (stage IV) renal disease, or dementia, THEN the chart should document pain and other symptoms, spiritual and existential concerns, caregiver burdens and needs for practical assistance, and advance care planning (ACP) within 6 months before death, BECAUSE these concerns are known to be important to patients with advanced illnesses as part of their late-life care.	Numerator: -	Denominator: -	Exclusion: -	Performance standard: -	Included	N/A	07-DOMAIN 7 OPTIMAL TREATMENT OF SYMPTOMS AND PROVIDING COMFORT	Process
63	NEW Schenck, 2010	Structure and Process: Percent of patients who have screening for physical and psychological symptoms during the admission visit.	Numerator: Number of patients with all times=0 (Pain Time=Pain screening date-date of admission<br>Dyspnea Time= Dyspnea screening date-date of admission<br>Nausea Time= Nausea screening date-date of admission<br>Constipation Time= Constipation screening date-date of admission<br>Depression Time= Depression screening date-date of admission<br>Anxiety Time= Anxiety screening date-date of admission)	Denominator: Total # of patients	Exclusion: -	Performance standard: -	Excluded	Relevance to long-term care settings	N/A	N/A

64	NEW Schenck, 2010	Structure and Process: Percent of patients who have comprehensive assessment completed within 5 days of admission.	Numerator: Number of patients with all times<=5	Denominator: Total # of patients (Prognosis Time= Prognosis date-date of admission (Note: if Prognosis Time < 0, set = 0) Functional Status Time= Functional status screening date-date of admission Pain Time=Pain screening date-date of admission Dyspnea Time= Dyspnea screening date-date of admission Nausea Time= Nausea screening date-date of admission Constipation Time= Constipation screening date-date of admission Depression Time= Depression screening date-date of admission Anxiety Time= Anxiety screening date-date of admission Spiritual Time= Spiritual discussion date-date of admission Social Family Time= Family discussion date-date of admission)	Exclusion: -	Performance standard: -	Excluded	Relevance to long-term care settings	N/A	N/A
65	Yabroff, 2004	Percentage of patients within and among health facilities or systems with evidence that care plan was implemented by all providers consistent with goals of care.	Numerator: -	Denominator: -	Exclusion: -	Performance standard: -	Included	N/A	04-DOMAIN 4 CONTINUITY OF CARE	Process
66	Yabroff, 2004	Percentage of patients within and among health facilities or systems with evidence of care planning and provider-provider communication consistent with goals of care.	Numerator: -	Denominator: -	Exclusion: -	Performance standard: -	Included	N/A	04-DOMAIN 4 CONTINUITY OF CARE	Process

67	Mularski, 2006	Documentation that the goals of care and resuscitation status are communicated to the receiving team on transfer of the patient out of the ICU.	Numerator: Total number of patients transferred out of the ICU with documentation that the goals of care and resuscitation status were communicated to the receiving team	Denominator: Total number of patients transferred out of the ICU alive to another service in the hospital or other care facility	Exclusion: Patients who die in the ICU and patients discharged to home from the ICU without home care services	Performance standard: -	Excluded	Relevance to long-term care settings	N/A	N/A
68	Mularski, 2006	Documentation of the goals of care, in the patient chart, within 72 hours.	Numerator: Total number of patients in the ICU for > 72 hours with documentation of the goals of care	Denominator: Total number of patients in the ICU for > 72 hours	Exclusion: -	Performance standard: -	Excluded	Relevance to long-term care settings	N/A	N/A
69	Mularski, 2006	Documentation of a policy that allows for continuity of nursing care for patients with multiple-day stay in the ICU for patients and family members.	Numerator: Presence of a policy in the ICU that supports arranging continuity of nurses for patients who spend > 1 day in the ICU	Denominator: ICU	Exclusion: -	Performance standard: -	Excluded	Relevance to long-term care settings	N/A	N/A
70	Lorenz, 2007	Advance directive and surrogate continuity<br>IF a VE has an advance directive in the outpatient, inpatient, or nursing home medical record, or the patient reports the existence of an advance directive in an interview, and the patient receives care in a second venue, THEN the advance directive should be present in the medical record at the second venue, or documentation should acknowledge its existence and its contents, BECAUSE an advance directive can guide care only if its existence is recognized and its content is known.	Numerator: -	Denominator: -	Exclusion: -	Performance standard: -	Included	N/A	04-DOMAIN 4 CONTINUITY OF CARE	Process
71	NEW Quality Markers, 2009	QM for commissioners: Care in the last days of life: All care providers adopt a common approach to care for people in the last days of life.	Proportion of providers adopting a standardised approach (e.g. the Liverpool Care Pathway or equivalent)	Numerator: -<br>Denominator: -	Exclusion: -	Performance standard: -	Excluded	Relevance to UK care settings	N/A	N/A
72	NEW Quality Markers, 2009	QM for commissioners: Care in the days after death: All providers have appropriate processes in place for verification and certification of death, including viewing of the body and return of personal property.	Proportion of providers using the care after death module of the Liverpool Care Pathway (or equivalent)	Numerator: -<br>Denominator: -	Exclusion: -	Performance standard: -	Excluded	Relevance to UK care settings	N/A	N/A

73	NEW Quality Markers, 2009	QM for commissioners: Care in the days after death: All providers have appropriate processes in place for verification and certification of death, including viewing of the body and return of personal property.	Proportion of individuals for whom the care after death module of the Liverpool Care Pathway (or equivalent) was used (including those who died suddenly)	Numerator: -<br>Denominator: -	Exclusion: -	Performance standard: -	Excluded	Relevance to UK care settings	N/A	N/A
74	NEW Quality Markers, 2009	QM for district/community nursing services. They have mechanisms in place to assess and document the needs of those approaching the end of life (e.g. use of the Gold Standards Framework or equivalent), and to discuss, record and, (where appropriate) communicate the wishes and preferences of those approaching the end of life (advance care planning).	Proportion of nursing services that have adopted an approach to end of life care management (e.g. the Gold Standards Framework or equivalent)	Numerator: -<br>Denominator: -	Exclusion: -	Performance standard: -	Included	N/A	03-DOMAIN 3 SETTING CARE GOALS AND ADVANCE PLANNING	Structure
75	NEW ELCQuA, 2011	Individuals' preferences and choices are documented and communicated and available at all times of day to all relevant professionals.<br><br>People's advance care plans are available to professionals 24 hours a day, to ensure they can respond to the wishes of individuals at all times including out of hours.<br><br>Ambulance services can routinely and quickly identify people who are known to be approaching the end of life and respect their preferences.	Numerator: Number of people who died who were recorded on an electronic end of life care register or equivalent + number who declined to have their record uploaded		Exclusion: -	Performance standard: All patients approaching the end of life offered entry onto locality register, including DNACPR wishes and preferred place of care/death	Excluded	Conceptualisation and/or Operationalisation	N/A	N/A
76	NEW ELCQuA, 2011	People are treated with dignity and respect and are supported by a common care pathway management approach in the last hours or days of life.	Numerator: Number of deaths with LCP or equivalent in place<br><br>Numerator: Completed and available LCP goals documentation fields (scored either as 'achieved' or 'variance') for a consecutive sample of end of life patients	Denominator: Total deaths for same time period<br><br>Denominator: Number of available and applicable LCP goals documentation fields for consecutive sample of the same number of end of life patients	Exclusion: -<br><br>Exclusion: -	Performance standard: Implementation of LCP or equivalent across the organisation (100%)	Excluded	Relevance to UK care settings	N/A	N/A
77	NEW Quality Markers, 2009	QM for community hospitals: They (all community hospitals which provide relevant services) nominate a key worker for each	Audits of the proportion of patients approaching the end of life with a documented key worker	Numerator: -<br>Denominator: -	Exclusion: -	Performance standard: -	Included	N/A	04-DOMAIN 4 CONTINUITY OF CARE	Process

		patient approaching the end of life if required.								
78	NEW Quality Markers, 2009	QM for care homes: All care home providers that provide services to people approaching the end of life (care homes need to consider end of life care from the point of view of residents who may have a year or more to live, rather than days or weeks). They nominate a key worker for each patient approaching the end of life if required.	Audits of the proportion of patients approaching the end of life with a documented key worker	Numerator: -<br>Denominator: -	Exclusion: -	Performance standard: -	Included	N/A	04-DOMAIN 4 CONTINUITY OF CARE	Process
79	NEW Quality Markers, 2009	QM for specialist palliative care inpatient facilities e.g. hospices: They nominate a key worker for each patient approaching the end of life if required.	Audits of the proportion of patients approaching the end of life with a documented key worker	Numerator: -<br>Denominator: -	Exclusion: -	Performance standard: -	Included	N/A	04-DOMAIN 4 CONTINUITY OF CARE	Process
80	NEW Quality Markers, 2009	QM for district/community nursing services. They nominate a key worker for each patient approaching the end of life if required.	Audits of the proportion of patients approaching the end of life with a documented key worker	Numerator: -<br>Denominator: -	Exclusion: -	Performance standard: -	Included	N/A	04-DOMAIN 4 CONTINUITY OF CARE	Process
81	NEW ELCQuA, 2011	People are able to contact a dedicated key worker at all times.	Numerator: Number of patients/service users who died with a documented key worker	Denominator: Total deaths for same time period	Exclusion: -	Performance standard: -	Included	N/A	04-DOMAIN 4 CONTINUITY OF CARE	Outcome
82	Yabroff, 2004	Percentage of patients within and among health facilities or systems where evidence exists to confirm patient/family/caregiver participation in the discussion and development of their treatment goals.	Numerator: -	Denominator:-	Exclusion: -	Performance standard: -	Included	N/A	02-DOMAIN 2 PERSON-CENTRED CARE, COMMUNICATION AND SHARED DECSION MAKING	Process
83	National Hospice and Palliative Care Organization (NHPCO), 2006<br>Brown University, 2006	Provide coordination of care (Family evaluation of hospice care)<br>Questions:<br>F1: How often did someone from the hospice team give confusing or contradictory information about the patient's medical treatment?<br>F2: While under the care of hospice, was there always one nurse who was identified as being in charge of the patient's overall care?<br>F3: Was there any problem with hospice doctors or nurses not knowing enough about the patient's medical history to provide the best possible care?	Numerator: Sum of 1 response to survey instrument by family member of deceased patient	Denominator: Number of items (=3)	Exclusion: Patients who are not enrolled in a hospice program or have disenrolled from a hospice program. Live discharged are excluded	Performance standard: -	Excluded	Relevance to long-term care settings	N/A	N/A

84	Twaddle, 2007	Patient/family meeting Percentage of all cases with documentation that a patient/family meeting (i.e. meeting between patient/family and members of the health care team to discuss the patient's treatment preferences or the plans for discharge disposition) occurred during the first week of the hospital stay.	Numerator: -	Denominator: -	Exclusion: -	Performance standard: 90%	Included	N/A	04-DOMAIN 4 CONTINUITY OF CARE	Process
85	Peruselli, 1997	Satisfaction for patients and families is crucial to palliative care services, which considers clients to be their central focus.	Numerator: Number of responses to questionnaire items answered "excellent" by both patient and family	Denominator: Total number of responses x 100	Exclusion: -	Performance standard: 75%	Included	N/A	05-DOMAIN 5 PROGNOSTICATIO N AND TIMELY RECOGNITION OF DYING	Outcome
86	Twaddle, 2007	Documentation of discharge plan Percentage of all patients with documentation of discharge plan (i.e. early documentation of statements such as 'likely to require health services at discharge' or 'not expected to survive this admission') within 4 days of admission.	Numerator: -	Denominator: -	Exclusion: -	Performance standard: 90%	Included	N/A	04-DOMAIN 4 CONTINUITY OF CARE	Process
87	Twaddle, 2007	Discharge planner arranged services required for discharge Percentage of all cases with documentation that a discharge planner or other hospital personnel arranged any home services necessary.	Numerator: -	Denominator: -	Exclusion: -	Performance standard: 90%	Included	N/A	04-DOMAIN 4 CONTINUITY OF CARE	Process
88	[NEW] Grunfeld, 2008	Enrollment in palliative care within 6 months of death A high proportion may indicate poor quality care. Palliative care should be accessible to all patients and families with a cancer diagnosis, in a timely manner, throughout the entire duration of their disease.	Numerator: Number of cases enrolled in palliative care within six months prior to death	Denominator: All cases enrolled in palliative care	Exclusion: -	Performance standard: -	Included	N/A	05-DOMAIN 5 PROGNOSTICATIO N AND TIMELY RECOGNITION OF DYING	Outcome
89	[NEW] Lorenz, 2009	Information and care planning IF a patient with advanced cancer dies an expected death, THEN s/he should have been referred for palliative care before death (hospital-based or community hospice) OR there should be documentation why there was no referral.	Numerator: -	Denominator: -	Exclusion: -	Performance standard: -	Included	N/A	05-DOMAIN 5 PROGNOSTICATIO N AND TIMELY RECOGNITION OF DYING	Process
90	[NEW] Claessen, 2011	Generic aspects: Percentage of patients who receive medical aids soon enough Receiving medical aids soon enough is an important quality aspect of palliative care.	Numerator: The number of patients who indicate that they receive medical aids soon enough	Denominator: The total number of patients among whom this quality	Exclusion: Patients with moderate to (very) severe cognitive impairments,	Performance standard: -	Included	N/A	07-DOMAIN 7 OPTIMAL TREATMENT OF SYMPTOMS	Outcome

				indicator was measured	young children, and comatose and deeply sedated patients				AND PROVIDING COMFORT	
91	NEW Eagar, 2010	Time in the unstable phase<br>Time in the unstable phase is calculated as the difference between the phase start date and the phase end date and is analyzed by episode type and then occurrence of the unstable phase during the episode.	Percentage of patients in their first palliative care phase remain in the unstable phase for less than 7 days<br><br>Percentage of patients in a subsequent palliative care phase who remain in the unstable phase for less than 7 days		Exclusion: -	Performance standard: 85%<br><br>Performance standard: 90%	Excluded	Relevance to UK care settings	N/A	N/A
92	NEW ELCQuA, 2011	Care for individuals is coordinated across organisational boundaries 24 hours a day, seven days a week.	Numerator: Number of emergency admissions for patients in last 12 months of life	Denominator: Total deaths for same time period	Exclusion: -	Performance standard: -	Included	N/A	06-DOMAIN 6 AVOIDING OVERLY AGGRESSIVE, BURDENSOME OR FUTILE TREATMENT	Structure
93	NEW ELCQuA, 2011	People can be transferred quickly and sensitively to their preferred location of care.	Numerator: Sum of delay (days) for EoL patients awaiting transfer from a) home, and b) acute sector to preferred location	Denominator: Total number of deaths where a transfer is requested	Exclusion: -	Performance standard: -	Included	N/A	04-DOMAIN 4 CONTINUITY OF CARE	Outcome
94	NEW ELCQuA, 2011	Ambulances services inform GPs where they have transferred a person who is at the end of life to a hospital.	Numerator: Number of transfers for people on EOLC register where the GP is informed	Denominator: Total number of transfers for people on EOLC register	Exclusion: -	Performance standard: -	Included	N/A	04-DOMAIN 4 CONTINUITY OF CARE	Process
95	NEW ELCQuA, 2011	All organisations communicate effectively with people's GPs around end of life decisions and inform the general practice within 24 hours when a person dies.	Numerator: Number of deaths where the GP is informed within 24 hours	Denominator: Total number of deaths	Exclusion: -	Performance standard: GPs receive timely notification of all deaths	Included	N/A	04-DOMAIN 4 CONTINUITY OF CARE	Process
96	Keay, 1994	If a patient had pain, this is followed to assess results of intervention and pain is reduced.	Numerator: -	Denominator:-	Exclusion: Intractable pain ever after consultation	Performance standard: > 80%	Included	N/A	07-DOMAIN 7 OPTIMAL TREATMENT OF SYMPTOMS AND PROVIDING COMFORT	Outcome
97	Keay, 1994	If a terminally ill patient is reported to be in pain, this is addressed by the physician and active attempts are made to reduce pain.	Numerator: -	Denominator: -	Exclusion: Minor pain symptoms in cognitively intact patients	Performance standard: 100%	Included	N/A	07-DOMAIN 7 OPTIMAL TREATMENT OF SYMPTOMS AND PROVIDING COMFORT	Outcome

98	Nelson, 2006, Mularski, 2006	Regular pain assessment: percentage of 4 hour intervals with documentation of pain assessment.	Numerator: Number of 4 hour intervals for which pain was assessed and documented using an appropriate rating scale	Denominator: Total number of 4 hour intervals on days 0 and 1 (for patients admitted to ICU within the last 24 hours)	Exclusion: Time spend off the unit and no longer in the care of the ICU nurse (e.g. in the operating room); potential exclusions: comatose patients patients (e.g. Glasgow Coma Score of 2 or 3)	Performance standard: -	Excluded	Relevance to long-term care settings	N/A	N/A
99	Nelson, 2006, Mularski, 2006	Optimal pain management: percentage of 4 hour intervals with documented pain score < 3 on 1-10 scale.	Numerator: Number of 4 hour intervals for which pain score was < 3 on 1-10 scale (or equivalent)	Denominator: Total number of 4 hour intervals on days 0 and 1 (for patients admitted to ICU within the last 24 hours)	Exclusion: Time spend off the unit and no longer in the care of the ICU nurse (e.g. in the operating room)	Performance standard: -	Excluded	Relevance to long-term care settings	N/A	N/A
100	Lorenz, 2007 NEW Adapted by van der Ploeg, 2008 (see indicator 101)	Management of emergent pain and obstruction IF a VE who was conscious during the last 7 days of life died an expected death, THEN the medical record should contain documentation about presence or absence of pain during the last 7 days of life, BECAUSE pain is common at the end of life and can be effectively treated.	Numerator: -	Denominator:-	Exclusion: -	Performance standard: -	Included	N/A	07-DOMAIN 7 OPTIMAL TREATMENT OF SYMPTOMS AND PROVIDING COMFORT	Process
101	NEW van der Ploeg, 2008 (Based on Lorenz, 2007, see indicator 100)	IF a vulnerable elder who was conscious during the last 7 days of life died an expected death, THEN the general practitioners record should document a pain policy (including interventions).	Numerator: -	Denominator: -	Exclusion: -	Performance standard: -	Included	N/A	07-DOMAIN 7 OPTIMAL TREATMENT OF SYMPTOMS AND PROVIDING COMFORT	Process
102	Peruselli, 1997	Palliative care services must meet the physical, psychological, social and spiritual needs of patients.	Numerator: Number of patients with score for pain control (STAS item) dropped after 8 days of care (if initial score >0)	Denominator: Total patients x 100	Exclusion: -	Performance standard: 75%	Excluded	Scale specific	N/A	N/A
103	Peruselli, 1997	Palliative care services must meet the physical, psychological, social and spiritual needs of patients.	Numerator: Number of patients with score for pain control (STAS item) of 0-1 during final week of life	Denominator: Total patients x 100	Exclusion: -	Performance standard: 75%	Excluded	Scale specific	N/A	N/A
104	Peruselli, 1997	Palliative care services must meet the physical, psychological, social and spiritual needs of patients.	Numerator: Number of patients with global scores for pain (TIQ scale) dropped after 8 days of care (if initial score on the same scale > 25)	Denominator: Total patients x 100	Exclusion: -	Performance standard: 75%	Excluded	Scale specific	N/A	N/A

105	Peruselli, 1997	Palliative care services must meet the physical, psychological, social and spiritual needs of patients.	Numerator: Number of patients with global scores for pain (TIQ scale) not increased over initial score during final week of care (if initial score on the same scale > 25)	Denominator: Total patients x 100	Exclusion: -	Performance standard: 75%	Excluded	Scale specific	N/A	N/A
106	National Hospice and Palliative Care Organization (NHPCO), 2006 Brown University, 2006	Family evaluation of hospice care: Symptom management Questions: B1: While under the care of hospice, did the patient have pain or take medicine for pain? B2: How much medicine did the patient receive for his/her pain?	Numerator: Those who received too much or too little help concerning pain	Denominator: Those who experience pain	Exclusion: Patients who are not enrolled in a hospice program or have disenrolled from a hospice program. Live discharged are excluded	Performance standard: -	Excluded	Relevance to long-term care settings	N/A	N/A
107	National Hospice and Palliative Care Organization (NHPCO), 2006 Brown University, 2006	Comfortable dying	Numerator: Patients whose pain was brought under control within 48 hours of admission to hospice	Denominator: Patients who were uncomfortable because of pain on admission to hospice	Exclusion: -	Performance standard: -	Excluded	Relevance to long-term care settings	N/A	N/A
108	Twaddle, 2007	Pain assessment Percentage of all patients with documentation of pain assessment within 48 hours of admission.	Numerator: -	Denominator: -	Exclusion: -	Performance standard: 90%	Excluded	Relevance to long-term care settings	N/A	N/A
109	Twaddle, 2007	Use of a quantitative pain rating scale Percentage of patients with pain evaluated according to a numeric or other validated pain scale.	Numerator: -	Denominator: Patients reporting pain within 48 hours of admission	Exclusion: -	Performance standard: 90%	Excluded	Relevance to long-term care settings	N/A	N/A
110	Twaddle, 2007	Reduction or relief of pain Percentage of patients with pain relieved or reduced (i.e. pain score of 3 or less) within 48 hours of admission.	Numerator: -	Denominator: Patients reporting pain	Exclusion: -	Performance standard: 90%	Excluded	Relevance to long-term care settings	N/A	N/A
111	Lorenz, 2007	Management of emergent pain and obstruction IF a VE with end-stage metastatic cancer is treated with opiates for pain, THEN the medical record should document a plan for management of worsening or emergent pain, BECAUSE increasing symptom burden is common with the progression of cancer and significantly impairs patient quality of life.	Numerator: -	Denominator: -	Exclusion: -	Performance standard: -	Excluded	Procedural Relevance	N/A	N/A

112	Lorenz, 2007	Management of emergent pain and obstruction<br>IF a VE with end-stage metastatic cancer has obstructive gastrointestinal symptoms, THEN the medical record should document a plan for management of worsening or emergent nausea and vomiting, BECAUSE increasing symptom burden is common with these conditions and significantly impairs patient quality of life.	Numerator: -	Denominator: -	Exclusion: -	Performance standard: -	Excluded	Procedural Relevance	N/A	N/A
113	Mularski, 2006	Documentation of respiratory distress assessment (for non-ventilated patient) or patient-ventilator dyssynchrony (for ventilated patients) every 8 hours.	Numerator: Total number of 8-hour periods during the proportion of the 24-hour day that a patient is in the ICU or under care of the ICU nurse for which dyspnea/ dyssynchrony is assessed and recorded using a quantitative rating scale	Denominator: Total number of 8-hour periods that a patient is in the ICU during the proportion of the 24-hour day that a patient is in the ICU or under care of the ICU nurse	Exclusion: Time Spend off the unit and no longer in the care of the ICU nurse (e.g. in the operating room)	Performance standard: -	Excluded	Relevance to long-term care settings	N/A	N/A
114	Mularski, 2006	Treatment of or management plan for respiratory distress (for non-ventilated patients) or patient-ventilator dyssynchrony (for ventilated patients) that is assessed as >3 on a 1-10 scale or greater than mild on other scales with reassessment after treatment.	Numerator: Total number of 8-hour periods during the proportion of the 24-hour day that a patient is in the ICU or under care of the ICU nurse for which respiratory distress/dyssynchony is assessed as >3 (or greater than mild) and there is a documented treatment/management plan provided and documented reassessment within 2 hours after treatment/management plan	Denominator: Total number of 8-hour periods during the proportion of the 24-hour day that a patient is in the ICU or under care of the ICU nurse for which respiratory distress/dyssynchony is assessed as >3 (or greater than mild)	Exclusion: Time spend off the unit and no longer in the care of the ICU nurse (e.g. in the operating room)	Performance standard: -	Excluded	Relevance to long-term care settings	N/A	N/A
115	[NEW] Grunfeld, 2008	Radiotherapy for uncontrolled bone pain for bony metastases<br>Providing patients with radiotherapy for bony metastases improves pain management and improves quality of life. A high proportion may indicate good quality care.	Numerator: Number of cases who received palliative radiation to the bones	Denominator: The entire cohort	Exclusion: -	Performance standard: -	Excluded	Procedural Relevance	N/A	N/A

116	NEW Miyashita, 2008<br>Sato, 2008	Symptom control: Presence or absence of pain	Numerator: Number of patients for whom this indicator was documented on admission or within the last 2 weeks of the hospitalization	Denominator: All patients who died (retrospectively identified)	Exclusion: -	Performance standard: -	Included	N/A	07-DOMAIN 7 OPTIMAL TREATMENT OF SYMPTOMS AND PROVIDING COMFORT	Outcome
117	NEW Miyashita, 2008<br>Sato, 2008	Symptom control: Degree of pain	Numerator: Number of patients for whom this indicator was documented on admission or within the last 2 weeks of the hospitalization	Denominator: All patients who died (retrospectively identified)	Exclusion: -	Performance standard: -	Included	N/A	07-DOMAIN 7 OPTIMAL TREATMENT OF SYMPTOMS AND PROVIDING COMFORT	Outcome
118	NEW Miyashita, 2008<br>Sato, 2008	Symptom control: Physician's prescription order for pain management	Numerator: Number of patients for whom this indicator was documented on admission or within the last 2 weeks of the hospitalization	Denominator: All patients who died (retrospectively identified)	Exclusion: -	Performance standard: -	Included	N/A	07-DOMAIN 7 OPTIMAL TREATMENT OF SYMPTOMS AND PROVIDING COMFORT	Process
119	NEW Sato, 2008	Symptom control: Physician's prescription order for first and second line pain management	Numerator: Number of patients for whom this indicator was documented on admission or within the last 2 weeks of the hospitalization	Denominator: All patients who died (retrospectively identified)	Exclusion: -	Performance standard: -	Excluded	Procedural Relevance	N/A	N/A
120	NEW Schenck, 2010	Physical Aspects of Care: Percent of patients screened for pain during the admission visit.	Numerator: Number of patients with pain time=0	Denominator: # of patients (Pain time = pain screening date – date of admission)	Exclusion: -	Performance standard: -	Included	N/A	07-DOMAIN 7 OPTIMAL TREATMENT OF SYMPTOMS AND PROVIDING COMFORT	Process
121	NEW Schenck, 2010	Physical Aspects of Care: For patients who screened positive for pain, the percent with clinical assessment within 1 day of screening.	Numerator: Number of patients with (0<=Clinical Pain Time<=1)	Denominator: Patients with pain (Clinical Pain Time= screening date – pain assessment date)	Exclusion: -	Performance standard: -	Included	N/A	07-DOMAIN 7 OPTIMAL TREATMENT OF SYMPTOMS AND PROVIDING COMFORT	Process
122	NEW Schenck, 2010	Physical Aspects of Care: For patients who screened positive for pain, the percent with any treatment within 1 day of screening.	Numerator: Number of patients with (0<=Pain Treatment Time<=1) and (pain treatment="Y") (Pain Treatment Time = date of treatment – pain assessment date)	Denominator: Patients with pain	Exclusion: -	Performance standard: -	Included	N/A	07-DOMAIN 7 OPTIMAL TREATMENT OF SYMPTOMS AND PROVIDING COMFORT	Process

123	NEW Schenck, 2010	Physical Aspects of Care: For patients who screened positive for pain, the percent who have an order for regularly scheduled (not PRN) pain medication within 1 day of screening.	Numerator: Number of patients with (0<=Pain Treatment Time<=1) and (Treatment=1) (Pain Treatment time = date of treatment – pain assessment date Treatment = 1 if type of treatment equals "Scheduled medication, opioid" or "Scheduled medication, non-opioid")	Denominator: Number of patients with pain	Exclusion: -	Performance standard: -	Included	N/A	07-DOMAIN 7 OPTIMAL TREATMENT OF SYMPTOMS AND PROVIDING COMFORT	Process
124	NEW Schenck, 2010	Physical Aspects of Care: For patients who screened positive for pain, the percent with improvement within 1 day of screening.	Numerator: Number of patients with (0<=Improvement Time<=1) and (Improvement=1) (Improvement Time = Second Pain assessment date-Pain assessment date)	Denominator: Patients with pain	Exclusion: -	Performance standard: -	Included	N/A	07-DOMAIN 7 OPTIMAL TREATMENT OF SYMPTOMS AND PROVIDING COMFORT	Outcome
125	NEW Schenck, 2010	Physical Aspects of Care: For patients who screened positive for pain, the percent whose pain was at a comfortable level within 2 days of screening (patient report of comfort or mild pain based on standard pain rating scale).	Numerator: Number of patients with (0<=Improvement Time<=2) and (Comfort =1) (Improvement Time = Second Pain assessment date-Pain assessment date Comfort =1 if patient reports comfort or mild pain based on standard pain rating scale)	Denominator: Patients with pain	Exclusion: -	Performance standard: -	Included	N/A	07-DOMAIN 7 OPTIMAL TREATMENT OF SYMPTOMS AND PROVIDING COMFORT	Outcome
126	NEW Schenck, 2010	Physical Aspects of Care: Percent of patients with cognitive and language problems receiving pain assessment appropriate to communication needs.	Numerator: (Number of patients with dementia or confusion and pain assessment = observational) + (number of patients who are deaf or non-English speaking with pain assessment = translated materials)	Denominator: Number of patients with dementia, confusion, deafness or non-English	Exclusion: -	Performance standard: -	Included	N/A	07-DOMAIN 7 OPTIMAL TREATMENT OF SYMPTOMS AND PROVIDING COMFORT	Process
127	NEW Schenck, 2010	Care for the Imminently Dying: Percent of patients who had moderate to severe pain on a standard rating scale at any time in the last week of life.	Numerator: Number of patients with Pain in last week= "Moderate" or "Severe"	Denominator: Number of patients who died	Exclusion: -	Performance standard: -	Included	N/A	07-DOMAIN 7 OPTIMAL TREATMENT OF SYMPTOMS AND PROVIDING COMFORT	Outcome

128	NEW Lorenz, 2009	Pain: IF a cancer patient has a cancer-related outpatient visit THEN there should be screening for the presence or absence and intensity of pain using a numeric pain score.	Numerator: -	Denominator: -	Exclusion: -	Performance standard: -	Excluded	Procedural Relevance	N/A	N/A
129	NEW Lorenz, 2009	Pain: IF a cancer patient is admitted to a hospital THEN there should be screening for the presence or absence of pain.	Numerator: -	Denominator: -	Exclusion: -	Performance standard: -	Included	N/A	04-DOMAIN 4 CONTINUITY OF CARE	Process
130	NEW Lorenz, 2009	Pain: IF a patient with cancer pain is started on a long-acting opioid formulation, THEN a short-acting opioid formulation for breakthrough pain should also be provided.	Numerator: -	Denominator: -	Exclusion: -	Performance standard: -	Excluded	Procedural Relevance	N/A	N/A
131	NEW Lorenz, 2009	Pain: IF a patient's outpatient cancer pain regimen is changed, THEN there should be an assessment of the effectiveness of treatment at or before the next outpatient visit with that provider or at another cancer-related outpatient visit.	Numerator: -	Denominator: -	Exclusion: -	Performance standard: -	Included	N/A	07-DOMAIN 7 OPTIMAL TREATMENT OF SYMPTOMS AND PROVIDING COMFORT	Process
132	NEW Lorenz, 2009	Pain: IF a patient has advanced cancer and receives radiation treatment for painful bone metastases THEN s/he should be offered single-fraction radiation OR there should be documentation of a contraindication to single-fraction treatment.	Numerator: -	Denominator: -	Exclusion: -	Performance standard: -	Excluded	Procedural Relevance	N/A	N/A
133	NEW Lorenz, 2009	Pain: IF a cancer patient is treated for spinal cord compression THEN there should be follow-up of neurologic symptoms and signs within 1 week after treatment is completed.	Numerator: -	Denominator: -	Exclusion: -	Performance standard: -	Excluded	Procedural Relevance	N/A	N/A
134	NEW Claessen, 2011	Management of pain and other physical symptoms: Percentage of patients with moderate to severe pain<br>Pain is a common symptom in the palliative phase. The quality of both pharmacological and non-pharmacological interventions influences the severity of pain.	Numerator: The number of patients with a pain score of 4 or above on the NRS (average over 3 days)		Exclusion: Comatose and deeply sedated patients	Performance standard: -	Excluded	Scale specific	N/A	N/A

135	NEW Eagar, 2010	Change in pain<br>Change in pain is calculated by the difference in pain score from the beginning of a phase to the end of phase and is calculated using both PSS pain and SAS pain measures.	Percentage of patients with absent or mild pain at the beginning of their phase of palliative care have absent or mild pain at the end of the phase<br><br>Percentage of patients with moderate or severe pain at the beginning of their phase of palliative care have absent or mild pain at the end of the phase			Performance standard: 90%<br><br>Performance standard: 60%	Excluded	Scale specific	N/A	N/A
136	Mularski, 2006	Documentation of opioids, benzodiazepines, or similar agents prescribed to manage distress or dyspnea for non-comatose patients undergoing terminal withdrawal of mechanical ventilation.	Numerator: Total number of non-comatose patients for whom mechanical ventilation is withdrawn in anticipation of death who have an order writer for opiates or benzodiazepines as scheduled or as needed	Denominator: Total number of non-comatose patients for whom mechanical ventilation is withdrawn in anticipation of death	Exclusion: Comatose patients (e.g. Glasgow Coma Score of 2 or 3)	Performance standard: -	Excluded	Procedural Relevance	N/A	N/A
137	Keay, 1994	Dyspnea, if present, is addressed and attempts are made to minimize dyspnea.	Numerator: -	Denominator: -	Exclusion: Physicians not made aware of dyspnea	Performance standard: 100%	Included	N/A	07-DOMAIN 7 OPTIMAL TREATMENT OF SYMPTOMS AND PROVIDING COMFORT	Process
138	Peruselli, 1997	Palliative care services must meet the physical, psychological, social and spiritual needs of patients.	Numerator: Number of patients with global scores for breathing difficulties (TIQ scale) dropped after 8 days of care (if initial score on the same scale > 25)	Denominator: Total patients x 100	Exclusion: -	Performance standard: 75%	Excluded	Scale specific	N/A	N/A
139	Peruselli, 1997	Palliative care services must meet the physical, psychological, social and spiritual needs of patients.	Numerator: Number of patients with global scores for breathing difficulties (TIQ scale) not increased over initial score during final week of care (if initial score on the same scale > 25)	Denominator: Total patients x 100	Exclusion: -	Performance standard: 75%	Excluded	Scale specific	N/A	N/A
140	National Hospice and Palliative Care Organization (NHPCO), 2006 Brown University, 2006	Family evaluation of hospice care: Symptom management Questions:<br>B5: While under the care of hospice, did the patient have trouble breathing?<br>B6: How much help in dealing with his/her breathing did the patient	Numerator: Those who received too much or too little help concerning shortness of breath	Denominator: Those who experience shortness of breath	Exclusion: Patients who are not enrolled in a hospice program or have disenrolled from a hospice program. Live discharged are	Performance standard: -	Excluded	Relevance to long-term care settings	N/A	N/A

		receive while under the care of hospice?			excluded<br><br>Exclusion: -					
141	Twaddle, 2007	Dyspnea assessment<br>Percentage of all patients with documentation of dyspnea assessment within 48 hours of admission.	Numerator: -	Denominator: -	Exclusion: -	Performance standard: 90%	Excluded	Relevance to long-term care settings	N/A	N/A
142	Twaddle, 2007	Reduction or relief of dyspnea<br>Percentage of patients with dyspnea relieved or reduced (i.e. score of 3 or less on a 10-point scale) within 48 hours of admission.	Numerator: -	Denominator: Patients reporting dyspnea	Exclusion: -	Performance standard: 90%	Excluded	Relevance to long-term care settings	N/A	N/A
143	Lorenz, 2007	Mechanical ventilator withdrawal<br>IF a non-comatose VE is not expected to survive, and a mechanical ventilator is withdrawn or withheld, THEN the chart should document whether the patient has dyspnea, and the patient should receive (or have orders available for) an opiate, benzodiazepine, or barbiturate infusion, BECAUSE dyspnea can be controlled in the setting of comfort care when mechanical ventilation is withdrawn or withheld.	Numerator: -	Denominator: -	Exclusion: -	Performance standard: -	Excluded	Procedural Relevance	N/A	N/A
144	Lorenz, 2007<br>NEW Adapted by van der Ploeg, 2008<br>(see indicator 145)	Management of emergent dyspnea<br>IF a VE who has dyspnea in the last 7 days of life died an expected death, THEN the chart should document dyspnea care and follow-up, BECAUSE dyspnea can be effectively treated with oxygen and pharmacological agents.	Numerator: -	Denominator: -	Exclusion: -	Performance standard: -	Included	N/A	07-DOMAIN 7 OPTIMAL TREATMENT OF SYMPTOMS AND PROVIDING COMFORT	Process
145	NEW van der Ploeg, 2008<br>(Based on Lorenz, 2007, see indicator 144)	IF a vulnerable elder who had dyspnea in the last 7 days of life died an expected death, THEN the general practitioners record should document a dyspnea policy (including interventions).	Numerator: -	Denominator: -	Exclusion: -	Performance standard: -	Included	N/A	07-DOMAIN 7 OPTIMAL TREATMENT OF SYMPTOMS AND PROVIDING COMFORT	Process

146	Lorenz, 2007	Dyspnea assessment IF a VE is diagnosed with lung cancer, or cancer metastatic to lung, NYHA Class III to IV CHF, or oxygen-dependent pulmonary disease, THEN a **self-reported assessment** of dyspnea should be documented in the outpatient chart, BECAUSE dyspnea is common in these conditions, and there are effective treatments for addressing dyspnea.	Numerator: -	Denominator: -	Exclusion: -	Performance standard: -	Excluded	Procedural Relevance	N/A	N/A
147	Lorenz, 2007 NEW van der Ploeg, 2008	Treatment of dyspnea IF a VE with metastatic cancer or oxygen-dependent pulmonary disease has dyspnea refractory to non-opiate medications, THEN opiate medications should be offered, BECAUSE opiates effectively treat dyspnea from advanced cancer and chronic obstructive pulmonary disease.	Numerator: -	Denominator: -	Exclusion: -	Performance standard: -	Included	N/A	07-DOMAIN 7 OPTIMAL TREATMENT OF SYMPTOMS AND PROVIDING COMFORT	Process
148	Lorenz, 2007	Management of emergent dyspnea IF a VE is in hospice or has a preference for no hospitalization and is living with oxygen-dependent pulmonary disease, lung cancer, or NYHA Class III to IV CHF, THEN the medical record should document a plan for management of worsening or emergent dyspnea, BECAUSE increasing symptom burden is common with the progression of these illnesses and significantly impairs patient quality of life.	Numerator: -	Denominator: -	Exclusion: -	Performance standard: -	Included	N/A	07-DOMAIN 7 OPTIMAL TREATMENT OF SYMPTOMS AND PROVIDING COMFORT	Process
149	NEW Miyashita, 2008 Sato, 2008	Symptom control: Presence or absence of dyspnea	Numerator: Number of patients for whom this indicator was documented on admission or within the last 2 weeks of the hospitalization	Denominator: All patients who died (retrospectively identified)	Exclusion: -	Performance standard: -	Excluded	Relevance to long-term care settings	N/A	N/A
150	NEW Miyashita, 2008 Sato, 2008	Symptom control: Physician's prescription order for dyspnea	Numerator: Number of patients for whom this indicator was documented on admission or within the last 2 weeks of the hospitalization	Denominator: All patients who died (retrospectively identified)	Exclusion: -	Performance standard: -	Excluded	Relevance to long-term care settings	N/A	N/A

151	NEW Schenck, 2010	Physical Aspects of Care: Percent of patients who were screened for shortness of breath during the admission visit.	Numerator: Number of patients with dyspnea time=0 (Dyspnea time = dyspnea screening date –date of admission)	Denominator: # of patients	Exclusion: -	Performance standard: -	Excluded	Relevance to long-term care settings	N/A	N/A
152	NEW Schenck, 2010	Physical Aspects of Care: For patients who screened positive for dyspnea, the percent who received treatment within 1 day of screening.	Numerator: Number of patients with (0<=Dyspnea Treatment Time<=1 and dyspnea treatment="Y") Dyspnea Treatment time = date of treatment – dyspnea assessment date	Denominator: # patients with dyspnea	Exclusion: -	Performance standard: -	Included	N/A	07-DOMAIN 7 OPTIMAL TREATMENT OF SYMPTOMS AND PROVIDING COMFORT	Process
153	NEW Schenck, 2010	Physical Aspects of Care: For patients who screened positive for dyspnea, the percent of patients who improved within 1 day of screening.	Numerator: Number of patients with (0<=Improvement Time<=1 and Improvement=1) (Improvement Time = Second Dyspnea assessment date-Dyspnea assessment date)	Denominator: # patients with dyspnea	Exclusion: -	Performance standard: -	Included	N/A	07-DOMAIN 7 OPTIMAL TREATMENT OF SYMPTOMS AND PROVIDING COMFORT	Outcome
154	NEW Schenck, 2010	Physical Aspects of Care: For patients with moderate or severe shortness of breath, the percent with treatment or satisfied within 4 hours.	Numerator: Number of patients with moderate or severe shortness of breath with treatment or satisfied within 4 hours = "Y"	Denominator: Total number patients with moderate or severe dyspnea	Exclusion: -	Performance standard: -	Included	N/A	07-DOMAIN 7 OPTIMAL TREATMENT OF SYMPTOMS AND PROVIDING COMFORT	Process
155	NEW Lorenz, 2009	Dyspnea IF a patient with cancer reports new or worsening dyspnea, THEN there should be documentation of cause or of investigation of at least 1 of the following: hypoxia, anemia, bronchospasm or chronic obstructive pulmonary disease, pleural effusion, tumor obstruction of bronchi or the trachea, pneumonia, or pulmonary embolism.	Numerator: -	Denominator: -	Exclusion: -	Performance standard: -	Excluded	Procedural Relevance	N/A	N/A
156	NEW Lorenz, 2009	Dyspnea IF an outpatient with primary lung cancer or advanced cancer reports new or worsening dyspnea, THEN s/he should be offered symptomatic management or treatment directed at an underlying cause within 1 month.	Numerator: -	Denominator: -	Exclusion: -	Performance standard: -	Excluded	Procedural Relevance	N/A	N/A

157	NEW Lorenz, 2009	Dyspnea IF an inpatient with primary lung cancer or advanced cancer has dyspnea on admission, THEN s/he should be offered symptomatic management or treatment directed at an underlying cause within 24 hours.	Numerator: -	Denominator: -	Exclusion: -	Performance standard: -	Excluded	Procedural Relevance	N/A	N/A
158	NEW Lorenz, 2009	Dyspnea IF a patient with cancer in the hospital is treated for dyspnea, THEN there should be an assessment within 24 hours that the treatment was effective in relieving dyspnea OR that a change in treatment for dyspnea was made.	Numerator: -	Denominator: -	Exclusion: -	Performance standard: -	Included	N/A	07-DOMAIN 7 OPTIMAL TREATMENT OF SYMPTOMS AND PROVIDING COMFORT	Process
159	NEW Lorenz, 2009	Dyspnea IF a cancer patient has dyspnea and a malignant pleural effusion, THEN s/he should be offered thoracentesis within 1 month of the initial diagnosis of the effusion OR other treatment (e.g., diuresis) should result in a reduction in the effusion or symptomatic dyspnea.	Numerator: -	Denominator: -	Exclusion: -	Performance standard: -	Excluded	Procedural Relevance	N/A	N/A
160	NEW Lorenz, 2009	Dyspnea IF a cancer patient with a malignant pleural effusion undergoes thoracentesis, THEN there should be a repeat assessment of dyspnea within 1 week.	Numerator: -	Denominator: -	Exclusion: -	Performance standard: -	Excluded	Procedural Relevance	N/A	N/A
161	NEW Claessen, 2011	Management of pain and other physical symptoms: Percentage of patients with shortness of breath Shortness of breath often occurs in the palliative phase and can to a certain extent be influenced by pharmacological interventions (e.g. antibiotics, anticoagulants) and non-pharmacological interventions (e.g. oxygen administration, suction).	Numerator: The number of patients with a shortness of breath score of 4 or above on the NRS (average over 3 days)		Exclusion: Comatose and deeply sedated patients	Performance standard: -	Excluded	Scale specific	N/A	N/A
162	Peruselli, 1997	Palliative care services must meet the physical, psychological, social and spiritual needs of patients.	Numerator: Number of patients with global scores for constipation (TIQ scale) dropped after 8 days of care (if initial score on the same scale > 25)	Denominator: Total patients x 100	Exclusion: -	Performance standard: 75%	Excluded	Scale specific	N/A	N/A

163	Peruselli, 1997	Palliative care services must meet the physical, psychological, social and spiritual needs of patients.	Numerator: Number of patients with global scores for constipation (TIQ scale) not increased over initial score during final week of care (if initial score on the same scale > 25)	Denominator: Total patients x 100	Exclusion: -	Performance standard: 75%	Excluded	Scale specific	N/A	N/A
164	Peruselli, 1997	Palliative care services must meet the physical, psychological, social and spiritual needs of patients.	Numerator: Number of patients with global scores for gastrointestinal symptoms (TIQ scale) dropped after 8 days of care (if initial score on the same scale > 25)	Denominator: Total patients x 100	Exclusion: -	Performance standard: 75%	Excluded	Scale specific	N/A	N/A
165	Peruselli, 1997	Palliative care services must meet the physical, psychological, social and spiritual needs of patients.	Numerator: Number of patients with global scores for gastrointestinal symptoms (TIQ scale) not increased over initial score during final week of care (if initial score on the same scale > 25)	Denominator: Total patients x 100	Exclusion: -	Performance standard: 75%	Excluded	Scale specific	N/A	N/A
166	Twaddle, 2007	Bowel regimen ordered in conjunction with opioid administration<br>Percentage of patients receiving opioids who had an order for a bowel regimen written within 24 hours of order for the opioid.	Numerator: -	Denominator: -	Exclusion: -	Performance standard: 90%	Excluded	Relevance to long-term care settings	N/A	N/A
167	NEW Grunfeld, 2008	Potent antiemetics for emetogenic chemotherapy<br>Potent anti-emetic therapy for highly emetogenic chemotherapy treatments greatly controls nausea and vomiting. A high proportion may indicate good quality care.	Numerator: Number of cases 65 years of age and older who had a prescription for a potent antiemetic	Denominator: All cases 65 years of age and older	Exclusion: -	Performance standard:-	Excluded	Procedural Relevance	N/A	N/A
168	NEW Schenck, 2010	Physical Aspects of Care: For patients who screened positive for nausea, the percent who received treatment within 1 day of screening.	Numerator: Number of patients with (0<=Nausea Treatment Time<=1 and nausea treatment="Y") (Nausea Treatment time = date of treatment – nausea assessment date)	Denominator: Number of patients with nausea	Exclusion: -	Performance standard: -	Excluded	Procedural Relevance	N/A	N/A
169	NEW Schenck, 2010	Physical Aspects of Care: Percent of patients with bowel function assessed at least weekly.	Numerator: Number of patients with bowel function assessed weekly = "Y"	Denominator: Total # patients	Exclusion: -	Performance standard: -	Included	N/A	07-DOMAIN 7 OPTIMAL TREATMENT OF SYMPTOMS AND PROVIDING COMFORT	Process

170	NEW Schenck, 2010	Physical Aspects of Care: For patients who screened positive for constipation, the percent who receive treatment within 1 day of screening.	Numerator: Number of patients with (0<=Constipation Time<=1 and constipation treatment="Y")	Denominator: # patients with constipation (Constipation Treatment time = date of treatment – constipation assessment date)	Exclusion: -	Performance standard: -	Included	N/A	07-DOMAIN 7 OPTIMAL TREATMENT OF SYMPTOMS AND PROVIDING COMFORT	Process
171	NEW Schenck, 2010	Physical Aspects of Care: Percent of residents on opioids for whom a bowel regimen is established.	Numerator: Number of patients with opioids="Y" and bowel regimen="Y"	Denominator: # patients on opioids	Exclusion: -	Performance standard: -	Included	N/A	07-DOMAIN 7 OPTIMAL TREATMENT OF SYMPTOMS AND PROVIDING COMFORT	Process
172	NEW Schenck, 2010	Physical Aspects of Care: Percent of residents on opioids who have a bowel regimen within 1 day of opioid initiation.	Numerator: Number of patients with (0<=Time<=1 and opioids="Y" and bowel regimen="Y")	Denominator: # patients on opioids (Time = bowel regimen date – pain treatment date)	Exclusion: -	Performance standard: -	Included	N/A	07-DOMAIN 7 OPTIMAL TREATMENT OF SYMPTOMS AND PROVIDING COMFORT	Process
173	NEW Lorenz, 2009	Pain: IF a patient with cancer pain is started on chronic opioid treatment THEN s/he should be offered either a prescription or non-prescription bowel regimen within 24 hours OR there should be documented contraindication to a bowel regimen.	Numerator: -	Denominator: -	Exclusion: -	Performance standard: -	Excluded	Procedural Relevance	N/A	N/A
174	NEW Lorenz, 2009	Nausea and vomiting IF a patient with cancer undergoing moderately or highly emetic chemotherapy or with advanced cancer affecting the gastrointestinal tract or abdomen is seen for a visit in a cancer-related outpatient setting, THEN the presence or absence of nausea or vomiting should be assessed at every visit.	Numerator: -	Denominator: -	Exclusion: -	Performance standard: -	Excluded	Procedural Relevance	N/A	N/A
175	NEW Lorenz, 2009	Nausea and vomiting IF a patient with advanced cancer affecting the gastrointestinal tract or abdomen is admitted to a hospital, THEN the presence or absence of nausea or vomiting should be assessed within 24 hours.	Numerator: -	Denominator: -	Exclusion: -	Performance standard: -	Excluded	Relevance to long-term care settings	N/A	N/A
176	NEW Lorenz, 2009	Nausea and vomiting IF a patient with cancer is undergoing chemotherapy treatment with a high acute emetic risk, THEN a 3-drug regimen including single doses of a 5-HT3 receptor antagonist, dexamethasone, and selective neurokinin-1 receptor blocker should be given immediately before chemotherapy.	Numerator: -	Denominator: -	Exclusion: -	Performance standard: -	Excluded	Procedural Relevance	N/A	N/A

177	NEW Lorenz, 2009	Nausea and vomiting IF a patient with cancer is undergoing chemotherapy treatment with a moderate acute emetic risk, THEN a 2-drug regimen including a 5-HT3 receptor antagonist and dexamethasone should be given immediately before chemotherapy.	Numerator: -	Denominator: -	Exclusion: -	Performance standard: -	Excluded	Procedural Relevance	N/A	N/A
178	NEW Lorenz, 2009	Nausea and vomiting IF a patient with cancer reports nausea or vomiting on admission to the hospital, THEN within 24 hours potential underlying causes should be assessed.	Numerator: -	Denominator: -	Exclusion: -	Performance standard: -	Excluded	Relevance to long-term care settings	N/A	N/A
179	NEW Lorenz, 2009	Nausea and vomiting IF an inpatient with cancer has nausea or vomiting, THEN within 24 hours of the initial report of nausea and vomiting, the patient should be offered a change in therapy.	Numerator: -	Denominator: -	Exclusion: -	Performance standard: -	Excluded	Procedural Relevance	N/A	N/A
180	NEW Lorenz, 2009	Nausea and vomiting IF an outpatient with cancer not receiving chemotherapy or radiation is treated for nausea or vomiting with an antiemetic medication, THEN the effectiveness of treatment should be evaluated before or on the next visit to the same outpatient site.	Numerator: -	Denominator: -	Exclusion: -	Performance standard: -	Excluded	Procedural Relevance	N/A	N/A
181	NEW Lorenz, 2009	Anorexia/weight loss IF a patient presents for an initial visit for cancer affecting the oropharynx or gastrointestinal tract or advanced cancer at a cancer-related outpatient site, THEN there should be an assessment for the presence or absence of anorexia or dysphagia.	Numerator: -	Denominator: -	Exclusion: -	Performance standard: -	Excluded	Procedural Relevance	N/A	N/A
182	NEW Lorenz, 2009	Anorexia/weight loss IF a cancer patient is treated with an appetite stimulant for anorexia, THEN there should be an assessment before or on the next visit to the same outpatient site of whether there was an improvement in anorexia.	Numerator: -	Denominator: -	Exclusion: -	Performance standard: -	Excluded	Procedural Relevance	N/A	N/A
183	NEW Lorenz, 2009	Anorexia/weight loss IF a cancer patient is treated with enteral or parenteral nutrition, THEN there should be an assessment before starting nutrition that there was difficulty maintaining nutrition due to significant gastrointestinal issues and that life expectancy was at least 1 month.	Numerator: -	Denominator: -	Exclusion: -	Performance standard: -	Excluded	Procedural Relevance	N/A	N/A

184	NEW Claessen, 2011	Management of pain and other physical symptoms: Percentage of patients with constipation Constipation is a frequent and burdensome problem in the palliative phase and can be influenced particularly by pharmacological interventions (e.g. laxantia).	Numerator: The number of patients with a constipation score of 4 or above on the NRS (average over 3 days)		Exclusion: Comatose and deeply sedated patients	Performance standard: -	Excluded	Scale specific	N/A	N/A
185	Peruselli, 1997	Palliative care services must meet the physical, psychological, social and spiritual needs of patients.	Numerator: Number of patients with global scores for fatigue (TIQ scale) dropped after 8 days of care (if initial score on the same scale > 25)	Denominator: Total patients x 100	Exclusion: -	Performance standard: 75%	Excluded	Scale specific	N/A	N/A
186	Peruselli, 1997	Palliative care services must meet the physical, psychological, social and spiritual needs of patients.	Numerator: Number of patients with global scores for fatigue (TIQ scale) not increased over initial score during final week of care (if initial score on the same scale > 25)	Denominator: Total patients x 100	Exclusion: -	Performance standard: 75%	Excluded	Scale specific	N/A	N/A
187	NEW Lorenz, 2009	Fatigue/anemia IF a cancer patient is seen for an initial visit or any visit while undergoing chemotherapy at a cancer-related outpatient site, THEN there should be an assessment of the presence or absence of fatigue.	Numerator: -	Denominator: -	Exclusion: -	Performance standard: -	Excluded	Procedural Relevance	N/A	N/A
188	NEW Lorenz, 2009	Fatigue/anemia<br>IF a known cancer patient is newly diagnosed with advanced cancer, THEN there should be an assessment of the presence or absence of fatigue	Numerator: -	Denominator: -	Exclusion: -	Performance standard: -	Excluded	Procedural Relevance	N/A	N/A
189	NEW Lorenz, 2009	Fatigue/anemia IF a patient with cancer is found to have anemia with a hemoglobin <10 g/dL, THEN the presence and severity of anemia- related symptoms (e.g., fatigue, dyspnea, and lightheadedness) should be evaluated.	Numerator: -	Denominator: -	Exclusion: -	Performance standard: -	Excluded	Procedural Relevance	N/A	N/A
190	NEW Lorenz, 2009	Fatigue/anemia IF a patient with cancer is found to have severe, symptomatic anemia (hemoglobin <8 g/dL), THEN transfusion with packed red cells should be offered to the patient within 24 hours.	Numerator: -	Denominator: -	Exclusion: -	Performance standard: -	Excluded	Procedural Relevance	N/A	N/A

191	NEW Claessen, 2011	Management of pain and other physical symptoms: Percentage of patients with fatigue<br>Fatigue is one of the most common symptoms in cancer patients. Both pharmacological and non-pharmacological interventions (e.g. relaxation therapy) are appropriate treatment options.	Numerator: The number of patients with a fatigue score of 4 or above on the NRS (average over 3 days)		Exclusion: Comatose and deeply sedated patients	Performance standard: -	Excluded	Scale specific	N/A	N/A
192	Yabroff, 2004	Percentage of patients within and among health facilities or systems with evidence that symptom relief was achieved and unmet needs were met with appropriate response or resolved.	Numerator: -	Denominator: -	Exclusion: -	Performance standard: -	Included	N/A	07-DOMAIN 7 OPTIMAL TREATMENT OF SYMPTOMS AND PROVIDING COMFORT	Outcome
193	Peruselli, 1997	Palliative care services must meet the physical, psychological, social and spiritual needs of patients.	Numerator: Number of patients with score for symptom control other than pain (STAS item) dropped after 8 days of care (if initial score >0)	Denominator: Total patients x 100	Exclusion: -	Performance standard: 75%	Excluded	Scale specific	N/A	N/A
194	Peruselli, 1997	Palliative care services must meet the physical, psychological, social and spiritual needs of patients.	Numerator: Number of patients with score symptom control other than pain (STAS item) of 0-1 during final week of life	Denominator: Total patients x 100	Exclusion: -	Performance standard: 75%	Excluded	Scale specific	N/A	N/A
195	Peruselli, 1997	Palliative care services must meet the physical, psychological, social and spiritual needs of patients.	Numerator: Number of patients with global scores for physical symptoms (= comprising TIQ scales: fatigue, gastrointestinal symptoms and global health status) dropped after 8 days of care (if initial score on the same scale > 25)	Denominator: Total patients x 100	Exclusion: -	Performance standard: 75%	Excluded	Scale specific	N/A	N/A
196	Peruselli, 1997	Palliative care services must meet the physical, psychological, social and spiritual needs of patients.	Numerator: Number of patients with global scores for physical symptoms (= comprising TIQ scales: fatigue, gastrointestinal symptoms and global health status) not increased over initial score during final week of care (if initial score on the same scale > 25)	Denominator: Total patients x 100	Exclusion: -	Performance standard: 75%	Excluded	Scale specific	N/A	N/A

197	Peruselli, 1997	Palliative care services must meet the physical, psychological, social and spiritual needs of patients.	Numerator: Number of patients with global scores for global health status (TIQ scale) dropped after 8 days of care (if initial score on the same scale > 25)	Denominator: Total patients x 100	Exclusion: -	Performance standard: 75%	Excluded	Scale specific	N/A	N/A
198	Peruselli, 1997	Palliative care services must meet the physical, psychological, social and spiritual needs of patients.	Numerator: Number of patients with global scores for global health status (TIQ scale) not increased over initial score during final week of care (if initial score on the same scale > 25)	Denominator: Total patients x 100	Exclusion: -	Performance standard: 75%	Excluded	Scale specific	N/A	N/A
199	NEW Sato, 2008	Symptom control: Effect of rescue medication for any physical distress	Numerator: Number of patients for whom this indicator was documented on admission or within the last 2 weeks of the hospitalization	Denominator: All patients who died (retrospectively identified)	Exclusion: -	Performance standard: -	Excluded	Procedural Relevance	N/A	N/A
200	NEW van der Ploeg, 2008<br>This indicator suits domain 2,3,4 and 8.	IF a vulnerable elder dies with a progressive incurable disease (for example metastatic cancer, or dementia) THEN there should be evidence within 6 months prior to death that they received a comprehensive assessment including:<br>· Pain;<br>· Anxiety, depression;<br>· Vomiting and dyspnea;<br>· Spiritual and existential concerns;<br>· Caregiver burdens/need for practical assistance;<br>· Wishes concerning medical treatment and care at the end of life;<br>A discussion about and if possible the determination of a surrogate decision maker.	Numerator: -	Denominator: -	Exclusion: -	Performance standard: -	Included	N/A	07-DOMAIN 7 OPTIMAL TREATMENT OF SYMPTOMS AND PROVIDING COMFORT	Process
201	NEW Miyashita, 2008<br>Sato, 2008	Symptom control: Observation and care of mouth	Numerator: Number of patients for whom this indicator was documented on admission or within the last 2 weeks of the hospitalization	Denominator: All patients who died (retrospectively identified)	Exclusion: -	Performance standard: -	Included	N/A	07-DOMAIN 7 OPTIMAL TREATMENT OF SYMPTOMS AND PROVIDING COMFORT	Process

202	NEW Lorenz, 2009	Pain: IF a cancer patient has new neurologic symptoms or findings on physical examination consistent with spinal cord compression THEN s/he should be treated with steroids as soon as possible, but within 24 hours OR a contraindication to steroids should be documented.	Numerator: -	Denominator: -	Exclusion: -	Performance standard: -	Excluded	Procedural Relevance	N/A	N/A
203	NEW Lorenz, 2009	Pain: IF a cancer patient has new neurologic symptoms or findings on physical examination consistent with spinal cord compression THEN a whole-spine MRI scan or myelography should be performed as soon as possible, but within 24 hours OR there should be documentation of why an MRI scan was not appropriate.	Numerator: -	Denominator: -	Exclusion: -	Performance standard: -	Excluded	Procedural Relevance	N/A	N/A
204	NEW Lorenz, 2009	Pain: IF a cancer patient has confirmation of spinal cord compression on radiologic examination, THEN radiotherapy or surgical decompression should be initiated within 24 hours OR a contraindication for such therapy should be documented.	Numerator: -	Denominator: -	Exclusion: -	Performance standard: -	Excluded	Procedural Relevance	N/A	N/A
205	NEW Lorenz, 2009	Treatment-associated toxicities - Diarrhea IF a patient with cancer is undergoing chemotherapy and has diarrhea THEN to classify the diarrhea as complicated or uncomplicated all of the following should be assessed: history of onset and duration; number of stools and stool composition; and at least 1 of the associated symptoms (fever, dizziness, abdominal pain/cramping, nausea/vomiting, decreased performance status, sepsis, fever, bleeding, or dehydration).	Numerator: -	Denominator: -	Exclusion: -	Performance standard: -	Excluded	Procedural Relevance	N/A	N/A
206	NEW Lorenz, 2009	Treatment-associated toxicities - Diarrhea IF a patient with cancer is undergoing chemotherapy with a high risk (>10%) of chemotherapy-induced diarrhea THEN an antidiarrheal agent should be prescribed on or before treatment is initiated.	Numerator: -	Denominator: -	Exclusion: -	Performance standard: -	Excluded	Procedural Relevance	N/A	N/A
207	NEW Lorenz, 2009	Skin rash IF a patient with cancer who is being treated with agents that block epidermal growth factor receptors, THEN the presence and severity of skin rash should be	Numerator: -	Denominator: -	Exclusion: -	Performance standard: -	Excluded	Procedural Relevance	N/A	N/A

		evaluated within 1 month after starting the treatment and at each visit.							
208	Peruselli, 1997	Palliative care services must meet the physical, psychological, social and spiritual needs of patients.	Numerator: Number of patients with global scores for functional impairment (TIQ scale) dropped after 8 days of care (if initial score on the same scale > 25)	Denominator: Total patients x 100	Exclusion: -	Performance standard: 75%	Excluded	Scale specific	N/A	N/A
209	Peruselli, 1997	Palliative care services must meet the physical, psychological, social and spiritual needs of patients.	Numerator: Number of patients with global scores for functional impairment (TIQ scale) not increased over initial score during final week of care (if initial score on the same scale > 25)	Denominator: Total patients x 100	Exclusion: -	Performance standard: 75%	Excluded	Scale specific	N/A	N/A
210	Saliba, 2004	IF a nursing home resident was conscious during any of the last 7 days of life and died an expected death THEN there should be medical record documentation about emotional distress (presence, absence, or inability to assess) in the last 7 days of life.	Numerator:-	Denominator: -	Exclusion: -	Performance standard: -	Included	N/A	07-DOMAIN 7 OPTIMAL TREATMENT OF SYMPTOMS AND PROVIDING COMFORT	Process
211	Peruselli, 1997	Palliative care services must meet the physical, psychological, social and spiritual needs of patients.	Numerator: Number of patients with score for patient anxiety (STAS item) dropped after 8 days of care (if initial score >0)	Denominator: Total patients x 100	Exclusion: -	Performance standard: 75%	Excluded	Scale specific	N/A	N/A
212	Peruselli, 1997	Palliative care services must meet the physical, psychological, social and spiritual needs of patients.	Numerator: Number of patients with score for patient anxiety (STAS item) of 0-1 during final week of life		Exclusion: -	Performance standard: 75%	Excluded	Scale specific	N/A	N/A
213	Peruselli, 1997	Palliative care services must meet the physical, psychological, social and spiritual needs of patients.	Numerator: Number of patients with score for family anxiety (STAS item) dropped after 8 days of care (if initial score >0)	Denominator: Total patients x 100	Exclusion: -	Performance standard: 75%	Excluded	Scale specific	N/A	N/A
214	Peruselli, 1997	Palliative care services must meet the physical, psychological, social and spiritual needs of patients.	Numerator: Number of patients with score for family anxiety (STAS item) of 0-1 during final week of life		Exclusion: -	Performance standard: 75%	Excluded	Scale specific	N/A	N/A

215	National Hospice and Palliative Care Organization (NHPCO), 2006 Brown University, 2006	Family evaluation of hospice care: Symptom management Questions: B9: While under the care of hospice, did the patient have any feelings of anxiety or sadness? B10: How much help in dealing with these feelings did the patient receive?	Numerator: Those who received too much or too little help concerning anxiety/sadness	Denominator: Those who experience anxiety/sadness	Exclusion: Patients who are not enrolled in a hospice program or have disenrolled from a hospice program. Live discharged are excluded	Performance standard: -	Excluded	Relevance to long-term care settings	N/A	N/A
216	NEW Miyashita, 2008 Sato, 2008	Symptom control: Presence or absence of delirium or agitation.	Numerator: Number of patients for whom this indicator was documented on admission or within the last 2 weeks of the hospitalization	Denominator: All patients who died (retrospectively identified)	Exclusion: -	Performance standard: -	Included	N/A	07-DOMAIN 7 OPTIMAL TREATMENT OF SYMPTOMS AND PROVIDING COMFORT	Outcome
217	NEW Miyashita, 2008 Sato, 2008	Symptom control: Physician's prescription order for delirium or agitation.	Numerator: Number of patients for whom this indicator was documented on admission or within the last 2 weeks of the hospitalization	Denominator: All patients who died (retrospectively identified)	Exclusion: -	Performance standard: -	Included	N/A	07-DOMAIN 7 OPTIMAL TREATMENT OF SYMPTOMS AND PROVIDING COMFORT	Process
218	NEW Miyashita, 2008 Sato, 2008	Psychosocial and spiritual concerns: Degree and content of patient's anxiety.	Numerator: Number of patients for whom this indicator was documented on admission or within the last 2 weeks of the hospitalization	Denominator: All patients who died (retrospectively identified)	Exclusion: -	Performance standard: -	Excluded	Relevance to long-term care settings	N/A	N/A
219	NEW Schenck, 2010	Psychological Aspects of Care: For patients who screened positive for anxiety, the percent who receive treatment within two weeks of diagnosis.	Numerator: Number of patients with (0<=Time<=14 days and anxiety treatment="Y") (Anxiety treatment time = date of treatment – anxiety assessment date)	Denominator: # patients with anxiety screening=Y	Exclusion: -	Performance standard: -	Excluded	Procedural Relevance	N/A	N/A
220	NEW Lorenz, 2009	Delirium IF a hospitalized patient with cancer aged >65 years or with advanced cancer has delirium THEN there should be an assessment for the presence or absence of at least 1 of the following potential causes and their association with delirium: medication effects, central nervous system disease, infection, and metabolic processes.	Numerator: -	Denominator: -	Exclusion: -	Performance standard: -	Included	N/A	07-DOMAIN 7 OPTIMAL TREATMENT OF SYMPTOMS AND PROVIDING COMFORT	Process

221	NEW Claessen, 2011	Care for psychosocial well-being of patients: Percentage of patients with anxiety<br>Anxiety is a common symptom for many patients in the palliative phase, as a reaction to their illness and the prospect of approaching death. Anxiety can be influenced by pharmacological and non-pharmacological interventions (e.g. psychosocial support).	Numerator: The number of patients with an anxiety score of 4 or above on the NRS (average over 3 days)		Exclusion: Patients with moderate to (very) severe cognitive impairments, young children, psychiatric and/or confused patients, and comatose and deeply sedated patients	Performance standard: -	Excluded	Scale specific	N/A	N/A
222	NEW Schenck, 2010	Psychological Aspects of Care: For patients who screened positive for depression, the percent who received further assessment, counseling or medication treatment.	Numerator: Number of patients with depression further assessment="Y"	Denominator: # patients with depression screening=Yes	Exclusion: -	Performance standard: -	Excluded	Procedural Relevance	N/A	N/A
223	NEW Schenck, 2010	Psychological Aspects of Care: For patients diagnosed with depression, the percent who receive treatment within two weeks of diagnosis.	Numerator: Number of patients with (0<=Depression Treatment Time<=14 days and depression treatment="Y")	Denominator: # patients with depression diagnosis =Y (Depression treatment time = date of treatment – depression diagnosis date)	Exclusion: -	Performance standard: -	Excluded	Procedural Relevance	N/A	N/A
224	NEW Lorenz, 2009	Depression and psychosocial distress IF depression is diagnosed in a cancer patient, THEN a treatment plan for depression should be documented.	Numerator: -	Denominator: -	Exclusion: -	Performance standard: -	Excluded	Procedural Relevance	N/A	N/A
225	NEW Lorenz, 2009	Depression and psychosocial distress IF a patient with cancer is treated for depression, THEN response to therapy should be documented within 6 weeks.	Numerator: -	Denominator: -	Exclusion: -	Performance standard: -	Excluded	Procedural Relevance	N/A	N/A
226	NEW Claessen, 2011	Care for psychosocial well-being of patients: Percentage of patients who feel depressed<br>Depression is a common symptom in the palliative phase, and can be influenced most by non-pharmacological interventions (listening to them), and to a lesser extent by pharmacological interventions (e.g. antidepressants).	Numerator: The number of patients with a feeling depressed score of 4 or above on the NRS (average over 3 days).		Exclusion: Patients with moderate to (very) severe cognitive impairments, young children, psychiatric and/or confused patients, and comatose and deeply sedated patients	Performance standard: -	Excluded	Scale specific	N/A	N/A
227	Peruselli, 1997	Palliative care services must meet the physical, psychological, social and spiritual needs of patients.	Numerator: Number of patients with global scores for cognitive status (TIQ scale) dropped after 8 days of care (if initial score on the same scale > 25)	Denominator: Total patients x 100	Exclusion: -	Performance standard: 75%	Excluded	Scale specific	N/A	N/A

228	Peruselli, 1997	Palliative care services must meet the physical, psychological, social and spiritual needs of patients.	Numerator: Number of patients with global scores for cognitive status (TIQ scale) not increased over initial score during final week of care (if initial score on the same scale > 25)	Denominator: Total patients x 100	Exclusion: -	Performance standard: 75%	Excluded	Scale specific	N/A	N/A
229	National Hospice and Palliative Care Organization (NHPCO), 2006 Brown University, 2006	Attend to family needs (Family evaluation of hospice care) Questions: E2: Did you have as much contact about your religious or spiritual beliefs as you wanted? E3: How much emotional support did the hospice team provide to you prior to the patient's death? E4: How much emotional support did the hospice team provide to you after the patient's death?	Numerator: Sum of 1 response to survey instrument by family member of deceased patient	Denominator: Number of items (=3)	Exclusion: Patients who are not enrolled in a hospice program or have disenrolled from a hospice program. Live discharged are excluded	Performance standard: -	Excluded	Relevance to long-term care settings	N/A	N/A
230	Yabroff, 2004	Percentage of patients within and among health facilities or systems with evidence for ongoing quality of life assessment reflected in the treatment plan.	Numerator: -	Denominator: -	Exclusion: -	Performance standard: -	Included	N/A	03-DOMAIN 3 SETTING CARE GOALS AND ADVANCE PLANNING	Process
231	Peruselli, 1997	Palliative care services must meet the physical, psychological, social and spiritual needs of patients.	Numerator: Number of patients with global scores for emotional status (TIQ scale) dropped after 8 days of care (if initial score on the same scale > 25)		Exclusion: -	Performance standard: 75%	Excluded	Scale specific	N/A	N/A
232	Peruselli, 1997	Palliative care services must meet the physical, psychological, social and spiritual needs of patients.	Numerator: Number of patients with global scores for emotional status (TIQ scale) not increased over initial score during final week of care (if initial score on the same scale > 25)		Exclusion: -	Performance standard: 75%	Excluded	Scale specific	N/A	N/A
233	Peruselli, 1997	Palliative care services must meet the physical, psychological, social and spiritual needs of patients.	Numerator: Number of patients with global scores for therapy impact index (= comprising functional impairment, emotional status, cognitive status, social interaction TIQ scales) dropped after 8 days of care (if initial score on the same scale > 25)	Denominator: Total patients x 100	Exclusion: -	Performance standard: 75%	Excluded	Scale specific	N/A	N/A

234	Peruselli, 1997	Palliative care services must meet the physical, psychological, social and spiritual needs of patients.	Numerator: Number of patients with global scores for therapy impact index (= comprising functional impairment, emotional status, cognitive status, social interaction TIQ scales) not increased over initial score during final week of care (if initial score on the same scale > 25)	Denominator: Total patients x 100	Exclusion: -	Performance standard: 75%	Excluded	Scale specific	N/A	N/A
235	Peruselli, 1997	Palliative care services must meet the physical, psychological, social and spiritual needs of patients.	Numerator: Number of patients with score for communication between patient en family (STAS item) dropped after 8 days of care (if initial score >0)	Denominator: Total patients x 100	Exclusion: -	Performance standard: 75%	Excluded	Scale specific	N/A	N/A
236	Peruselli, 1997	Palliative care services must meet the physical, psychological, social and spiritual needs of patients.	Numerator: Number of patients with score for communication between patient en family (STAS item) of 0-1 during final week of life	Denominator: Total patients x 100	Exclusion: -	Performance standard: 75%	Excluded	Scale specific	N/A	N/A
237	Twaddle, 2007	Psychosocial assessment<br>All patients were expected to have a psychosocial assessment (i.e. a formal psychosocial assessment conducted by a psychologist, social worker, psychiatrist or other expert) completed within 1 year prior or 4 days after admission.	Numerator: -	Denominator: -	Exclusion: -	Performance standard: 90%	Excluded	Relevance to long-term care settings	N/A	N/A
238	NEW Miyashita, 2008	Psychosocial and spiritual concerns: Patient's preference of daily living.	Numerator: Number of patients for whom this indicator was documented on admission or within the last 2 weeks of the hospitalization	Denominator: All patients who died (retrospectively identified)	Exclusion: -	Performance standard: -	Included	N/A	08-DOMAIN 8 PSYCHOSOCIAL AND SPIRITUAL SUPPORT	Process
239	Keay, 1994	Psychological or social support is documented in the patient's medical record	Numerator: -	Denominator: -	Exclusion: Patient death within a few hours of being declared terminally ill; patient cognitively impaired and family or friends are not reasonably available	Performance standard: > 80%	Included	N/A	08-DOMAIN 8 PSYCHOSOCIAL AND SPIRITUAL SUPPORT	Process

240	Mularski, 2006	Documentation of offering of psychosocial support within the first 72 hours of admission to the ICU.	Numerator: Total number of patients in the ICU for > 72 hours with psychosocial support offered to the patient or family by any team member	Denominator: Total number of patients in the ICU for > 72 hours	Exclusion: Comatose patients (e.g. Glasgow Coma Score of 2 or 3) with no family member of friend identified	Performance standard: -	Excluded	Relevance to long-term care settings	N/A	N/A
241	Nelson, 2006	Social work support: percentage of patients with documentation that social work support was offered to the patient/family.	Numerator: Number of patients with documentation that social work support was offered to the patient/family	Denominator: Total number of patients with ICU length of stay > 3 days	Exclusion: -	Performance standard: -	Excluded	Relevance to long-term care settings	N/A	N/A
242	Lorenz, 2007 NEW van der Ploeg, 2008	Caregiver stress IF a VE is a caregiver for a spouse, significant other , or dependent who is terminally ill or has very limited function, THEN the VE should be assessed for caregiver financial, physical, and emotional stress, BECAUSE caregiver burden is substantial in these situations and associated with poor outcomes.	Numerator: -	Denominator: -	Exclusion: -	Performance standard: -	Included	N/A	09-DOMAIN 9 FAMILY CARE AND INVOLVEMENT	Process
243	NEW Miyashita, 2008	Family care: Care strategy for family	Numerator: Number of patients for whom this indicator was documented on admission or within the last 2 weeks of the hospitalization	Denominator: All patients who died (retrospectively identified)	Exclusion: -	Performance standard: -	Included	N/A	09-DOMAIN 9 FAMILY CARE AND INVOLVEMENT	Process
244	NEW Miyashita, 2008	Psychosocial and spiritual concerns: Coordination of social resources when patient had no family or friends.	Numerator: Number of patients for whom this indicator was documented on admission or within the last 2 weeks of the hospitalization	Denominator: All patients who died (retrospectively identified)	Exclusion: -	Performance standard: -	Excluded	Relevance to long-term care settings	N/A	N/A
245	NEW Quality Markers, 2009	QM for primary care: They have mechanisms in place to assess and document the needs of carers of those approaching the end of life (Royal College of General Practicioners' Supporting Carers).	Proportion of individuals whose carer is recorded	Numerator: - Denominator: -	Exclusion: -	Performance standard: -	Included	N/A	09-DOMAIN 9 FAMILY CARE AND INVOLVEMENT	Process
246	NEW Quality Markers, 2009	QM for primary care: They have mechanisms in place to assess and document the needs of carers of those approaching the end of life (Royal College of General Practicioners' Supporting Carers).	Proportion of carers who have been referred to a carer's assessment and whose needs have been recorded	Numerator: - Denominator: -	Exclusion: -	Performance standard: -	Included	N/A	09-DOMAIN 9 FAMILY CARE AND INVOLVEMENT	Process

247	NEW ELCQuA, 2011	The needs and preferences of carers are assessed and recorded.	Proportion of deaths where the patient/service user had a recorded carer<br><br>Numerator: Number of patients who died whose carer was recorded, including n/a.<br><br>Proportion of carers referred for a carer's assessment<br><br>Numerator: Number of carers who have been referred for a carer's assessment<br><br>Proportion of carers referred for assessment actually receiving an assessment<br><br>Numerator: Number of carers whose needs have been recorded	Denominator: Total deaths for same time period<br><br>Denominator: Total number of carers<br><br>Denominator: Number of carers referred for assessment	Exclusion: -<br><br>Exclusion: -<br><br>Exclusion: -	Performance standard: Carer's assessments undertaken for all appropriate cases<br><br>Performance standard: Carer's assessments undertaken for all appropriate cases<br><br>Performance standard: Carer's assessments undertaken for all appropriate cases	Included	N/A	09-DOMAIN 9 FAMILY CARE AND INVOLVEMENT	Process
248	Peruselli, 1997	Support provided to patient and family also includes home visits by voluntary workers.	Numerator: Number of patients who have had home visits by voluntary workers	Denominator: Population served x 100	Exclusion: -	Performance standard: 30%	Excluded	Relevance to UK care settings	N/A	N/A
249	Peruselli, 1997	Palliative care services must meet the physical, psychological, social and spiritual needs of patients.	Numerator: Number of patients with global scores for social interaction (TIQ scale) dropped after 8 days of care (if initial score on the same scale > 25)		Exclusion: -	Performance standard: 75%	Excluded	Scale specific	N/A	N/A
250	Peruselli, 1997	Palliative care services must meet the physical, psychological, social and spiritual needs of patients.	Numerator: Number of patients with global scores for social interaction (TIQ scale) not increased over initial score during final week of care (if initial score on the same scale > 25)	Denominator: Total patients x 100	Exclusion: -	Performance standard: 75%	Excluded	Scale specific	N/A	N/A

251	NEW Miyashita, 2008<br>Sato, 2008	Family care: Configuration of family relationships.	Numerator: Number of patients for whom this indicator was documented on admission or within the last 2 weeks of the hospitalization	Denominator: All patients who died (retrospectively identified)	Exclusion: -	Performance standard: -	Included	N/A	09-DOMAIN 9 FAMILY CARE AND INVOLVEMENT	Process
252	NEW Sato, 2008	Family care: Family's insight of disease.	Numerator: Number of patients for whom this indicator was documented on admission or within the last 2 weeks of the hospitalization	Denominator: All patients who died (retrospectively identified)	Exclusion: -	Performance standard: -	Included	N/A	09-DOMAIN 9 FAMILY CARE AND INVOLVEMENT	Process
253	NEW Sato, 2008	Family care: Discussion with family about goals of care.	Numerator: Number of patients for whom this indicator was documented on admission or within the last 2 weeks of the hospitalization	Denominator: All patients who died (retrospectively identified)	Exclusion: -	Performance standard: -	Included	N/A	02-DOMAIN 2 PERSON-CENTRED CARE, COMMUNICATION AND SHARED DECSION MAKING	Process
254	Nelson, 2006, Mularski, 2006	Spiritual support: percentage of patients with documentation that spiritual support was offered to the patient /family.	Numerator: Number of patients with documentation that spiritual support was offered to the patient/family	Denominator: Total number of patients with ICU length of stay > 3 days	Exclusion: patients with no family members visiting the patient during the ICU stay	Performance standard: -	Included	N/A	08-DOMAIN 8 PSYCHOSOCIAL AND SPIRITUAL SUPPORT	Process
255	NEW Miyashita, 2008<br>Sato, 2008	Psychosocial and spiritual concerns: Patient's religion.	Numerator: Number of patients for whom this indicator was documented on admission or within the last 2 weeks of the hospitalization	Denominator: All patients who died (retrospectively identified)	Exclusion: -	Performance standard: -	Included	N/A	08-DOMAIN 8 PSYCHOSOCIAL AND SPIRITUAL SUPPORT	Process
256	NEW Schenck, 2010	Spiritual Aspects of Care: Percent of patients with chart documentation of a discussion of spiritual concerns.	Numerator: Number of patients with spiritual discussion = "Y"	Denominator: Total number of patients	Exclusion: -	Performance standard: -	Included	N/A	08-DOMAIN 8 PSYCHOSOCIAL AND SPIRITUAL SUPPORT	Process
257	NEW Miyashita, 2008	Psychosocial and spiritual concerns: Emotional reaction to explanation of medical condition.	Numerator: Number of patients for whom this indicator was documented on admission or within the last 2 weeks of the hospitalization	Denominator: All patients who died (retrospectively identified)	Exclusion: -	Performance standard: -	Included	N/A	08-DOMAIN 8 PSYCHOSOCIAL AND SPIRITUAL SUPPORT	Process
258	NEW Claessen, 2011	Care for spiritual well-being of patients: Percentage of relatives who indicate that the patient died peacefully<br>If patients die peacefully, this can indicate that in this respect their spiritual needs were met.	Numerator: The number of relatives who indicate that their relative died peacefully	Denominator: The total number of relatives among whom this quality indicator was measured	Exclusion: -	Performance standard:	Included	N/A	08-DOMAIN 8 PSYCHOSOCIAL AND SPIRITUAL SUPPORT	Outcome

259	NEW Claessen, 2011	Care for spiritual well-being of patients: Percentage of relatives who indicate that the patient had accepted her/his approaching death.<br>If patients accept their approaching death, this can indicate that their spiritual needs were met.	Numerator: The number of relatives who indicate that their relative had accepted approaching death	Denominator: The total number of relatives among whom this quality indicator was measured	Exclusion: -	Performance standard:	Included	N/A	08-DOMAIN 8 PSYCHOSOCIAL AND SPIRITUAL SUPPORT	Outcome
260	NEW Miyashita, 2008	Family care: Family's preference of explanation of medical condition.	Numerator: Number of patients for whom this indicator was documented on admission or within the last 2 weeks of the hospitalization	Denominator: All patients who died (retrospectively identified)	Exclusion: -	Performance standard: -	Included	N/A	09-DOMAIN 9 FAMILY CARE AND INVOLVEMENT	Process
261	Earle, 2006	Proportion receiving chemotherapy in the last 14 days of life.	Numerator: Patients who died from cancer and received chemotherapy in the last 14 days of life	Denominator: Patients who died from cancer	Exclusion: -	Performance standard: < 10%	Excluded	Procedural Relevance	N/A	N/A
262	Earle, 2006	Proportion with more than one emergency room (ER) visit in the last 30 days of life.	Numerator: Patients who died from cancer and had > 1 ER visit in the last 30 days of life	Denominator: Patients who died from cancer	Exclusion: -	Performance standard: < 4%	Included	N/A	06-DOMAIN 6 AVOIDING OVERLY AGGRESSIVE, BURDENSOME OR FUTILE TREATMENT	Outcome
263	NEW Grunfeld, 2008	Frequency of ER visits<br>High number of emergency room visits near death may indicate poor quality care	Numerator: Number of cases with more than 1 ER visit in the last 30 days<br><br>Numerator: Number of ER visits in the last 30 days averaged across entire cohort<br><br>Numerator: Sum of ER visits across entire cohort	Denominator: entire cohort<br><br>Denominator: - entire cohort<br><br>Denominator: The sum of available days (i.e., days out of hospital) across entire cohort)	Exclusion: -<br><br>Exclusion: -<br><br>Exclusion: -	Performance standard: -<br><br>Performance standard: -<br><br>Performance standard: -	Included	N/A	06-DOMAIN 6 AVOIDING OVERLY AGGRESSIVE, BURDENSOME OR FUTILE TREATMENT	Outcome
264	Earle, 2006	Proportion with more than one hospitalization in the last 30 days of life.	Numerator: Patients who died from cancer and had > 1 hospitalization in the last 30 days of life	Denominator: Patients who died from cancer	Exclusion: -	Performance standard: < 4%	Included	N/A	06-DOMAIN 6 AVOIDING OVERLY AGGRESSIVE, BURDENSOME OR FUTILE TREATMENT	Outcome
265	Earle, 2006	Proportion admitted to the ICU in the last 30 days of life.	Numerator: Patients who died from cancer and were admitted to the ICU in the last 30 days of life	Denominator: Patients who died from cancer	Exclusion: -	Performance standard: < 4%	Excluded	Relevance to long-term care settings	N/A	N/A
266	NEW Grunfeld, 2008	ICU stays near the end of life<br>Hospital stays in the terminal period of cancer may indicate poor quality care.	Numerator: Number of cases with one or more ICU admissions in the last 30 days	Denominator: Entire cohort	Exclusion: -	Performance standard: -	Excluded	Relevance to long-term care settings	N/A	N/A

267	NEW Grunfeld, 2008	Enrollment in palliative care within 3 days of death<br>A high proportion may indicate poor quality care. Palliative care should be accessible to all patients and families with a cancer diagnosis, in a timely manner, throughout the entire duration of their disease.	Numerator: Number of cases enrolled in palliative care within three days prior to death	Denominator: All cases enrolled in palliative care	Exclusion: -	Performance standard: -	Included	N/A	05-DOMAIN 5 PROGNOSTICATIO N AND TIMELY RECOGNITION OF DYING	Outcome
268	NEW Miyashita, 2008	Decision making and preference of care: Time of patient's enrollment in palliative care program and documented medical history.	Numerator: Number of patients for whom this indicator was documented on admission or within the last 2 weeks of the hospitalization	Denominator: All patients who died (retrospectively identified)	Exclusion: -	Performance standard: -	Included	N/A	05-DOMAIN 5 PROGNOSTICATIO N AND TIMELY RECOGNITION OF DYING	Process
269	Mularski, 2006	Documentation of a protocol for provision of analgesia and sedation during terminal withdrawal of mechanical ventilation.	Numerator: Presence of a protocol that can be applied in settings of terminal withdrawal of mechanical ventilation	Denominator: ICU	Exclusion: -	Performance standard: -	Excluded	Procedural Relevance	N/A	N/A
270	Peruselli, 1997	Home palliative care services must ensure continuous care for the patient until the final moments of life, and for the family after the patient's death by helping to deal with the mourning process.	Numerator: Number of families who have received home visits on the day of patient's death	Denominator: Population served x 100	Exclusion: -	Performance standard: 90%	Included	N/A	09-DOMAIN 9 FAMILY CARE AND INVOLVEMENT	Outcome
271	Peruselli, 1997	Home palliative care services must ensure continuous care for the patient until the final moments of life, and for the family after the patient's death by helping to deal with the mourning process.	Numerator: Number of families who have received home visits in the days following patient's death to provide support during the mourning process	Denominator: Population served x 100	Exclusion: -	Performance standard: 90%	Included	N/A	09-DOMAIN 9 FAMILY CARE AND INVOLVEMENT	Outcome
272	NEW Grunfeld, 2008	Time and location of care<br>The ideal is assumed to be clinician visits that are more frequent as death approaches.	Numerator: Sum of home and office visits for the entire cohort in last two weeks	Denominator: Sum of home and office visits for the entire cohort in last six months	Exclusion: -	Performance standard: -	Excluded	Relevance to UK care settings	N/A	N/A
273	Keay,1994	Follow-up bereavement counseling is offered by the physician.	Numerator: -	Denominator: -	Exclusion: -	Performance standard: -	Included	N/A	09-DOMAIN 9 FAMILY CARE AND INVOLVEMENT	Process
274	National Hospice and Palliative Care Organization (NHPCO), 2006 Brown University, 2006	Inform and communicate about patient (Family evaluation of hospice care)<br>Questions:<br>D5: How often did the hospice team keep you or other family members informed about the patient's condition?<br>D7: Would you have wanted more information about what to expect while the patient was dying?	Numerator: Sum of 1 response to survey instrument by family member of deceased patient	Denominator: Number of items (=2)	Exclusion: Patients who are not enrolled in a hospice program or have disenrolled from a hospice program. Live discharged are excluded	Performance standard: -	Excluded	Relevance to long-term care settings	N/A	N/A

275	National Hospice and Palliative Care Organization (NHPCO), 2006 Brown University, 2006	Provide information about symptoms (Family evaluation of hospice care) Questions: B4: Did you want more information than you got about the medicines used to manage the patient's pain? B8: Did you want more information than you got about what was being done for the patient's trouble with breathing?	Numerator: Sum of 1 response to survey instrument by family member of deceased patient	Denominator: Number of items (=2)	Exclusion: Patients who are not enrolled in a hospice program or have disenrolled from a hospice program. Live discharged are excluded	Performance standard: -	Excluded	Relevance to long-term care settings	N/A	N/A
276	Lorenz, 2007 NEW van der Ploeg, 2008	Bereavement IF a VE's spouse or significant other dies, THEN the VE should be assessed for depression or thoughts of suicidality within 6 months, BECAUSE bereaved persons are at high risk of poor outcomes.	Numerator: -	Denominator: -	Exclusion: -	Performance standard: -	Excluded	Procedural Relevance	N/A	N/A
277	NEW Quality Markers, 2009	QM for primary care: They collate information of the quality of care provided to individuals after their death for audit purposes (e.g. using a tool such as the After Death Analysis from the Gold Standards Framework).	Proportion of carers and family members who receive support following the death of a patient	Numerator: - Denominator: -	Exclusion: -	Performance standard: -	Included	N/A	09-DOMAIN 9 FAMILY CARE AND INVOLVEMENT	Process
278	NEW ELCQuA, 2011	Relatives, carers and friends of the deceased, including other residents/patients and children, have access to effective bereavement services.	Numerator: Number of providers meeting 75% of NICE bereavement standards	Denominator: Total number of providers	Exclusion: -	Performance standard: Standards in NICE Supportive and Palliative Care Guidance are fully met by all relevant providers, and all care homes have policies in place to ensure other residents are supported following death	Included	N/A	09-DOMAIN 9 FAMILY CARE AND INVOLVEMENT	Outcome
279	NEW ELCQuA, 2011	Verification of death and care after death is sensitively delivered in line with best practice.	Numerator: Number of deaths using the care after death module of the LCP or equivalent	Denominator: Total number of deaths	Exclusion: -	Performance standard: -	Excluded	Relevance to UK care settings	N/A	N/A
280	NEW Quality Markers, 2009	QM for primary care: They have mechanisms in place to assess and document the needs of those approaching the end of life (e.g. use of the Gold Standards Framework or equivalent), and to discuss, record and, (where appropriate) communicate the wishes and preferences of those	Proportion of patients who die in their preferred place for care	Numerator: - Denominator: -	Exclusion: -	Performance standard: -	Included	N/A	03-DOMAIN 3 SETTING CARE GOALS AND ADVANCE PLANNING	Outcome

		approaching the end of life (advance care planning).								
281	NEW Quality Markers, 2009	QM for primary care: They collate information of the quality of care provided to individuals after their death for audit purposes (e.g. using a tool such as the After Death Analysis from the Gold Standards Framework).	Proportion of individuals who die in their preferred place	Numerator: -<br>Denominator: -	Exclusion: -	Performance standard: -	Included	N/A	03-DOMAIN 3 SETTING CARE GOALS<br>AND ADVANCE PLANNING	Outcome
282	NEW Quality Markers, 2009	QM for district/community nursing services. They have mechanisms in place to assess and document the needs of those approaching the end of life (e.g. use of the Gold Standards Framework or equivalent), and to discuss, record and, (where appropriate) communicate the wishes and preferences of those approaching the end of life (advance care planning).	Proportion of patients who die in their preferred place for care	Numerator: -<br>Denominator: -	Exclusion: -	Performance standard: -	Included	N/A	03-DOMAIN 3 SETTING CARE GOALS<br>AND ADVANCE PLANNING	Process
283	NEW Claessen, 2011	Generic aspects: Percentage of patients who died in the location of their preference<br>Patients have the right to die wherever they wish. The patient's wishes therefore prevail.	Numerator: The number of relatives who indicate that the patient died in the location of his/her preference	Denominator: The total number of relatives among whom this quality indicator was measured	Exclusion: -	Performance standard: -	Included	N/A	03-DOMAIN 3 SETTING CARE GOALS<br>AND ADVANCE PLANNING	Outcome
284	NEW ELCQuA, 2011<br><br>This indicator suits domain 7 and 8.	Individuals are offered an advance care plan.	Numerator: Number of patients who died who were registered to have an advance care plan in place plus number offered a care plan but without a plan in place.<br><br>Numerator: Number dying in preferred place of death.<br><br>Numerator: Number of patients who died in their own home or care home.<br><br>Numerator: Number of deaths in hospital within 3 days of admission by PCT or acute site		Exclusion: -<br><br>Exclusion: -<br><br>Exclusion: -<br><br>Exclusion: -	Performance standard: All patients/service users requiring end of life care or approaching end of life are offered advance care planning conversations<br><br>Performance standard: -<br><br>Performance standard: -<br><br>Performance standard: -	Excluded	Conceptualisation and/or Operationalisation	N/A	N/A

285	NEW Quality Markers, 2009	QM for primary care: They collate information of the quality of care provided to individuals after their death for audit purposes (e.g. using a tool such as the After Death Analysis from the Gold Standards Framework).	Proportion of individuals who die at home.	Numerator: -<br>Denominator: -	Exclusion: -	Performance standard: -	Included	N/A	06-DOMAIN 6 AVOIDING OVERLY AGGRESSIVE, BURDENSOME OR FUTILE TREATMENT	Process
286	Keay, 1994	Interventions not wanted by the patient are not performed.	Numerator: -	Denominator: -	Exclusion: Conflicting patient directives	Performance standard: > 80%	Included	N/A	03-DOMAIN 3 SETTING CARE GOALS AND ADVANCE PLANNING	Outcome
287	Lorenz, 2007	Decisions about life-sustaining treatment<br>IF a VE has documented treatment preferences to withhold or withdraw life-sustaining treatment (e.g., DNR order, no tube feeding, no hospital transfer), THEN these treatment preferences should be followed, BECAUSE medical care should aim to be consistent with a patient's preferences.	Numerator: -	Denominator: -	Exclusion: -	Performance standard: -	Included	N/A	03-DOMAIN 3 SETTING CARE GOALS AND ADVANCE PLANNING	Outcome
288	NEW Miyashita, 2008<br>Sato, 2008	Decision making and preference of care: Patient's preference of place of care.	Numerator: Number of patients for whom this indicator was documented on admission or within the last 2 weeks of the hospitalization	Denominator: All patients who died (retrospectively identified)	Exclusion: -	Performance standard: -	Included	N/A	02-DOMAIN 2 PERSON-CENTRED CARE, COMMUNICATION AND SHARED DECSION MAKING	Process
289	NEW Miyashita, 2008<br>Sato, 2008	Decision making and preference of care: Patient's insight of disease.	Numerator: Number of patients for whom this indicator was documented on admission or within the last 2 weeks of the hospitalization	Denominator: All patients who died (retrospectively identified)	Exclusion: -	Performance standard: -	Included	N/A	02-DOMAIN 2 PERSON-CENTRED CARE, COMMUNICATION AND SHARED DECSION MAKING	Process
290	NEW Miyashita, 2008<br>Sato, 2008	Family care: Family's preferred place of care.	Numerator: Number of patients for whom this indicator was documented on admission or within the last 2 weeks of the hospitalization	Denominator: All patients who died (retrospectively identified)	Exclusion: -	Performance standard: -	Included	N/A	09-DOMAIN 9 FAMILY CARE AND INVOLVEMENT	Process
291	NEW Miyashita, 2008<br>Sato, 2008	Family care: Family's preferences or expectations.	Numerator: Number of patients for whom this indicator was documented on admission or within the last 2 weeks of the hospitalization	Denominator: All patients who died (retrospectively identified)	Exclusion: -	Performance standard: -	Included	N/A	09-DOMAIN 9 FAMILY CARE AND INVOLVEMENT	Process

292	NEW Quality Markers, 2009	QM for primary care: They (GP practices) have mechanisms in place to assess and document the needs of those approaching the end of life (e.g. use of the Gold Standards Framework or equivalent), and to discuss, record and, (where appropriate) communicate the wishes and preferences of those approaching the end of life (advance care planning).	Proportion of individuals whose preferred place for care has been recorded	Numerator: - Denominator: -	Exclusion: -	Performance standard: -	Included	N/A	03-DOMAIN 3 SETTING CARE GOALS AND ADVANCE PLANNING	Process
293	NEW Quality Markers, 2009	QM for district/community nursing services. They have mechanisms in place to assess and document the needs of those approaching the end of life (e.g. use of the Gold Standards Framework or equivalent), and to discuss, record and, (where appropriate) communicate the wishes and preferences of those approaching the end of life (advance care planning).	Proportion of individuals whose preferred place for care has been recorded	Numerator: - Denominator: -	Exclusion: -	Performance standard: -	Included	N/A	03-DOMAIN 3 SETTING CARE GOALS AND ADVANCE PLANNING	Process
294	Mularski, 2006	Assessment within 24 hours of admission of the patient's capacity to make decisions.	Numerator: Total number of patients in the ICU with documentation of decisional capacity made within 24 hours of admission	Denominator: Total number of patients in the ICU for > 24 hours	Exclusion: -	Performance standard: -	Excluded	Relevance to long-term care settings	N/A	N/A
295	Nelson, 2006, Mularski, 2006	Medical decision maker: Percentage of patients with documentation of status of identification of health care proxy (or other appropriate surrogate).	Numerator: Number of patients with documentation of status of identification of health care proxy (or other appropriate surrogate)	Denominator: Total number of patients admitted to the ICU within the last 24 hours	Exclusion: -	Performance standard: -	Included	N/A	03-DOMAIN 3 SETTING CARE GOALS AND ADVANCE PLANNING	Process
296	NEW Miyashita, 2008 Sato, 2008	Family care: Key person involved in patient care.	Numerator: Number of patients for whom this indicator was documented on admission or within the last 2 weeks of the hospitalization	Denominator: All patients who died (retrospectively identified)	Exclusion: -	Performance standard: -	Included	N/A	09-DOMAIN 9 FAMILY CARE AND INVOLVEMENT	Process
297	NEW Schenck, 2010	Ethical and legal aspects of care: Percent of patients with contact information for surrogate decision maker in the chart or documentation that there is no surrogate.	Numerator: Number of patients with surrogate contact info ="Y" or Discussion of no surrogate = "Y"	Denominator: Number of Patients	Exclusion: -	Performance standard: -	Included	N/A	03-DOMAIN 3 SETTING CARE GOALS AND ADVANCE PLANNING	Process

298	NEW Schenck, 2010	Ethical and legal aspects of care: Percent of patients with impaired decision making (dementia, coma or other impairment) that have documentation of surrogate decision maker in chart within 2 days of recognition of impaired decision making.	Numerator: Number of patients with (0<=Surrogate document time <=2days) and (Dementia="Y" or Confused-sedated-nonverbal="Y") (Surrogate date = Date of documentation if chart has a surrogate decision maker, or date of documentation of no surrogate if chart contains contact info of surrogate or discussion of no surrogate is recorded Surrogate document time = surrogate date – admission date)	Denominator: Number of patients with dementia="Y" or confused-sedated-nonverbal="Y"	Exclusion: -	Performance standard: -	Included	N/A	03-DOMAIN 3 SETTING CARE GOALS AND ADVANCE PLANNING	Process
299	Nelson, 2006, Mularski, 2006	Advance directive: percentage of patients with documentation of advance directive status on or before day 1 of the ICU admission.	Numerator: Number of patients who have documentation of advance directive status on or before day 1of the ICU admission	Denominator: Total number of patients with an ICU length of stay > 5 days	Exclusion: Patients discharged (or transferred out of the ICU) on or before day 1 of ICU admission Patients expired on or before day 1 of ICU admission Patients with decisional capacity	Performance standard: -	Excluded	Relevance to long-term care settings	N/A	N/A
300	Nelson, 2006	Resuscitation status: percentage of patients with documentation of resuscitation status.	Numerator: Number of patients with documentation of resuscitation status	Denominator: Total number of patients admitted to the ICU within the last 24 hours	Exclusion: -	Performance standard: -	Excluded	Relevance to long-term care settings	N/A	N/A
301	Keay, 1994	Documentation of patients wishes or advance directive is present.	Numerator: -	Denominator: -	Exclusion: Patient was always incapacitated and without advance directive or legal proxy	Performance standard: 100%	Included	N/A	03-DOMAIN 3 SETTING CARE GOALS AND ADVANCE PLANNING	Process
302	Lorenz, 2007	ACP documented ALL VEs should have in the outpatient chart patient's surrogate decision maker, or documentation of a discussion to identify or search for a surrogate decision maker, BECAUSE, advance directives and discussions about surrogate decisions makers facilitate identification of surrogate decision makers and decision making on	Numerator: -	Denominator: -	Exclusion: -	Performance standard: -	Included	N/A	03-DOMAIN 3 SETTING CARE GOALS AND ADVANCE PLANNING	Process

		behalf of a patient who has lost decision-making capacity.								
303	Lorenz, 2007	Advance directive and surrogate continuity<br>IF a VE is admitted to the hospital or nursing home, THEN within 48 hours of admission, the medical record should contain the patient's surrogate decision maker or documentation of a discussion to identify or search for surrogate decision maker, BECAUSE specification of a surrogate decision maker facilitates decision making for patients at risk of losing decision making capacity.	Numerator: -	Denominator: -	Exclusion: -	Performance standard: -	Included	N/A	03-DOMAIN 3 SETTING CARE GOALS<br>AND ADVANCE PLANNING	Process
304	Lorenz, 2007	Care-preference documentation<br>IF a VE with severe dementia is admitted to the hospital and survives 48 hours, THEN within 48 hours of admission, the medical record should document that the patient's preferences for care have been considered or an attempt was made to identify them, BECAUSE patient's values and preferences should guide life-sustaining care.	Numerator: -	Denominator: -	Exclusion: -	Performance standard: -	Included	N/A	04-DOMAIN 4 CONTINUITY OF CARE	Process
305	Lorenz, 2007	Care-preference documentation<br>IF a VE is admitted to the ICU and survives 48 hours, THEN within 48 hours of ICU admission, the medical record should document that the patient's preferences for care have been considered or an attempt was made to identify them, BECAUSE patient's values and preferences should guide life-sustaining care.	Numerator: -	Denominator: -	Exclusion: -	Performance standard: -	Excluded	Relevance to long-term care settings	N/A	N/A

306	Lorenz, 2007	Decisions about life-sustaining treatment IF a VE with decision-making capacity has orders in the hospital or nursing home to withhold or withdraw a life-sustaining treatment (e.g., DNR order), THEN the medical record should document patient participation in the decision or why the patient did not participate, BECAUSE the values of patients with decision-making capacity who wish to participate should guide important care decisions.	Numerator: -	Denominator: -	Exclusion: -	Performance standard: -	Included	N/A	03-DOMAIN 3 SETTING CARE GOALS AND ADVANCE PLANNING	Process
307	Lorenz, 2007	Mechanical ventilation preference IF a hospitalized VE requires mechanical ventilation for longer than 48 hours, THEN within 48 hours of the initiation of the mechanical, the medical record should document the goals of care and the patient's preference for mechanical ventilation or why this information is unavailable, BECAUSE mechanical ventilation should be performed only when it is consistent with a patient's goals, and early consideration of prognosis and preferences will guide care to be consistent with the patient's values.	Numerator: -	Denominator: -	Exclusion: -	Performance standard: -	Excluded	Procedural Relevance	N/A	N/A
308	Lorenz, 2007	Goals of care surrogate discussion IF a VE dies an expected death with metastatic cancer, oxygen-dependent pulmonary disease, New York Heart Association (NYHA) Class III to IV congestive heart failure (CHF). end-stage (stage IV) renal disease, or dementia, THEN the chart should document discussion of the medical condition and goals for treatment with a designated surrogate, the patient's preferences for not involving a designated surrogate in discussions, or a note that a surrogate decision maker is unavailable within 6 months before death, BECAUSE temporary decisional incapacity is common in these health states, and therefore, surrogates are at risk of being called upon to assist in achieving	Numerator: -	Denominator: -	Exclusion: -	Performance standard: -	Included	N/A	02-DOMAIN 2 PERSON-CENTRED CARE, COMMUNICATION AND SHARED DECSION MAKING	Process

		patient preferences, yet it is not routine for physicians to involve surrogates in care planning.								
309	Lorenz, 2007	Gastrostomy tube placement<br>IF a VE with dementia has a gastrostomy or jejunum tube placed, THEN before placement, the medical record should document patient preferences concerning tube feeding; discussion of patient preferences or best interests if the patient is decisionally incapacitated and a surrogate decision maker is available; or use of a formal decision mechanism is the patient is decisionally incapacitated and a surrogate decision maker is not available, BECAUSE many patients would not want to receive tube feeding to maintain survival is a persistent severely compromised health state, and decisions are often made to place gastrostomy tubes when patients can no longer participate in decisions.	Numerator: -	Denominator: -	Exclusion: -	Performance standard: -	Included	N/A	03-DOMAIN 3 SETTING CARE GOALS AND ADVANCE PLANNING	Process
310	Saliba, 2004	ALL residents, within 2 weeks of NH admission, should have physician notes or orders documenting a discussion or decision concerning all of the following: resuscitation status, hospital transfers, and advance directives, unless there is documentation that the resident is not capable of understanding and surrogate could not be located. This information should remain available in the chart throughout the resident's stay.	Numerator: -	Denominator: -	Exclusion: When there is documentation that the resident is not capable of understanding and surrogate could not be located	Performance standard: 100%	Included	N/A	03-DOMAIN 3 SETTING CARE GOALS AND ADVANCE PLANNING	Process

311	NEW Miyashita, 2008	Decision making and preference of care: Patient's preference of care or advance directives.	Numerator: Number of patients for whom this indicator was documented on admission or within the last 2 weeks of the hospitalization	Denominator: All patients who died (retrospectively identified)	Exclusion: -	Performance standard: -	Included	N/A	03-DOMAIN 3 SETTING CARE GOALS AND ADVANCE PLANNING	Process
312	NEW Sato, 2008	Decision making and preference of care: Discussion with patient about goals of care.	Numerator: Number of patients for whom this indicator was documented on admission or within the last 2 weeks of the hospitalization	Denominator: All patients who died (retrospectively identified)	Exclusion: -	Performance standard: -	Included	N/A	02-DOMAIN 2 PERSON-CENTRED CARE, COMMUNICATION AND SHARED DECSION MAKING	Process
313	NEW Sato, 2008	Decision making and preference of care: Discussion with patient about do-not-resuscitate order.	Numerator: Number of patients for whom this indicator was documented on admission or within the last 2 weeks of the hospitalization	Denominator: All patients who died (retrospectively identified)	Exclusion: -	Performance standard: -	Included	N/A	02-DOMAIN 2 PERSON-CENTRED CARE, COMMUNICATION AND SHARED DECSION MAKING	Process
314	NEW Miyashita, 2008 Sato, 2008	Family care: Discussion with family about do-not-resuscitate order.	Numerator: Number of patients for whom this indicator was documented on admission or within the last 2 weeks of the hospitalization	Denominator: All patients who died (retrospectively identified)	Exclusion: -	Performance standard: -	Included	N/A	09-DOMAIN 9 FAMILY CARE AND INVOLVEMENT	Process
315	NEW Miyashita, 2008, omitted in Sato, 2008	Psychosocial and spiritual concerns: Patient's preference for bowel and bladder excretion.	Numerator: Number of patients for whom this indicator was documented on admission or within the last 2 weeks of the hospitalization	Denominator: All patients who died (retrospectively identified)	Exclusion: -	Performance standard: -	Excluded	Procedural Relevance	N/A	N/A
316	NEW Schenck, 2010	Ethical and legal aspects of care: Percent of patients with chart documentation of preferences for life sustaining treatments.	Numerator: Number of patients with documentation = "Y"	Denominator: Number of Patients	Exclusion: -	Performance standard: -	Included	N/A	03-DOMAIN 3 SETTING CARE GOALS AND ADVANCE PLANNING	Process
317	NEW Schenck, 2010	Ethical and legal aspects of care: Percent of patients with chart documentation of an advanced directive (living will or health care power of attorney) or discussion that there is no advanced directive.	Numerator: Number of patients with documentation of advanced directive="Y" or Discussion of no advanced directive = "Y"	Denominator: Number of Patients	Exclusion: -	Performance standard: -	Included	N/A	03-DOMAIN 3 SETTING CARE GOALS AND ADVANCE PLANNING	Process
318	NEW Schenck, 2010	Adverse Events: Selected number of occurrences per 100 patient days (falls, medication errors, DME concerns, and patient or family complaints).	Numerator: The total number of occurrences reported in the time period	Denominator: Total number of patient days in the time period	Exclusion: -	Performance standard: -	Included	N/A	07-DOMAIN 7 OPTIMAL TREATMENT OF SYMPTOMS AND PROVIDING COMFORT	Outcome

319	NEW Quality Markers, 2009	QM for acute hospitals: They offer care plans to all patients who are approaching the end of life.	Proportion of all deceased patients who had an end of life care plan (or documentation that a care plan had been offered but declined)	Numerator: -<br>Denominator: -	Exclusion: -	Performance standard: -	Excluded	Relevance to long-term care settings	N/A	N/A
320	NEW Quality Markers, 2009	QM for community hospitals: They offer care plans to all patients who are approaching the end of life.	Proportion of all deceased patients who had an end of life care plan (or documentation that a care plan had been offered but declined)	Numerator: -<br>Denominator: -	Exclusion: -	Performance standard: -	Excluded	Relevance to long-term care settings	N/A	N/A
321	NEW Lorenz, 2009	Information and care planning<br>IF a patient with advanced cancer dies an expected death, THEN there should be documentation of an advance directive or a surrogate decision maker in the medical record.	Numerator: -	Denominator: -	Exclusion: -	Performance standard: -	Included	N/A	03-DOMAIN 3 SETTING CARE GOALS<br>AND ADVANCE PLANNING	Process
322	NEW Lorenz, 2009	Information and care planning<br>IF a patient with advanced cancer is admitted to the ICU and survives 48 hours, THEN within 48 hours of ICU admission, the medical record should document the patient's preferences for care or attempt to identify them.	Numerator: -	Denominator: -	Exclusion: -	Performance standard: -	Excluded	Relevance to long-term care settings	N/A	N/A
323	NEW Lorenz, 2009	Information and care planning IF a patient with advanced cancer is mechanically ventilated in the ICU, THEN within 48 hours of admission to the ICU, the medical record should document the patient's preference for mechanical ventilation or why this information is unavailable.	Numerator: -	Denominator: -	Exclusion: -	Performance standard: -	Excluded	Relevance to long-term care settings	N/A	N/A
324	NEW Claessen, 2011	Generic aspects: Presence of documentation concerning the desired care and treatment at the end of life<br>The patient's wishes with regard to care and treatment at the end of life must be documented.	Numerator: The number of patients for whom the desired care and treatment at the end of life is documented	Denominator: Total number of patient files consulted	Exclusion: -	Performance standard: -	Included	N/A	03-DOMAIN 3 SETTING CARE GOALS<br>AND ADVANCE PLANNING	Process
325	NEW ELCQuA, 2011	Individuals have an agreed care plan.	Numerator: Number of deceased patients with care plan in place	Denominator: Total deaths for same time period	Exclusion: -	Performance standard: Care plans in place for all patients approaching the end of life	Included	N/A	03-DOMAIN 3 SETTING CARE GOALS<br>AND ADVANCE PLANNING	Process
326	NEW ELCQuA, 2011	Patients with an agreed Do Not Attempt Resuscitation policy in place have their preferences respected.	Numerator: Number of patients with a DNAR in place where	Denominator: Total number of deaths where a DNAR is in place	Exclusion: -	Performance standard: -	Included	N/A	03-DOMAIN 3 SETTING CARE GOALS	Outcome

			resuscitation is attempted						AND ADVANCE PLANNING	
327	Price, 2014<br><br>Centres for Medicare and Medicaid services, 2015	Communication with family<br><br>Multi-item measure. "While your family member was in hospice care..." P1: "How often did the hospice team keep you informed about when they would arrive to care for your family member?" P2: "How often did the hospice team explain things in a way that was easy to understand?" P3: "How often did the hospice team listen carefully to you when you talked with them about problems with your family member's hospice care?" P4: "How often did the hospice team keep you informed about your family member's condition?" P5: "How often did the hospice team listen carefully to you? P6: "How often did anyone from the hospice team give you confusing or contradictory information about your family member's condition or care?"	Numerator<br><br>CAHPS Hospice Survey measures are calculated using top-box scoring. The top-box score refers to the percentage of caregiver respondents that give the most positive response. For questions P1 through P5 in this measure, the top box numerator is the number of respondents who answer "Always." For question P6, the top box numerator is the number of respondents who answer "Never." Top box scores for each survey question within the measure are adjusted for mode of survey administration (at the individual respondent level) and case mix (at the hospice level), and then averaged to calculate the overall hospice-level measure score.	Denominator<br><br>The top box denominator is the number of respondents who answer at least one question in the multi-item measure (i.e., one of P1 through P6).	Exclusion<br><br>The hospice patient is still alive -The decedent's age at death was less than 18 -The decedent died within 48 hours of his/her last admission to hospice care -The decedent had no caregiver of record -The decedent had a caregiver of record, but the caregiver does not have a U.S. or U.S. Territory home address - The decedent had no caregiver other than a nonfamilial legal guardian - The decedent or caregiver requested that they not be contacted (i.e., by signing a no publicity request while under the care of hospice or otherwise directly requesting not to be contacted) -The caregiver is institutionalized, has mental/physical incapacity, has a language barrier, or is deceased - The caregiver reports on the survey that he or she "never" oversaw or took part in decedent's hospice care	Performance standard: -	Included	N/A	09-DOMAIN 9 FAMILY CARE AND INVOLVEMENT	Process

328	Price, 2014<br><br>Centres for Medicare and Medicaid services, 2016	Getting timely help<br><br>Multi-item measure P1: "While your family member was in hospice care, when you or your family member asked for help from the hospice team, how often did you get help as soon as you needed it?" P2: "How often did you get the help you needed from the hospice team during evenings, weekends, or holidays?"	Numerator<br><br>CAHPS Hospice Survey measures are calculated using top-box scoring. The top-box score refers to the percentage of caregiver respondents that give the most positive response. The top box numerator is the number of respondents who answer "Always." Top box scores for each survey question within the measure are adjusted for mode of survey administration (at the individual respondent level) and case mix (at the hospice level), and then averaged to calculate the overall hospice-level measure score.	Denominator<br><br>The top box denominator is the number of respondents who answer at least one question in the multi-item measure (i.e., one of P1 or P2).	Exclusion<br><br>Exclusions from the denominator: - The hospice patient is still alive -The decedent's age at death was less than 18 -The decedent died within 48 hours of his/her last admission to hospice care -The decedent had no caregiver of record -The decedent had a caregiver of record, but the caregiver does not have a U.S. or U.S. Territory home address - The decedent had no caregiver other than a nonfamilial legal guardian - The decedent or caregiver requested that they not be contacted (i.e., by signing a no publicity request while under the care of hospice or otherwise directly requesting not to be contacted) -The caregiver is institutionalized, has mental/physical incapacity, has a language barrier, or is deceased - The caregiver reports on the survey that he or she "never" oversaw or took part in decedent's hospice care	Performance standard: -	Included	N/A	04-DOMAIN 4 CONTINUITY OF CARE	Process

329	Price, 2014<br><br>Centres for Medicare and Medicaid services, 2017	Treating patient with respect<br><br>Multi-item measure P1: "While your family member was in hospice care, how often did the hospice team treat your family member with dignity and respect?" P2: "While your family member was in hospice care, how often did you feel that the hospice team really cared about your family member?	Numerator<br><br>CAHPS Hospice Survey measures are calculated using top-box scoring. The top-box score refers to the percentage of caregiver respondents that give the most positive response. For both questions in this measure, the top box numerator is the number of respondents who answer "Always." Top box scores for each survey question within the measure are adjusted for mode of survey administration (at the individual respondent level) and case mix (at the hospice level), and then averaged to calculate the overall hospice-level measure score.	Denominator<br><br>The top box denominator is the number of respondents who answer at least one question in the multi-item measure (i.e., one of P1 or P2).	Exclusion<br><br>The hospice patient is still alive -The decedent's age at death was less than 18 -The decedent died within 48 hours of his/her last admission to hospice care -The decedent had no caregiver of record -The decedent had a caregiver of record, but the caregiver does not have a U.S. or U.S. Territory home address - The decedent had no caregiver other than a nonfamilial legal guardian - The decedent or caregiver requested that they not be contacted (i.e., by signing a no publicity request while under the care of hospice or otherwise directly requesting not to be contacted) -The caregiver is institutionalized, has mental/physical incapacity, has a language barrier, or is deceased - The caregiver reports on the survey that he or she "never" oversaw or took part in decedent's hospice care	Performance standard: -	Included	N/A	02-DOMAIN 2 PERSON-CENTRED CARE, COMMUNICATION AND SHARED DECSION MAKING	Process

330	Price, 2014<br><br>Centres for Medicare and Medicaid services, 2018	Emotional and Spiritual support<br><br>Multi-item measure P1: "While your family member was in hospice care, how much emotional support did you get from the hospice team?" P2: "In the weeks after your family member died, how much emotional support did you get from the hospice team?" P3: "Support for religious or spiritual beliefs includes talking, praying, quiet time, or other ways of meeting your religious or spiritual needs. While your family member was in hospice care, how much support for your religious and spiritual beliefs did you get from the hospice team?"	Numerator<br><br>CAHPS Hospice Survey measures are calculated using top-box scoring. The top-box score refers to the percentage of caregiver respondents that give the most positive response. For all questions in this measure, the top box numerator is the number of respondents who answer "Right amount." Top box scores for each survey question within the measure are adjusted for mode of survey administration (at the individual respondent level) and case mix (at the hospice level), and then averaged to calculate the overall hospice-level measure score.	Denominator<br><br>The top box denominator is the number of respondents who answer at least one question in the multi-item measure (i.e., one of P1 through P3).	Exclusion<br><br>The hospice patient is still alive -The decedent's age at death was less than 18 -The decedent died within 48 hours of his/her last admission to hospice care -The decedent had no caregiver of record -The decedent had a caregiver of record, but the caregiver does not have a U.S. or U.S. Territory home address -The decedent had no caregiver other than a nonfamilial legal guardian -The decedent or caregiver requested that they not be contacted (i.e., by signing a no publicity request while under the care of hospice or otherwise directly requesting not to be contacted) -The caregiver is institutionalized, has mental/physical incapacity, has a language barrier, or is deceased -The caregiver reports on the survey that he or she "never" oversaw or took part in decedent's hospice care	Performance standard: -	Included	N/A	08-DOMAIN 8 PSYCHOSOCIAL AND SPIRITUAL SUPPORT	Process

331	Price, 2014<br><br>Centres for Medicare and Medicaid services, 2019	Help for Pain and Symptoms<br><br>Multi-item measure P1: "Did your family member get as much help with pain as he or she needed?" P2: "How often did your family member get the help he or she needed for trouble breathing?" P3: "How often did your family member get the help he or she needed for trouble with constipation?" P4: "How often did your family member receive the help he or she needed from the hospice team for feelings of anxiety or sadness?"	Numerator<br><br>CAHPS Hospice Survey measures are calculated using top-box scoring. The top-box score refers to the percentage of caregiver respondents that give the most positive response. For question P1, the top box numerator is the number of respondents who answer "Yes, definitely." For questions P2, P3 and P4, the top box numerator is the number of respondents who answer "Always." Top box scores for each survey question within the measure are adjusted for mode of survey administration (at the individual respondent level) and case mix (at the hospice level), and then averaged to calculate the overall hospice-level measure score.	Denominator<br><br>The top box denominator is the number of respondents who answer at least one question in the multi-item measure (i.e., one of P1 through P4).	Exclusion<br><br>The hospice patient is still alive -The decedent's age at death was less than 18 -The decedent died within 48 hours of his/her last admission to hospice care -The decedent had no caregiver of record -The decedent had a caregiver of record, but the caregiver does not have a U.S. or U.S. Territory home address - The decedent had no caregiver other than a nonfamilial legal guardian - The decedent or caregiver requested that they not be contacted (i.e., by signing a no publicity request while under the care of hospice or otherwise directly requesting not to be contacted) -The caregiver is institutionalized, has mental/physical incapacity, has a language barrier, or is deceased - The caregiver reports on the survey that he or she "never" oversaw or took part in decedent's hospice care	Performance standard: -	Included	N/A	07-DOMAIN 7 OPTIMAL TREATMENT OF SYMPTOMS AND PROVIDING COMFORT	Process

332	Price, 2014<br><br>Centres for Medicare and Medicaid services, 2020	Training family to care for patient<br><br>Multi-item measure P1: Did the hospice team give you the training you needed about what side effects to watch for from pain medication? P2: Did the hospice team give you the training you needed about if and when to give more pain medicine to your family member? P3: Did the hospice team give you the training you needed about how to help your family member if he or she had trouble breathing? P4: Did the hospice team give you the training you needed about what to do if your family member became restless or agitated? P5: Side effects of pain medicine include things like sleepiness. Did any member of the hospice team discuss side effects of pain medicine with your or your family member?	Numerator<br><br>CAHPS Hospice Survey measures are calculated using top-box scoring. The top-box score refers to the percentage of caregiver respondents that give the most positive response. For all questions in this measure, the top box numerator is the number of respondents who answer "Yes, definitely." Top box scores for each survey question within the measure are adjusted for mode of survey administration (at the individual respondent level) and case mix (at the hospice level), and then averaged to calculate the overall hospice-level measure score.	Denominator<br><br>The top box denominator is the number of respondents who answer at least one question in the multi-item measure (i.e., one of P1 through P5).	Exclusion<br><br>The hospice patient is still alive -The decedent's age at death was less than 18 -The decedent died within 48 hours of his/her last admission to hospice care -The decedent had no caregiver of record -The decedent had a caregiver of record, but the caregiver does not have a U.S. or U.S. Territory home address - The decedent had no caregiver other than a nonfamilial legal guardian - The decedent or caregiver requested that they not be contacted (i.e., by signing a no publicity request while under the care of hospice or otherwise directly requesting not to be contacted) -The caregiver is institutionalized, has mental/physical incapacity, has a language barrier, or is deceased - The caregiver reports on the survey that he or she "never" oversaw or took part in decedent's hospice care	Performance standard: -	Included	N/A	09-DOMAIN 9 FAMILY CARE AND INVOLVEMENT	Process

333	Price, 2014<br><br>Centres for Medicare and Medicaid services, 2021	Rating of this Hospice<br><br>Individual survey item asking respondents: "Using any number from 0 to 10, where 0 is the worst hospice care possible and 10 is the best hospice care possible, what number would you use to rate your family member's hospice care?" 0-10 rating scale with 0=Worst hospice care possible and 10=Best hospice care possible	Numerator<br><br>The top box numerator is the number of respondents in the hospice who answer "9" or "10." Top box scores for the measure are adjusted for mode of survey administration (at the individual respondent level) and case mix (at the hospice level) to calculate the overall hospice-level measure score.	Denominator<br><br>The top box denominator is the total number of respondents in the hospice who answered the item.	Exclusion<br><br>The hospice patient is still alive -The decedent's age at death was less than 18 -The decedent died within 48 hours of his/her last admission to hospice care -The decedent had no caregiver of record -The decedent had a caregiver of record, but the caregiver does not have a U.S. or U.S. Territory home address -The decedent had no caregiver other than a nonfamilial legal guardian -The decedent or caregiver requested that they not be contacted (i.e., by signing a no publicity request while under the care of hospice or otherwise directly requesting not to be contacted) -The caregiver is institutionalized, has mental/physical incapacity, has a language barrier, or is deceased -The caregiver reports on the survey that he or she "never" oversaw or took part in decedent's hospice care	Performance standard: -	Excluded	Relevance to long-term care settings	N/A	N/A

334	Price, 2014<br><br>Centres for Medicare and Medicaid services, 2022	Willing to recommend this Hospice<br><br>Individual survey item asking respondents: “Would you recommend this hospice to your friends and family?”	Numerator<br><br>The top box numerator is the number of respondents in a hospice program who responded “Definitely yes.” Top box scores for the measure are adjusted for mode of survey administration (at the individual respondent level) and case mix (at the hospice level) to calculate the overall hospice-level measure score.	Denominator<br><br>The top box denominator is the total number of respondents in the hospice that answered the item.	Exclusion<br><br>The hospice patient is still alive -The decedent’s age at death was less than 18 -The decedent died within 48 hours of his/her last admission to hospice care -The decedent had no caregiver of record -The decedent had a caregiver of record, but the caregiver does not have a U.S. or U.S. Territory home address - The decedent had no caregiver other than a nonfamilial legal guardian - The decedent or caregiver requested that they not be contacted (i.e., by signing a no publicity request while under the care of hospice or otherwise directly requesting not to be contacted) -The caregiver is institutionalized, has mental/physical incapacity, has a language barrier, or is deceased - The caregiver reports on the survey that he or she “never” oversaw or took part in decedent’s hospice care	Performance standard: -	Excluded	Relevance to long-term care settings	N/A	N/A

335	Palliative Care Outcomes Collaboration, 2015	Time from date ready for care to episode start<br><br>Time from date ready for care to episode start is calculated as the time in days between the date ready for care and the episode start	Numerator/Denominator: Percentage of patients whose episode commences the day of, or the day following the date ready for care	Denominator: -	Exclusion: -	Performance standard: 90%	Included	N/A	05-DOMAIN 5 PROGNOSTICATIO N AND TIMELY RECOGNITION OF DYING	Process
336	Palliative Care Outcomes Collaboration, 2016	Time in the unstable phase<br><br>Time in the unstable phase is calculated as the difference between the phase start date and the phase end date.	Percentage of patients in the unstable phase for 3 days or less	Denominator: -	Exclusion: -	Performance standard: 90%	Included	N/A	05-DOMAIN 5 PROGNOSTICATIO N AND TIMELY RECOGNITION OF DYING	Process
337	Palliative Care Outcomes Collaboration, 2017	Change in pain<br><br>Change in pain is calculated by the difference in pain score from the beginning of a phase to the end of phase and is calculated using both PCPSS pain and SAS pain measures.	Percentage of patients with absent or mild PCPSS pain at the beginning of their phase of palliative care have absent or mild PCPSS pain at the end of the phase<br><br>Percentage of patients with moderate or severe PCPSS pain at the beginning of their phase of palliative care have absent or mild PCPSS pain at the end of the phase<br><br>Percentage of patients with absent or mild SAS pain at the beginning of their phase of palliative care have absent or mild SAS pain at the end of the phase<br><br>Percentage of patients with moderate or severe SAS pain at the beginning of their phase of palliative care have absent or mild SAS pain at the end of the phase	Denominator: -	Exclusion: -	Performance standard: 90%<br><br>Performance standard: 60%<br><br>Performance standard: 90%<br><br>Performance standard: 60%	Included	N/A	07-DOMAIN 7 OPTIMAL TREATMENT OF SYMPTOMS AND PROVIDING COMFORT	Outcome

338	National Institute for Health and Care Excellence, 2017	Adults who have signs and symptoms that suggest they may be in the last days of life are monitored for further changes to help determine if they are nearing death, stabilising or recovering.<br><br>Evidence of local arrangements and systems to ensure that it is recognised when an adult may be entering the last days of life.	Numerator: -	Denominator: -	Exclusion: -	Performance standard: -	Excluded	Conceptualisation and/or Operationalisation	N/A	N/A
339	National Institute for Health and Care Excellence, 2017	Adults who have signs and symptoms that suggest they may be in the last days of life are monitored for further changes to help determine if they are nearing death, stabilising or recovering.<br><br>Evidence of local arrangements and systems to monitor signs and symptoms of adults thought to be in the last days of life, and to review changes in a person's condition to help determine if they are nearing death, stabilising or recovering.	Numerator: -	Denominator: -	Exclusion: -	Performance standard: -	Excluded	Conceptualisation and/or Operationalisation	N/A	N/A
340	National Institute for Health and Care Excellence, 2017	Adults who have signs and symptoms that suggest they may be in the last days of life are monitored for further changes to help determine if they are nearing death, stabilising or recovering.<br><br>Proportion of adult deaths with documented evidence that it was recognised that the person was in the last days of life.	Numerator<br><br>The number in the denominator in which the care records show it was recognised that the adult was in the last days of life.	Denominator<br><br>The number of adult deaths	Exclusion: -	Performance standard: -	Included	N/A	05-DOMAIN 5 PROGNOSTICATIO N AND TIMELY RECOGNITION OF DYING	Process
341	National Institute for Health and Care Excellence, 2017	Adults who have signs and symptoms that suggest they may be in the last days of life are monitored for further changes to help determine if they are nearing death, stabilising or recovering.<br><br>Proportion of adults recognised as being in the last days of life with documented evidence that their signs and symptoms were monitored at least daily.	Numerator<br><br>The number in the denominator in which the care records show evidence of monitoring of signs and symptoms at least daily	Denominator<br><br>The number of adult deaths	Exclusion: -	Performance standard: -	Included	N/A	07-DOMAIN 7 OPTIMAL TREATMENT OF SYMPTOMS AND PROVIDING COMFORT	Process

342	National Institute for Health and Care Excellence, 2017	Adults who have signs and symptoms that suggest they may be in the last days of life are monitored for further changes to help determine if they are nearing death, stabilising or recovering.<br><br>Proportion of adults thought to be in the last days of life given care appropriate to whether they were nearing death, stabilising or recovering.	Numerator	Denominator	Exclusion: -	Performance standard: -	Included	N/A	07-DOMAIN 7 OPTIMAL TREATMENT OF SYMPTOMS AND PROVIDING COMFORT	Outcome
343	National Institute for Health and Care Excellence, 2017	Adults in the last days of life, and the people important to them, are given opportunities to discuss, develop and review an individualised care plan<br><br>Evidence of local arrangements to ensure that adults in the last days of life, and the people important to them, are given opportunities to discuss and develop individualised care plans	Numerator: -	Denominator: -	Exclusion: -	Performance standard: -	Excluded	Conceptualisation and/or Operationalisation	N/A	N/A
344	National Institute for Health and Care Excellence, 2017	Adults in the last days of life, and the people important to them, are given opportunities to discuss, develop and review an individualised care plan<br><br>Evidence of local arrangements to ensure that adults in the last days of life have their individualised care plans reviewed	Numerator: -	Denominator: -	Exclusion: -	Performance standard: -	Excluded	Conceptualisation and/or Operationalisation	N/A	N/A
345	National Institute for Health and Care Excellence, 2017	Adults in the last days of life, and the people important to them, are given opportunities to discuss, develop and review an individualised care plan<br><br>Proportion of adults recognised as being in the last days of life, and the people important to them, who are given opportunities to discuss and develop an individualised care plan	Numerator:<br><br>the number in the denominator with care records that show the person who was in the last days of life, and the people important to them, were given opportunities to discuss and develop an individualised care plan	Denominator:<br><br>the number of adults recognised as being in the last days of life	Exclusion: -	Performance standard: -	Included	N/A	03-DOMAIN 3 SETTING CARE GOALS AND ADVANCE PLANNING	Process
346	National Institute for Health and Care Excellence, 2017	Adults in the last days of life, and the people important to them, are given opportunities to discuss, develop and review an individualised care plan<br><br>Proportion of adults recognised as	Numerator:<br><br>the number in the denominator with an individualised care plan	Denominator:<br><br>number of adults recognised as being in the last days of life	Exclusion: -	Performance standard: -	Included	N/A	03-DOMAIN 3 SETTING CARE GOALS AND ADVANCE PLANNING	Process

		being in the last days of life who have an individualised care plan									
347	National Institute for Health and Care Excellence, 2017	Adults in the last days of life, and the people important to them, are given opportunities to discuss, develop and review an individualised care plan<br><br>Proportion of adults recognised as being in the last days of life whose individualised care plan was followed	Numerator:<br><br>the number in the denominator whose individualised care plan was followed	Denominator:<br><br>the number of adults recognised as being in the last days of life	Exclusion: -	Performance standard: -	Included	N/A	03-DOMAIN 3 SETTING CARE GOALS AND ADVANCE PLANNING	Process
348	National Institute for Health and Care Excellence, 2017	Adults in the last days of life, and the people important to them, are given opportunities to discuss, develop and review an individualised care plan<br><br>Proportion of adults who feel they have choice and control over their care in the last days of life	Numerator: -	Denominator: -	Exclusion: -	Performance standard: -	Included	N/A	03-DOMAIN 3 SETTING CARE GOALS AND ADVANCE PLANNING	Outcome
349	National Institute for Health and Care Excellence, 2017	Adults in the last days of life who are likely to need symptom control are prescribed anticipatory medicines with individualised indications for use, dosage and route of administration<br><br>Evidence of local arrangements to ensure that adults in the last days of life are assessed for likely symptoms and are prescribed anticipatory medicines	Numerator: -	Denominator: -	Exclusion: -	Performance standard: -	Excluded	Conceptualisation and/or Operationalisation	N/A	N/A
350	National Institute for Health and Care Excellence, 2017	Adults in the last days of life who are likely to need symptom control are prescribed anticipatory medicines with individualised indications for use, dosage and route of administration<br><br>Proportion of adults recognised as being in the last days of life who have their prescribing needs assessed for symptoms likely to occur in their last days of life	Numerator:<br><br>the number in the denominator whose prescribing needs have been assessed for symptoms likely to occur in the last days of life	Denominator:<br><br>number of adults recognised as being in the last days of life	Exclusion: -	Performance standard: -	Included	N/A	07-DOMAIN 7 OPTIMAL TREATMENT OF SYMPTOMS AND PROVIDING COMFORT	Process

351	National Institute for Health and Care Excellence, 2017	Adults in the last days of life who are likely to need symptom control are prescribed anticipatory medicines with individualised indications for use, dosage and route of administration<br><br>Proportion of adults recognised as being in the last days of life prescribed anticipatory medicines with individualised indications for use, dosage and route of administration	Numerator:<br><br>the number in the denominator with care records that show anticipatory medicines have been prescribed with individualised indications for use, dosage and route of administration	Denominator:<br><br>number of adults recognised as being in the last days of life	Exclusion: -	Performance standard: -	Included	N/A	07-DOMAIN 7 OPTIMAL TREATMENT OF SYMPTOMS AND PROVIDING COMFORT	Outcome
352	National Institute for Health and Care Excellence, 2017	Adults in the last days of life who are likely to need symptom control are prescribed anticipatory medicines with individualised indications for use, dosage and route of administration<br><br>Proportion of adults who had their key symptoms controlled in the last days of life	Numerator: -	Denominator: -	Exclusion: -	Performance standard: -	Included	N/A	07-DOMAIN 7 OPTIMAL TREATMENT OF SYMPTOMS AND PROVIDING COMFORT	Outcome
353	National Institute for Health and Care Excellence, 2017	Adults in the last days of life have their hydration status assessed daily, and have a discussion about the risks and benefits of hydration options<br><br>Evidence of local arrangements to ensure that adults in the last days of life have their hydration status assessed daily	Numerator: -	Denominator: -	Exclusion: -	Performance standard: -	Excluded	Conceptualisation and/or Operationalisation	N/A	N/A
354	National Institute for Health and Care Excellence, 2017	Adults in the last days of life have their hydration status assessed daily, and have a discussion about the risks and benefits of hydration options<br><br>Evidence of local arrangements to ensure that adults in the last days of life have their hydration status assessed daily	Numerator: -	Denominator: -	Exclusion: -	Performance standard: -	Excluded	Conceptualisation and/or Operationalisation	N/A	N/A
355	National Institute for Health and Care Excellence, 2017	Adults in the last days of life have their hydration status assessed daily, and have a discussion about the risks and benefits of hydration options<br><br>Proportion of adults recognised as being in the last days of life who have their hydration status assessed daily	Numerator:<br><br>the number in the denominator who have their hydration status assessed daily	Denominator:<br><br>the number of adults recognised as being in the last days of life	Exclusion: -	Performance standard: -	Included	N/A	07-DOMAIN 7 OPTIMAL TREATMENT OF SYMPTOMS AND PROVIDING COMFORT	Process

356	National Institute for Health and Care Excellence, 2017	Adults in the last days of life have their hydration status assessed daily, and have a discussion about the risks and benefits of hydration options<br><br>Proportion of adults recognised as being in the last days of life who have a discussion about the risks and benefits of hydration options	Numerator:<br><br>the number in the denominator whose individual care plan shows that there has been a discussion about the risks and benefits of hydration options	Denominator:<br><br>the number of adults recognised as being in the last days of life	Exclusion: -	Performance standard: -	Included	N/A	07-DOMAIN 7 OPTIMAL TREATMENT OF SYMPTOMS AND PROVIDING COMFORT	Process
357	National Institute for Health and Care Excellence, 2017	Adults in the last days of life have their hydration status assessed daily, and have a discussion about the risks and benefits of hydration options<br><br>Proportion of adults who felt comfortable in the last days of life	Numerator: -	Denominator: -	Exclusion: -	Performance standard: -	Included	N/A	07-DOMAIN 7 OPTIMAL TREATMENT OF SYMPTOMS AND PROVIDING COMFORT	Outcome
358	National Institute for Health and Care Excellence, 2017	Adults in the last days of life have their hydration status assessed daily, and have a discussion about the risks and benefits of hydration options<br><br>Proportion of bereaved people who feel satisfied that the person who has died was supported to drink or receive fluids if they wished	Numerator: -	Denominator: -	Exclusion: -	Performance standard: -	Included	N/A	07-DOMAIN 7 OPTIMAL TREATMENT OF SYMPTOMS AND PROVIDING COMFORT	Outcome
359	Hui, 2015	Integration of Palliative care and oncology programs for advanced cancer patients in hospitals with >= 100 beds<br><br>Presence of palliative care inpatient consultation team	Numerator: -	Denominator: -	Exclusion: -	Performance standard: -	Excluded	Relevance to long-term care settings	N/A	N/A
360	Hui, 2015	Integration of Palliative care and oncology programs for advanced cancer patients in hospitals with >= 100 beds<br><br>Presence of palliative care outpatient clinic	Numerator: -	Denominator: -	Exclusion: -	Performance standard: -	Excluded	Relevance to long-term care settings	N/A	N/A
361	Hui, 2015	Integration of Palliative care and oncology programs for advanced cancer patients in hospitals with >= 100 beds<br><br>Presence of interdisciplinary palliative care team	Numerator: -	Denominator: -	Exclusion: -	Performance standard: -	Excluded	Relevance to long-term care settings	N/A	N/A

362	Hui, 2015	Integration of Palliative care and oncology programs for advanced cancer patients in hospitals with >= 100 beds<br><br>Routine symptom screening in the outpatientoncology clinic	Numerator: -	Denominator: -	Exclusion: -	Performance standard: -	Excluded	Relevance to long-term care settings	N/A	N/A
363	Hui, 2015	Integration of Palliative care and oncology programs for advanced cancer patients in hospitals with >= 100 beds<br><br>Routine documentation of advance care plans in patients with advanced cancer	Numerator: -	Denominator: -	Exclusion: -	Performance standard: -	Excluded	Relevance to long-term care settings	N/A	N/A
364	Hui, 2015	Integration of Palliative care and oncology programs for advanced cancer patients in hospitals with >= 100 beds<br><br>Early referral to palliative care	Numerator: -	Denominator: -	Exclusion: -	Performance standard: -	Excluded	Relevance to long-term care settings	N/A	N/A
365	Hui, 2015	Integration of Palliative care and oncology programs for advanced cancer patients in hospitals with >= 100 beds<br><br>Proportion of outpatients with pain assessed on either of the last two visits before death	Numerator: -	Denominator: -	Exclusion: -	Performance standard: -	Excluded	Relevance to long-term care settings	N/A	N/A
366	Hui, 2015	Integration of Palliative care and oncology programs for advanced cancer patients in hospitals with >= 100 beds<br><br>Proportion of patients with 2 or more emergency room visits in the last 30 days of life (negative indicator)	Numerator: -	Denominator: -	Exclusion: -	Performance standard: -	Included	N/A	06-DOMAIN 6 AVOIDING OVERLY AGGRESSIVE, BURDENSOME OR FUTILE TREATMENT	Process
367	Hui, 2015	Integration of Palliative care and oncology programs for advanced cancer patients in hospitals with >= 100 beds<br><br>Place of death consistent with patient's preference	Numerator: -	Denominator: -	Exclusion: -	Performance standard: -	Excluded	Relevance to long-term care settings	N/A	N/A

368	Hui, 2015	Integration of Palliative care and oncology<br>programs for advanced cancer patients in hospitals with >= 100 beds<br><br>Didactic palliative care curriculum for oncology fellows provided by palliative care teams	Numerator: -	Denominator: -	Exclusion: -	Performance standard: -	Excluded	Relevance to long-term care settings	N/A	N/A
369	Hui, 2015	Integration of Palliative care and oncology<br>programs for advanced cancer patients in hospitals with >= 100 beds<br><br>Continuing medical education in palliative care for attending oncologists	Numerator: -	Denominator: -	Exclusion: -	Performance standard: -	Excluded	Relevance to long-term care settings	N/A	N/A
370	Hui, 2015	Integration of Palliative care and oncology<br>programs for advanced cancer patients in hospitals with >= 100 beds<br><br>Combined palliative care and oncology educational activities for fellows/trainees	Numerator: -	Denominator: -	Exclusion: -	Performance standard: -	Excluded	Relevance to long-term care settings	N/A	N/A
371	Hui, 2015	Integration of Palliative care and oncology<br>programs for advanced cancer patients in hospitals with >= 100 beds<br><br>Oncology fellows have routine rotation in palliative care	Numerator: -	Denominator: -	Exclusion: -	Performance standard: -	Excluded	Relevance to long-term care settings	N/A	N/A
372	Leemans, 2017	Minimal set of Qis for Palliative Care/<br>Physical aspects of care<br><br>Have you or any other professional carer evaluated the<br>patient's symptom burden since the admission or start of<br>palliative care?<br>¨ Yes, with a scale<br>¨ Yes, without a scale<br>¨ No	Numerator:<br><br>number of patients who were subjected to a general symptom assessment on a validated scale	Denominator:<br><br>total number of patients for whom this<br>indicator was measured	Exclusion: -	Performance standard: -	Included	N/A	07-DOMAIN 7 OPTIMAL TREATMENT OF SYMPTOMS<br>AND PROVIDING COMFORT	Process

373	Leemans, 2017	Minimal set of Qis for Palliative Care/<br>Physical aspects of care<br><br>Did you have the feeling that the patient's symptom burden (such as pain, anxiety, shortness of breath . . .) was undercontrol in the last week before death?<br>¨ Yes, totally<br>¨ Yes, largely<br>¨ No, not totally<br>¨ No, totally not<br>¨ I don't know.	Numerator:<br><br>number of patients whose symptom burden was mostly or completely under control in the final week of life	Denominator:<br><br>total number of patients for whom a palliative care service was involved for at least 3 days	Exclusion: -	Performance standard: -	Included	N/A	07-DOMAIN 7 OPTIMAL TREATMENT OF SYMPTOMS AND PROVIDING COMFORT	Outcome
374	Leemans, 2017	Minimal set of Qis for Palliative Care/<br>Physical aspects of care<br><br>Have you or any other professional carer measured the patient's pain since the admission or start of palliative care?<br>¨Yes, with a pain scale<br>¨ Yes, without a pain scale<br>¨ No	Numerator:<br><br>number of patients who were subjected to a pain assessment, with or without pain scale	Denominator:<br><br>total number of patients for whom this indicator was measured	Exclusion: -	Performance standard: -	Included	N/A	07-DOMAIN 7 OPTIMAL TREATMENT OF SYMPTOMS AND PROVIDING COMFORT	Process
375	Leemans, 2017	Minimal set of Qis for Palliative Care/<br>Physical aspects of care<br><br>In your opinion, did the treatment adequately relieve the pain?<br>¨ Yes,<br>¨ No	Numerator:<br><br>number of patients who, after treatment, experienced significant improvement in pain	Denominator:<br><br>total number of patients treated for pain	Exclusion: -	Performance standard: -	Included	N/A	07-DOMAIN 7 OPTIMAL TREATMENT OF SYMPTOMS AND PROVIDING COMFORT	Outcome

376	Leemans, 2017	Minimal set of Qis for Palliative Care/<br>Physical aspects of care<br><br>Was the patient suffering from shortness of breath at the time of admission or when palliative care was started?<br>¨ Yes<br>¨ No<br>Did you or any other professional carer take action to relieve this patient's shortness of breath after admission or after starting palliative care?<br>¨ Yes<br>¨ No<br>How long after the patient was admitted or after palliative care was started was a relief of the shortness of breath achieved?<br>¨ (hours-days)<br>¨ No relief was achieved	Numerator:<br><br>number of patients whose shortness of breath was relieved within 48 hours after admission or starting palliative care	Denominator:<br><br>total number of patients with shortness of breath	Exclusion: -	Performance standard: -	Included	N/A	07-DOMAIN 7 OPTIMAL TREATMENT OF SYMPTOMS AND PROVIDING COMFORT	Outcome
377	Leemans, 2017	Minimal set of Qis for Palliative Care/<br>Psychosocial aspects of care<br><br>Have your professional carers checked how you are feeling?<br>¨ Yes, regularly<br>¨ Yes, once<br>¨ No	Numerator:<br>number of patients who indicated that the caregivers regularly assessed how they were feeling	Denominator:<br>total number of patients for whom this indicator was measured	Exclusion: -	Performance standard: -	Included	N/A	08-DOMAIN 8 PSYCHOSOCIAL AND SPIRITUAL SUPPORT	Process
378	Leemans, 2017	Minimal set of Qis for Palliative Care/<br>Psychosocial aspects of care<br><br>When you consider all aspects of your life—physical, psychological, social, spiritual, existential, and financial—your quality of life over the past 2 days was:<br>(very bad) 0 ——————— 10 (excellent)	Numerator:<br>number of patients with a score of 5 or more on a scale of 0 to 10 for quality of life	Denominator:<br>total number of patients for whom this indicator was measured	Exclusion: -	Performance standard: -	Included	N/A	08-DOMAIN 8 PSYCHOSOCIAL AND SPIRITUAL SUPPORT	Outcome

379	Leemans, 2017	Minimal set of Qis for Palliative Care/<br>Psychosocial aspects of care<br><br>Do your professional carers give you the freedom to plan your day?<br>¨ Never<br>¨ Sometimes<br>¨ Usually<br>¨ Always<br>¨ Not applicable.<br>Can you codecide about your care?<br>¨ Never<br>¨ Sometimes<br>¨ Usually<br>Do your professional carers take your personal wishes into account?<br>¨ None of them do<br>¨ Some of them do<br>¨ Most of them do<br>¨ All of them do	Numerator:<br><br>number of patients who indicated that the caregivers most of the times or always respected their personal wishes and that they could most of the time or always plan their day and decide about the care provided	Denominator:<br><br>total number of patients for whom this indicator was measured	Exclusion: -	Performance standard: -	Included	N/A	08-DOMAIN 8 PSYCHOSOCIAL AND SPIRITUAL SUPPORT	Outcome
380	Leemans, 2017	Minimal set of Qis for Palliative Care/<br>Communication and care planning with patient<br><br>Are you getting enough information about the diagnosis?<br>¨ less than necessary<br>¨ just the right amount<br>¨ more than enough	Numerator:<br>number of patients who received the right amount of information about their diagnosis	Denominator:<br>total number of patients for whom this indicator was measured	Exclusion: -	Performance standard: -	Included	N/A	02-DOMAIN 2 PERSON-CENTRED CARE, COMMUNICATION AND SHARED DECSION MAKING	Outcome
381	Leemans, 2017	Minimal set of Qis for Palliative Care/<br>Communication and care planning with patient<br><br>Are you getting enough information about the course of the disease?<br>¨ less than necessary<br>¨ just the right amount<br>¨ more than enough	Numerator:<br>number of patients who received the right amount of information about the course of the disease	Denominator:<br>total number of patients for whom this indicator was measured	Exclusion: -	Performance standard: -	Included	N/A	02-DOMAIN 2 PERSON-CENTRED CARE, COMMUNICATION AND SHARED DECSION MAKING	Outcome

382	Leemans, 2017	Minimal set of Qis for Palliative Care/<br>Communication and care planning with patient<br><br>Are you getting enough information with regard to end-oflife care?<br>¨ less than necessary<br>¨ just the right amount<br>¨c more than enough	Numerator:<br>number of patients who received the right amount of information on palliative care options	Denominator:<br>total number of patients for whom this indicator was measured	Exclusion: -	Performance standard: -	Included	N/A	02-DOMAIN 2 PERSON-CENTRED CARE, COMMUNICATION AND SHARED DECSION MAKING	Outcome
383	Leemans, 2017	Minimal set of Qis for Palliative Care/<br>Communication and care planning with patient<br><br>Do your professional carers explain things to you understandably?<br>¨ Never<br>¨ Sometimes<br>¨c Usually<br>¨ Always<br><br>Do your professional carers give you conflicting information?<br>¨ Never<br>¨ Sometimes<br>¨ Usually<br>¨ Always	Numerator:<br>number of patients who indicated that they most of the time or always received clear and comprehensible and never or sometimes contradictory information	Denominator:<br>total number of patients for whom this indicator was measured	Exclusion: -	Performance standard: -	Included	N/A	02-DOMAIN 2 PERSON-CENTRED CARE, COMMUNICATION AND SHARED DECSION MAKING	Outcome
384	Leemans, 2017	Minimal set of Qis for Palliative Care/<br>Communication and care planning with patient<br><br>Have you ever talked to the patient about his or her wishes with regard to the care objectives?<br>¨ Yes, once<br>¨ Yes, several times<br>¨ No<br>¨ I don’t know	Numerator:<br>number of patients with whom the physician discussed the care objectives	Denominator:<br>total number of patients for whom this indicator was measured	Exclusion: -	Performance standard: -	Included	N/A	02-DOMAIN 2 PERSON-CENTRED CARE, COMMUNICATION AND SHARED DECSION MAKING	Process

385	Leemans, 2017	Minimal set of Qis for Palliative Care/<br>Communication and care planning with patient<br><br>Have you or another professional carer inquired about the patient's opinion with regard to end-of-life decisions?<br>¨ Yes,<br>¨ No<br>Have you or another professional carer asked the patient's opinion with regard to euthanasia?<br>¨ Yes<br>¨ No	Numerator:<br>number of patients (or representatives) who were asked how they felt about end-of-life decisions and euthanasia	Denominator:<br>total number of patients for whom this<br>indicator was measured	Exclusion: -	Performance standard: -	Included	N/A	02-DOMAIN 2 PERSON-CENTRED CARE, COMMUNICATION AND SHARED DECSION MAKING	Process
386	Leemans, 2017	Minimal set of Qis for Palliative Care/<br>Communication and care planning with patient<br><br>Indicate about which of the following treatment decisions your relative asked the doctor or another professional carer a question.<br>¨ Discontinuing a life-prolonging treatment<br>¨ Increasing pain medication with a potentially life-shortening effect<br>¨ Administering medication with the intention of rendering your relative unconscious (palliative sedation)<br>¨ Administering life-ending drugs on your relative's own explicit request (euthanasia)<br>¨ None of the above decisions<br>Did the professional carers take each of these questions about a treatment decision seriously?<br>¨ Yes<br>¨ No	Numerator:<br>number of patients who got a positive response to (all) their request(s) for a treatment or an end-of-life decision	Denominator:<br>number of patients who requested a treatment or an end-of-life decision	Exclusion: -	Performance standard: -	Included	N/A	02-DOMAIN 2 PERSON-CENTRED CARE, COMMUNICATION AND SHARED DECSION MAKING	Outcome

387	Leemans, 2017	Minimal set of Qis for Palliative Care/<br>Communication and care planning with family<br><br>Did you receive information about your relative's condition?<br>¨ less than needed<br>¨ the right amount<br>¨ more than needed<br>Did you get information about the pros and cons of different treatments?<br>¨ less than necessary<br>¨ just the right amount<br>¨ more than enough	Numerator:<br>number of family carers who received the right amount of information about the patient's condition and treatments	Denominator:<br>total number of family carers for whom this indicator was measured	Exclusion: -	Performance standard: -	Included	N/A	09-DOMAIN 9 FAMILY CARE AND INVOLVEMENT	Outcome
388	Leemans, 2017	Minimal set of Qis for Palliative Care/<br>Communication and care planning with family<br><br>Did you get information about the impending death?<br>c less than necessary<br>c just the right amount<br>c more than enough	Numerator:<br>number of family carers who received the right amount of information about the patient's approaching<br>death	Denominator:<br>total number of family carers for whom this indicator was measured	Exclusion: -	Performance standard: -	Included	N/A	09-DOMAIN 9 FAMILY CARE AND INVOLVEMENT	Outcome
389	Leemans, 2017	Minimal set of Qis for Palliative Care/<br>Communication and care planning with family<br><br>How long after admission or after palliative care was started,<br>did the professional carers and family have a conversation<br>about the patient's care preferences?<br>¨ (hours-days)<br>¨ No conversation has taken place (yet) with the family<br>about the care preferences	Numerator:<br>number of patients about whom a conversation<br>about their care preferences took place between the professional caregivers and family carers in the first week<br>after admission or start of palliative care	Denominator:<br>total number of patients for whom this<br>indicator was measured	Exclusion: -	Performance standard: -	Included	N/A	09-DOMAIN 9 FAMILY CARE AND INVOLVEMENT	Process

390	Leemans, 2017	Minimal set of Qis for Palliative Care/<br>Communication and care planning among professional caregivers<br><br>How often, in the past month, was a formal multidisciplinary consultation meeting held for this patient between professional carers to discuss the care objectives and/or options in terms of palliative care? c once<br>¨ Less than once a week<br>¨ Approximately once a week<br>¨ Almost daily<br>¨ No formal multidisciplinary meetings have taken place (yet)	Numerator:<br><br>number of patients about whom multidisciplinary consultations took place at least once a week about their care objectives	Denominator:<br><br>total number of patients for whom the palliative care service was involved for at least 1 week	Exclusion: -	Performance standard: -	Included	N/A	04-DOMAIN 4 CONTINUITY OF CARE	Process
391	Leemans, 2017	Minimal set of Qis for Palliative Care/Type of care and circumstances surrounding death<br><br>Please indicate on a scale from 0 to 10 how you perceived your relative's death?<br>(restless death) 0 ——————10 (peaceful death)	Numerator:<br>number of patients who scored 5 or more on a scale of 0 to 10 for the quality of death according to family carers	Denominator:<br>total number of patients for whom this indicator was measured	Exclusion: -	Performance standard: -	Included	N/A	07-DOMAIN 7 OPTIMAL TREATMENT OF SYMPTOMS AND PROVIDING COMFORT	Outcome
392	Leemans, 2017	Minimal set of Qis for Palliative Care/Type of care and circumstances surrounding death<br><br>Please indicate on a scale from 0 to 10 how you perceived the patient's death?<br>(restless death) 0 ——————————— 10<br>(peaceful death)	Numerator:<br>number of patients who scored 5 or more on a scale of 0 to 10 for the quality of death according to physicians	Denominator:<br>total number of patients for whom this indicator was measured	Exclusion: -	Performance standard: -	Included	N/A	07-DOMAIN 7 OPTIMAL TREATMENT OF SYMPTOMS AND PROVIDING COMFORT	Outcome
393	Leemans, 2017	Minimal set of Qis for Palliative Care/Type of care and circumstances surrounding death<br><br>When did you start the palliative care for this patient?<br>¨ (dd/mm/yyyy)<br>¨ I don't know.<br>When did this patient start receiving support from the palliative team? (only if the respondent is the family physician)<br>¨ (dd/mm/yy)<br>¨ I don't know	Numerator:<br>number of patients whose palliative care started at least 2 weeks before death	Denominator:<br>total number of patients for whom this indicator was measured	Exclusion: -	Performance standard: -	Included	N/A	07-DOMAIN 7 OPTIMAL TREATMENT OF SYMPTOMS AND PROVIDING COMFORT	Process

394	Leemans, 2017	Minimal set of Qis for Palliative Care/Type of care and circumstances surrounding death<br><br>Was this patient admitted to the emergency department more than once since admission or since the start of the palliative care?<br>¨ Yes<br>¨ No<br>¨ I don't know	Numerator: number of patients admitted more than once to the emergency room since admission or start of palliative care	Denominator: total number of patients for whom this indicator was measured	Exclusion: -	Performance standard: -	Included	N/A	06-DOMAIN 6 AVOIDING OVERLY AGGRESSIVE, BURDENSOME OR FUTILE TREATMENT	Process
395	Leemans, 2017	Minimal set of Qis for Palliative Care/Type of care and circumstances surrounding death<br><br>Was this patient ever taken to intensive care since admission or since the start of the palliative care?<br>¨ Yes<br>¨ No<br>¨ I don't know.	Numerator: number of patients admitted to the intensive care unit since admission or start of palliative care	Denominator: total number of patients for whom this indicator was measured	Exclusion: -	Performance standard: -	Included	N/A	06-DOMAIN 6 AVOIDING OVERLY AGGRESSIVE, BURDENSOME OR FUTILE TREATMENT	Process
396	Leemans, 2017	Minimal set of Qis for Palliative Care/ Coordination and Continuity of care<br><br>Did you have contact with the patient in the last 3 months of his/her life?<br>¨ Yes, by telephone<br>¨ Yes, in person<br>¨ Yes, both by telephone and in person<br>¨ No<br>How often did you see or hear from the patient in those last 3 months?<br>¨ Every day<br>¨ A few times a week<br>¨ Every week<br>¨ Every month<br>¨ Less than once a month<br>¨ I don't know	Numerator: number of patients who were in touch with their family physician on a weekly basis in the last 3 months before death (personally or by telephone)	Denominator: total number of patients for whom this indicator was measured	Exclusion: -	Performance standard: -	Included	N/A	04-DOMAIN 4 CONTINUITY OF CARE	Process

397	Leemans, 2017	Minimal set of Qis for Palliative Care/<br>Coordination and Continuity of care<br><br>Did your GP, the physician, or other professional carers inform you of the patient's care objectives?<br>¨ Yes,<br>¨ No<br>Did your GP, the physician, or other professional carers inform you of the patient's resuscitation status?<br>¨ Yes,<br>¨ No	Numerator:<br>number of patients whose caregivers were given the care objectives and resuscitation status during or after admission or starting palliative care	Denominator:<br>total number of patients for whom this indicator was measured	Exclusion: -	Performance standard: -	Included	N/A	04-DOMAIN 4 CONTINUITY OF CARE	Process
398	Leemans, 2017	Minimal set of Qis for Palliative Care/<br>Coordination and Continuity of care<br><br>Which of the following information was noted in the records within 48 hours after admission or starting palliative care?<br>¨ Patient's prognosis<br>¨ Patient's psychosocial symptoms<br>¨ Patient's functional status<br>¨ Patient's general symptom burden<br>¨ Documentation with regard to the patient's desired care and treatment	Numerator:<br>number of patients for whom the prognosis, psychosocial symptoms, functional status, symptom burden, and documentation about care wishes were entered in the file within 48 hours after admission or starting palliative care	Denominator:<br>total number of patients for whom this indicator was measured	Exclusion: -	Performance standard: -	Included	N/A	04-DOMAIN 4 CONTINUITY OF CARE	Process
399	Leemans, 2017	Minimal set of Qis for Palliative Care/<br>Care for Family<br><br>Did the professional carers talk to you about what it meant to you to care for your sick relative?<br>¨ Yes, regularly<br>¨ Yes, once<br>¨ No	Numerator:<br>number of family carers who indicated that the caregivers regularly talked to them about what it meant to them to care for their ill next of kin	Denominator:<br>total number of family carers who cared for their ill next of kin	Exclusion: -	Performance standard: -	Included	N/A	09-DOMAIN 9 FAMILY CARE AND INVOLVEMENT	Process
400	Leemans, 2017	Minimal set of Qis for Palliative Care/<br>Care for Family<br><br>Did the professional carers ask how you were feeling?<br>¨ Yes, regularly<br>¨ Yes, a few times<br>¨ No	Numerator:<br>number of family carers who indicated that the caregivers regularly asked how they were feeling	Denominator:<br>total number of family carers for whom this indicator was measured	Exclusion: -	Performance standard: -	Included	N/A	09-DOMAIN 9 FAMILY CARE AND INVOLVEMENT	Process

401	Leemans, 2017	Minimal set of Qis for Palliative Care/<br>Care for Family<br><br>Did you feel supported by the professional carers<br>immediately after the death of your relative?<br>¨ Yes<br>¨ No<br>¨ Not applicable.<br>Was there a farewell- or staff appraisal interview that looked back on the care and treatment that was provided?<br>¨ Yes<br>¨ No<br>Were the possibilities of after-care pointed out to you after<br>the death of your relative?<br>¨ Yes<br>¨ No	Numerator:<br>number of family carers who felt they had adequate support after the patient's death (inclusive<br>evaluation meeting) and were informed of the possibilities<br>of after-care.	Denominator:<br>total number of family carers for whom this indicator was measured	Exclusion: -	Performance standard: -	Included	N/A	09-DOMAIN 9 FAMILY CARE AND INVOLVEMENT	Outcome
402	Leemans, 2017	Minimal set of Qis for Palliative Care/<br>Care for Family<br><br>Did you feel that the professional carers gave you all the help<br>and support you needed to care for your relative?<br>¨ Yes, I received all the help I needed<br>¨ Yes, I received help but not enough<br>¨ No, although I tried to get more help<br>¨ No, but I did not ask for more help either<br>¨ I did not need any help	Numerator:<br>number of family carers who were given as much assistance as necessary with the care process	Denominator:<br>total number of family carers who needed<br>assistance with the care process	Exclusion: -	Performance standard: -	Included	N/A	09-DOMAIN 9 FAMILY CARE AND INVOLVEMENT	Outcome
403	Raijmakers, 2012	Presence of dedicated room space for meetings<br>on ICU between clinicians and familiesb	Numerator: -	Denominator: -	Exclusion: -	Performance standard: -	Excluded	Relevance to long-term care<br>settings	N/A	N/A
404	Raijmakers, 2013	More than 90% of all families served by<br>home palliative care services should have received a home visit on the days following patients death to provide support during the mourning processb	Numerator: -	Denominator: -	Exclusion: -	Performance standard: 90%	Included	N/A	09-DOMAIN 9 FAMILY CARE AND INVOLVEMENT	Process
405	Raijmakers, 2014	Fewer than 10% of patients who died<br>from cancer received chemotherapy in the last 14 days of life	Numerator: -	Denominator: -	Exclusion: -	Performance standard: 10%	Excluded	Procedural Relevance	N/A	N/A

406	Raijmakers, 2015	The score for pain control is 0-1 in at least 75% of all patients during the last week of lifeb	Numerator: -	Denominator: -	Exclusion: -	Performance standard: 75%	Included	N/A	07-DOMAIN 7 OPTIMAL TREATMENT OF SYMPTOMS AND PROVIDING COMFORT	Outcome
407	Raijmakers, 2016	The score for communication from professional to patient and family is 0-1 in at least 75% of all patients during the final week of life	Numerator: -	Denominator: -	Exclusion: -	Performance standard: 75%	Included	N/A	02-DOMAIN 2 PERSON-CENTRED CARE, COMMUNICATION AND SHARED DECSION MAKING	Outcome
408	Raijmakers, 2017	The global score for gastrointestinal symptoms does not increase over the initial score during the final week of life in at least 75% of the patientsb	Numerator: -	Denominator: -	Exclusion: -	Performance standard: 75%	Included	N/A	07-DOMAIN 7 OPTIMAL TREATMENT OF SYMPTOMS AND PROVIDING COMFORT	Outcome
409	Raijmakers, 2018	The global score for pain does not increase over the initial score during the final week of life in at least 75% of the patientsb	Numerator: -	Denominator: -	Exclusion: -	Performance standard: 75%	Included	N/A	07-DOMAIN 7 OPTIMAL TREATMENT OF SYMPTOMS AND PROVIDING COMFORT	Outcome
410	Sanders, 2017	Disclosure of the Cause of Dementia<br><br>Percentage of patients with a diagnosis of a qualifying dementing disorder or disease whose diagnosis has been disclosed to them and, if available, their primary caregiver	Numerator: Patients and patient/caregiver dyads with a diagnosis* of a qualifying dementing disorder or disease who have been told (1) that they have dementia and (2) what disease is responsible	Denominator: All patients with a diagnosis of a qualifying dementing disorder or disease	Exclusions: Diagnosis previously disclosed (i.e., patient or caregiver provides diagnosis to provider, previous disclosure documented in medical record, or patient or caregiver states they were previously informed.)<br>• Disclosure offered, but patient declines information on their diagnosis<br>• Patient does not have caregiver	Performance standard: -	Included	N/A	02-DOMAIN 2 PERSON-CENTRED CARE, COMMUNICATION AND SHARED DECSION MAKING	Process

411	Sanders, 2017	Education and Support of Caregivers for Patients with Dementia<br><br>Percentage of patients with dementia whose caregiver(s) were provided with education on dementia disease management and health behavior changes AND were referred to additional resources for support in the last 12 months	Numerator:<br>Patients with dementia whose caregiver(s) were provided with education on dementia disease management and health behavior changes AND were referred to additional resources** for support in the last 12 months.	Denominator:<br>All patients with dementia	Exclusions:<br>Patient does not have a caregiver.<br>• Caregiver is trained and certified in dementia care.<br>• Patient/caregiver dyad referred to appropriate resources and connection to those resources confirmed.	Performance standard: -	Included	N/A	02-DOMAIN 2 PERSON-CENTRED CARE, COMMUNICATION AND SHARED DECSION MAKING	Process
412	Sanders, 2017	Functional Status Assessment for Patients with Dementia<br><br>Percentage of patients with dementia for whom an assessment of functional status was performed at least once in the last 12 months	Numerator:<br>Patients with dementia for whom an assessment of functional status was performed at least once in the last 12 months.	Denominator:<br>All patients with dementia	Exclusions:<br>None	Performance standard: -	Included	N/A	07-DOMAIN 7 OPTIMAL TREATMENT OF SYMPTOMS AND PROVIDING COMFORT	Process
413	Sanders, 2017	Screening and Management of Behavioral and Psychiatric Symptoms Associated with Dementia<br><br>Percentage of patients with dementia for whom there was a documented screening for behavioral and psychiatric symptoms, including depression, and for whom, if screening was positive, there was also documentation of recommendations for management in the last 12 months	Numerator:<br>Patients with dementia for whom there was a documented screening for behavioral and psychiatric symptoms, including depression in the last 12 months and for whom, if screening was positive, there was also documentation of recommendations for management in the last 12 months.	Denominator:<br>All patients with dementia	Exclusions:<br>None	Performance standard: -	Included	N/A	07-DOMAIN 7 OPTIMAL TREATMENT OF SYMPTOMS AND PROVIDING COMFORT	Process
414	Sanders, 2017	Safety Concern Screening and Follow-Up for Patients with Dementia<br><br>Percentage of patients with dementia or their caregiver(s) for whom there was a documented safety screening in two domains of risk: dangerousness to self or others and environmental risks; and if screening was positive in the last 12 months, there was documentation of mitigation recommendations, including but not limited to referral to other resources.	Numerator:<br>Patients with dementia or their caregiver(s) for whom there was a documented safety screening in two domains of risk: dangerousness to self or others and environmental risks; and if screening was positive in the last 12 months, there was documentation of mitigation recommendations, including but not limited to referral to other resources.	Denominator:<br>All patients with dementia	Exclusions:<br>Patient unable to communicate and informant not available.	Performance standard: -	Included	N/A	08-DOMAIN 8 PSYCHOSOCIAL AND SPIRITUAL SUPPORT	Process

415	Sanders, 2017	Driving Screening and Follow-Up for Patients with Dementia<br><br>Percentage of patients with dementia for whom there was a documented screening for driving risks and for whom, if screening positive, there was also documentation they were informed of alternatives to driving in the last 12 months.	Numerator:<br>Patients with dementia for whom there was a documented screening for driving risks and for whom, if screening positive, there was also documentation they were informed of alternatives to driving in the last 12 months.	Denominator:<br>All patients with dementia	Exclusions:<br>None	Performance standard: -	Included	N/A	09-DOMAIN 9 FAMILY CARE AND INVOLVEMENT	Process
416	Sanders, 2017	Advance Care Planning and Palliative Care<br>Counseling for Patients with Dementia<br><br>Percentage of patients with dementia who<br>1) have an advance care plan or surrogate decisions maker documented in the medical record or documentation in the medical record that an advance care plan was discussed but the patient did not wish or was not able to name a surrogate decision maker or provide an advance care plan<br>AND<br>Patients with dementia or their surrogate decision maker who<br>2) received comprehensive counseling regarding ongoing palliation & symptom management, and end of life decisions within two years of initial diagnosis or assumption of care.	Numerator:<br>Patients with dementia who<br>1) have an advance care plan or surrogate decisions maker documented in the medical record or documentation in the medical record that an advance care plan was discussed but the patient did not wish or was not able to name a surrogate decision maker or provide an advance care plan<br>AND<br>Patients with dementia or their surrogate decision maker who<br>2) received comprehensive counseling regarding ongoing palliation & symptom management, and end of life decisions within two years of initial diagnosis or assumption of care.	Denominator:<br>All patients with dementia	Exclusions:<br>Patients who at time of intake had advanced dementia for whom there was no record of advance care planning or surrogate decision-maker prior to their admission.	Performance standard: -	Included	N/A	03-DOMAIN 3 SETTING CARE GOALS<br>AND ADVANCE PLANNING	Process
417	Sanders, 2017	Pain Assessment and Follow-Up for Patients with Dementia<br><br>Percentage of patients with dementia who underwent documented screening for pain symptoms at every visit and if screening was positive also had documentation of a follow-up plan	Numerator:<br>Patients with dementia who underwent documented screening for pain symptoms at every visit and if screening was positive also had documentation of a follow-up plan	Denominator:<br>All patients with dementia	Exclusions:<br>None	Performance standard: -	Included	N/A	07-DOMAIN 7 OPTIMAL TREATMENT OF SYMPTOMS<br>AND PROVIDING COMFORT	Process

418	Sanders, 2017	Pharmacological Treatment of Dementia<br><br>Percentage of patients with dementia or their caregivers with whom available guideline-appropriate pharmacological treatment options and nonpharmacological behavior and lifestyle modifications were discussed at least once in the last12-month period	Numerator:<br>Patients with dementia or their caregivers with whom available guideline-appropriate pharmacological treatment options and nonpharmacological behavior and lifestyle modifications were discussed at least once in the last12-month period	Denominator:<br>All patients with dementia	Exclusions:<br>None	Performance standard: -	Included	N/A	07-DOMAIN 7 OPTIMAL TREATMENT OF SYMPTOMS AND PROVIDING COMFORT	Process
419	Schnitker, 2015	Management of older persons with cognitive impairment in EDs (general)<br><br>The ED has a policy outlining the management of older people with cognitive impairment during the ED episode of care	Numerator:<br>The number of EDs with policies, procedures, or protocols encompassing the management of older persons with cognitive impairment in the ED, including the identification of special care needs (e.g., delirium risk, ADL support), notifying of support person, and the involvement of support person in care planning.	Denominator:<br>The number of EDs that desire to identify their performance regarding structure quality of care for the older person with cognitive impairment.	Exclusions: -	Performance standard: -	Included	N/A	07-DOMAIN 7 OPTIMAL TREATMENT OF SYMPTOMS AND PROVIDING COMFORT	Structure

420	Schnitker, 2015	Management of (family) carers of older people with cognitive impairment<br><br>The ED has a policy outlining issues relevant to carers of older people with cognitive impairment, encompassing the need to include the carer in the ED episode of care.	Numerator: The number of EDs with policies, procedures or protocols encompassing the management of the (family) carers of older people with cognitive impairment.	Denominator: The number of EDs that desire to identify their performance regarding structure quality of care for the older person with cognitive impairment.	Exclusions: -	Performance standard: -	Included	N/A	09-DOMAIN 9 FAMILY CARE AND INVOLVEMENT	Structure
421	Schnitker, 2015	Assessment and management of behavioral symptoms<br><br>The ED has a policy outlining the assessment and management of behavioral symptoms, with specific reference to older people with cognitive impairment.	Numerator: The number of EDs with policies, procedures, or protocols encompassing the assessment and management of behavioral symptoms, with a specific reference to older people with cognitive impairment	Denominator: The number of EDs that desire to identify their performance regarding structure quality of care for the older person with cognitive impairment.	Exclusions: -	Performance standard: -	Included	N/A	07-DOMAIN 7 OPTIMAL TREATMENT OF SYMPTOMS AND PROVIDING COMFORT	Structure
422	Schnitker, 2015	Delirium prevention<br><br>The ED has a policy outlining delirium prevention strategies, including the assessment of patients' delirium risk factors.	Numerator: The number of EDs with a policy, procedure, or protocols encompassing delirium prevention strategies, including the assessment of patients' delirium risk factors.	Denominator: The number of EDs that desire to identify their performance regarding structure quality of care for the older person with cognitive impairment.	Exclusions: -	Performance standard: -	Included	N/A	07-DOMAIN 7 OPTIMAL TREATMENT OF SYMPTOMS AND PROVIDING COMFORT	Structure

423	Schnitker, 2015	Pain assessment and management in older ED patients with cognitive impairment<br><br>The ED has a policy outlining pain assessment and management for older people with cognitive impairment.	Numerator:<br>The number of EDs sites that have policies, procedures, or protocols encompassing pain assessment (e.g., observational methods) in older people with cognitive impairment.	Denominator:<br>The number of EDs that desire to identify their performance regarding structure quality of care for older persons with cognitive impairment.	Exclusions: -	Performance standard: -	Included	N/A	07-DOMAIN 7 OPTIMAL TREATMENT OF SYMPTOMS AND PROVIDING COMFORT	Structure
424	Schnitker, 2015	Cognitive screening<br><br>Proportion of older people who received cognitive screening in the ED.	Numerator:<br>The number of older ED patients who were screened for cognitive impairment during their ED episode of care	Denominator:<br>The number of older ED patients	Exclusions:<br>- Older ED patients assigned triage level 1 (resuscitation)	Performance standard: -	Included	N/A	07-DOMAIN 7 OPTIMAL TREATMENT OF SYMPTOMS AND PROVIDING COMFORT	Process
425	Schnitker, 2015	Delirium screening<br><br>Proportion of older people who received a screen for delirium in the ED.	Numerator:<br>The number of older ED patients who were screened for delirium during their ED episode of care	Denominator<br>The number of older ED patients	Exclusions:<br>Older ED patients assigned triage level 1 (resuscitation)	Performance standard: -	Included	N/A	07-DOMAIN 7 OPTIMAL TREATMENT OF SYMPTOMS AND PROVIDING COMFORT	Process
426	Schnitker, 2015	Delirium risk assessment<br><br>Proportion of older people in the ED who were assessed for the risk of delirium.	Numerator:<br>The number of older ED patients who were assessed for delirium risk during their ED episode of care	Denominator:<br>The number of older ED patients	Exclusions:<br>- Older ED patients assigned triage level 1 (resuscitation)<br>- Older ED patients diagnosed with delirium during their ED episode of care	Performance standard: -	Included	N/A	07-DOMAIN 7 OPTIMAL TREATMENT OF SYMPTOMS AND PROVIDING COMFORT	Process
427	Schnitker, 2015	Evaluation of acute change in mental status<br><br>Proportion of older people with cognitive impairment in the ED whose cognition was assessed for an acute change.	Numerator:<br>The number of older ED patients with cognitive impairment whose pre-morbid mental status was assessed for acute change	Denominator:<br>The number of older ED patients with cognitive impairment (identified by using a formal cognitive screening tool)	Exclusions:<br>- Older ED patients assigned triage level 1 (resuscitation)	Performance standard: -	Included	N/A	07-DOMAIN 7 OPTIMAL TREATMENT OF SYMPTOMS AND PROVIDING COMFORT	Process

428	Schnitker, 2015	Delirium etiology<br><br>Proportion of older people with suspected or definite diagnosis of delirium whose altered mental state was attributed to a potential	Numerator:<br>The number of older ED patients who have a suspected or definite diagnosis of delirium where the ED provider documented an attempt to attribute the altered mental state to a potential etiology	Denominator:<br>The number of older ED patients who have a suspected or definite diagnosis of delirium (identified by using medical record review).	Exclusions: -	Performance standard: -	Included	N/A	07-DOMAIN 7 OPTIMAL TREATMENT OF SYMPTOMS AND PROVIDING COMFORT	Process
429	Schnitker, 2015	Attendance of nominated support person<br><br>Proportion of older people with cognitive impairment in the ED where the ED provider ensured those close to the patient were notified.	Numerator:<br>The number of older ED patients with cognitive impairment whose nominated support person was notified during the ED episode of care.	Denominator:<br>The number of older ED patients with cognitive impairment (identified by using a formal cognitive screening tool).	Exclusions: -	Performance standard: -	Included	N/A	09-DOMAIN 9 FAMILY CARE AND INVOLVEMENT	Process
430	Schnitker, 2015	Collateral history<br><br>Proportion of older people with cognitive impairment in the ED where the ED provider obtained collateral history	Numerator:<br>The number of older ED patients with cognitive impairment where ED staff obtained collateral history during the ED episode of care.	Denominator:<br>The number of older ED patients with cognitive impairment (identified by using a formal cognitive screening tool).	Exclusions:	Performance standard: -	Included	N/A	09-DOMAIN 9 FAMILY CARE AND INVOLVEMENT	Process
431	Schnitker, 2015	Proxy notification<br><br>Proportion of older people with cognitive impairment in the ED where the ED provider involved the patient's nominated or legally authorized decision-maker in the care plan.	Numerator:<br>The number of older ED patients with cognitive impairment whose nominated or legally authorized decision-maker was involved in care planning during the ED episode of care.	Denominator:<br>The number of older ED patients with cognitive impairment (identified by using a formal cognitive screening tool).	Exclusions:	Performance standard: -	Included	N/A	09-DOMAIN 9 FAMILY CARE AND INVOLVEMENT	Process
432	Schnitker, 2015	Pain assessment<br><br>Proportion of older people with cognitive impairment who were assessed for pain in the ED.	Numerator:<br>Number of older ED patients with cognitive impairment who were assessed for pain using (besides self-report) other resources for pain identification, such as observational approaches, behavioral assessment or proxy report of pain, during their ED episode of care.	Denominator:<br>The number of older ED patients with cognitive impairment (identified by using a formal cognitive screening tool).	Exclusions:	Performance standard: -	Included	N/A	07-DOMAIN 7 OPTIMAL TREATMENT OF SYMPTOMS AND PROVIDING COMFORT	Process

433	Schnitker, 2015	Post-discharge follow-up<br><br>Proportion of older people with previously unrecognized cognitive impairment, which was not delirium, and an ED end status of discharged where the ED provider documented a referral for further cognitive evaluation.	Numerator:<br>The number of older persons who have an abnormal mental status that has not been previously recognized or diagnosed by another health care provider AND have no change in mental status from baseline AND have an ED service episode end status of discharged where the ED provider documents a referral for outpatient evaluation of the cognitive impairment.	Denominator:<br>The number of older persons who have abnormal mental status that has not been previously recognized or diagnosed by another health care provider AND have no change in mental status from baseline AND have an ED service episode end status of discharged.	Exclusions:	Performance standard: -	Included	N/A	07-DOMAIN 7 OPTIMAL TREATMENT OF SYMPTOMS AND PROVIDING COMFORT	Process
434	Schnitker, 2015	ED length of stay<br><br>Proportion of older people with cognitive impairment with an ED length of stay greater than eight hours.	Numerator:<br>The number of older ED patients with cognitive impairment whose ED episode of care exceeded eight hours.	Denominator:<br>The number of older ED patients with cognitive impairment (identified by using a formal cognitive screening tool).	Exclusions:	Performance standard: -	Included	N/A	06-DOMAIN 6 AVOIDING OVERLY AGGRESSIVE, BURDENSOME OR FUTILE TREATMENT	Outcome
435	Sinuff, 2015	EoL Communication and Decision Making/<br>Advance Care Planning<br><br>Before hospitalization, the patient discussed his/her preferences for using or not using life-sustaining treatments with their substitute decision maker (SDM)	Numerator: -	Denominator: -	Exclusion: -	Performance standard: -	Excluded	Conceptualisation and/or Operationalisation	N/A	N/A
436	Sinuff, 2015	EoL Communication and Decision Making/<br>Advance Care Planning<br><br>Before hospitalization, the doctor talked to the patient and/or a family member about a poor prognosis or indicated in some way that the patient has a limited time left to live	Numerator: -	Denominator: -	Exclusion: -	Performance standard: -	Excluded	Conceptualisation and/or Operationalisation	N/A	N/A
437	Sinuff, 2015	EoL Communication and Decision Making/<br>Advance Care Planning<br><br>Before hospitalization, the patient and/or a family member discussed their preferences for using or not using medically appropriate life-sustaining treatments with their family doctor or other doctor	Numerator: -	Denominator: -	Exclusion: -	Performance standard: -	Excluded	Conceptualisation and/or Operationalisation	N/A	N/A

438	Sinuff, 2015	EoL Communication and Decision Making/ Advance Care Planning<br><br>Before hospitalization, the patient discussed his/her preferences for using or not using medically appropriate life-sustaining treatments with other family members	Numerator: -	Denominator: -	Exclusion: -	Performance standard: -	Excluded	Conceptualisation and/or Operationalisation	N/A	N/A
439	Sinuff, 2015	EoL Communication and Decision Making/ Advance Care Planning<br><br>The patient has formally designated, in writing, someone who they trust to be their SDM concerning medical treatment decisions in the event they are not able to do so (using appropriate legal documentation depending on jurisdiction). In case of power of attorney, it should be related to health care.	Numerator: -	Denominator: -	Exclusion: -	Performance standard: -	Excluded	Conceptualisation and/or Operationalisation	N/A	N/A
440	Sinuff, 2015	EoL Communication and Decision Making/ Advance Care Planning<br><br>Before hospitalization, a member of the health care team offered to arrange a time when the patient and his/her family could meet with the doctor to discuss the use of medically appropriate lifesustaining treatments they would want, or not want, in the event the patient's physical health deteriorates.	Numerator: -	Denominator: -	Exclusion: -	Performance standard: -	Excluded	Conceptualisation and/or Operationalisation	N/A	N/A
441	Sinuff, 2015	EoL Communication and Decision Making/ Advance Care Planning<br><br>The patient has an advance directive or living will or has indicated in some other way (verbal, video, and so on) the medical treatments they would want (or not want) in the event they are unable to communicate for themselves as a result of a life-threatening health problem	Numerator: -	Denominator: -	Exclusion: -	Performance standard: -	Excluded	Conceptualisation and/or Operationalisation	N/A	N/A

442	Sinuff, 2015	EoL Communication and Decision Making/ Advance Care Planning<br><br>Before hospitalization, the patient and/or a family member discussed their preferences for using or not using medically appropriate life-sustaining treatments with other health care professionals (i.e., nurse, social worker, and spiritual carer)	Numerator: -	Denominator: -	Exclusion: -	Performance standard: -	Excluded	Conceptualisation and/or Operationalisation	N/A	N/A
443	Sinuff, 2015	EoL Communication and Decision Making/ Goals of care/scope of treatment discussion<br><br>Since admission, a member of the health care team has talked to the patient and/or SDM about a poor prognosis or indicated in some way that the patient has a limited time left to live	Numerator: -	Denominator: -	Exclusion: -	Performance standard: -	Excluded	Conceptualisation and/or Operationalisation	N/A	N/A
444	Sinuff, 2015	EoL Communication and Decision Making/ Goals of care/scope of treatment discussion<br><br>Since admission, a member of the health care team has talked to the patient and/or SDM about the outcomes, benefits, and burdens (or risks) of life-sustaining medical treatments	Numerator: -	Denominator: -	Exclusion: -	Performance standard: -	Excluded	Conceptualisation and/or Operationalisation	N/A	N/A
445	Sinuff, 2015	EoL Communication and Decision Making/ Goals of care/scope of treatment discussion<br><br>Since admission, a member of the health care team has talked to the patient and/or substitute decision maker about outcomes, benefits, and burdens of focusing on comfort care as the goal of the patient's treatment (e.g., palliative care or treating symptoms like pain without trying to cure or control their underlying illness)	Numerator: -	Denominator: -	Exclusion: -	Performance standard: -	Excluded	Conceptualisation and/or Operationalisation	N/A	N/A

446	Sinuff, 2015	EoL Communication and Decision Making/<br>Goals of care/scope of treatment discussion<br><br>Since the patient's admission, a member of the health care team has offered to arrange a time when the<br>patient/SDM and/or their family can meet with the doctor to discuss the treatment options and<br>plans	Numerator: -	Denominator: -	Exclusion: -	Performance standard: -	Excluded	Conceptualisation and/or Operationalisation	N/A	N/A
447	Sinuff, 2015	EoL Communication and Decision Making/<br>Goals of care/scope of treatment discussion<br><br>Since the patient's admission, a member of the health care team has asked if the patient (or SDM if patient is incapable) had prior discussions or has written documents about the use of life-sustaining<br>treatments	Numerator: -	Denominator: -	Exclusion: -	Performance standard: -	Excluded	Conceptualisation and/or Operationalisation	N/A	N/A
448	Sinuff, 2015	EoL Communication and Decision Making/<br>Goals of care/scope of treatment discussion<br><br>Since the patient's admission, a member of the health care team has asked the patient/SDM and/or their family what is important to them as they consider health care decisions at this stage of the patient's life (i.e., values, spiritual beliefs, and other practices)	Numerator: -	Denominator: -	Exclusion: -	Performance standard: -	Excluded	Conceptualisation and/or Operationalisation	N/A	N/A
449	Sinuff, 2015	EoL Communication and Decision Making/<br>Goals of care/scope of treatment discussion<br><br>Since admission, a member of the health care team has given the patient the opportunity to express their fears or discuss what concerns them	Numerator: -	Denominator: -	Exclusion: -	Performance standard: -	Excluded	Conceptualisation and/or Operationalisation	N/A	N/A

450	Sinuff, 2015	EoL Communication and Decision Making/<br>Goals of care/scope of treatment discussion<br><br>Since admission, a member of the health care team has asked the patient and/or their family if they had<br>any questions or needed things clarified regarding the patient's overall goals of care	Numerator: -	Denominator: -	Exclusion: -	Performance standard: -	Excluded	Conceptualisation and/or Operationalisation	N/A	N/A
451	Sinuff, 2015	EoL Communication and Decision Making/<br>Goals of care/scope of treatment discussion<br><br>Since admission, a member of the health care team has asked the patient what treatments they prefer to<br>have or not have if they develop a life-threatening illness	Numerator: -	Denominator: -	Exclusion: -	Performance standard: -	Excluded	Conceptualisation and/or Operationalisation	N/A	N/A
452	Sinuff, 2015	EoL Communication and Decision Making/<br>Goals of care/scope of treatment discussion<br><br>Since admission, the patient has been informed that they may change their minds regarding their decisions around goals of care	Numerator: -	Denominator: -	Exclusion: -	Performance standard: -	Excluded	Conceptualisation and/or Operationalisation	N/A	N/A
453	Sinuff, 2015	EoL Communication and Decision Making/<br>Goals of care/scope of treatment discussion<br><br>Since admission, the patient and family have been offered an opportunity to discuss with members of<br>the health care team issues around capacity and consent with regard to advance care planning (ACP); specifically what actions would take place in the possible event of losing capacity to consent to care	Numerator: -	Denominator: -	Exclusion: -	Performance standard: -	Excluded	Conceptualisation and/or Operationalisation	N/A	N/A
454	Sinuff, 2015	EoL Communication and Decision Making/<br>Goals of care/scope of treatment discussion<br><br>Since admission, the patient and family have been offered support from the allied health care team	Numerator: -	Denominator: -	Exclusion: -	Performance standard: -	Excluded	Conceptualisation and/or Operationalisation	N/A	N/A

		(e.g., spiritual care, social work, and clinical nurse specialist) as needed.								
455	Sinuff, 2015	EoL Communication and Decision Making/<br>Goals of care/scope of treatment discussion<br><br>Since admission, a member of the health care team provided the patient and/or their family with information about GOCD to look at before conversations with the doctor	Numerator: -	Denominator: -	Exclusion: -	Performance standard: -	Excluded	Conceptualisation and/or Operationalisation	N/A	N/A
456	Sinuff, 2015	EoL Communication and Decision Making/<br>Documentation<br><br>Documentation of a Goals of Care is present in the medical record.	Numerator: -	Denominator: -	Exclusion: -	Performance standard: -	Excluded	Conceptualisation and/or Operationalisation	N/A	N/A
457	Sinuff, 2015	EoL Communication and Decision Making/<br>Documentation<br><br>The Goals of Care present in the medical record is consistent with the patient's stated preferences	Numerator: -	Denominator: -	Exclusion: -	Performance standard: -	Excluded	Conceptualisation and/or Operationalisation	N/A	N/A
458	Sinuff, 2015	EoL Communication and Decision Making/<br>Documentation<br><br>If the hospital uses a standardized folder or other strategy to locate ACP/Goals of Care documents in the medical record, these are present in the medical record	Numerator: -	Denominator: -	Exclusion: -	Performance standard: -	Excluded	Conceptualisation and/or Operationalisation	N/A	N/A
459	Sinuff, 2015	EoL Communication and Decision Making/<br>Documentation<br><br>Documentation of the outcomes of ACP conversations (including any prior expressed wishes, diaries, and power of attorney documents) is present in the patient's medical record	Numerator: -	Denominator: -	Exclusion: -	Performance standard: -	Excluded	Conceptualisation and/or Operationalisation	N/A	N/A

460	Sinuff, 2015	EoL Communication and Decision Making/<br>Documentation<br><br>Since admission, a member of the health care team has helped the patient and/or their family access legal documents to communicate the patient's ACPs	Numerator: -	Denominator: -	Exclusion: -	Performance standard: -	Excluded	Conceptualisation and/or Operationalisation	N/A	N/A
461	Sinuff, 2015	EoL Communication and Decision Making/<br>Organisational system aspects<br><br>A mechanism is in place to enable access to the most current ACP/GOCD documents with the patient<br>in other settings within the health care system (i.e., electronic medical record and paper files)	Numerator: -	Denominator: -	Exclusion: -	Performance standard: -	Excluded	Conceptualisation and/or Operationalisation	N/A	N/A
462	Sinuff, 2015	EoL Communication and Decision Making/<br>Organisational system aspects<br><br>Institution uses a standardized folder or other strategy to locate ACP/Goals of Care documents in the<br>medical record	Numerator: -	Denominator: -	Exclusion: -	Performance standard: -	Excluded	Conceptualisation and/or Operationalisation	N/A	N/A
463	Sinuff, 2015	EoL Communication and Decision Making/<br>Organisational system aspects<br><br>The Institution ensures that clinical staff has access to the necessary professional development resources<br>to ensure ACP facilitation skills can be attained or maintained	Numerator: -	Denominator: -	Exclusion: -	Performance standard: -	Excluded	Conceptualisation and/or Operationalisation	N/A	N/A
464	Sinuff, 2015	EoL Communication and Decision Making/<br>Organisational system aspects<br><br>The Institution has documented ACP policies and/or procedures	Numerator: -	Denominator: -	Exclusion: -	Performance standard: -	Excluded	Conceptualisation and/or Operationalisation	N/A	N/A
465	Sinuff, 2015	EoL Communication and Decision Making/<br>Organisational system aspects<br><br>The Institution has policies and procedures in place so that ''high-risk'' (as defined by the institution) patients participate in ACP/GOCD processes	Numerator: -	Denominator: -	Exclusion: -	Performance standard: -	Excluded	Conceptualisation and/or Operationalisation	N/A	N/A

466	Sinuff, 2015	EoL Communication and Decision Making/<br>Organisational system aspects<br><br>The Institution has a continuous quality improvement initiative that audits and provides feedback to teams on specific ACP elements outlined in previous items	Numerator: -	Denominator: -	Exclusion: -	Performance standard: -	Excluded	Conceptualisation and/or Operationalisation	N/A	N/A
467	Sinuff, 2015	EoL Communication and Decision Making/<br>Organisational system aspects<br><br>Institution management evaluates ACP knowledge and skills amongst relevant staff	Numerator: -	Denominator: -	Exclusion: -	Performance standard: -	Excluded	Conceptualisation and/or Operationalisation	N/A	N/A
468	Sinuff, 2015	EoL Communication and Decision Making/<br>Organisational system aspects<br><br>The Institution has a process in place whereby patients with a specific disease, such as advanced chronic obstructive pulmonary disease, cancer, neurological disease, or heart failure are offered diseasespecific advance directives	Numerator: -	Denominator: -	Exclusion: -	Performance standard: -	Excluded	Conceptualisation and/or Operationalisation	N/A	N/A
469	van Riet Paap, 2014	Access to palliative care<br>Access and availability<br><br>A specialist palliative care team* is available 24/7	Numerator: -	Denominator: -	Exclusion: -	Performance standard: -	Excluded	Conceptualisation and/or Operationalisation	N/A	N/A
470	van Riet Paap, 2014	Access to palliative care<br>Access and availability<br><br>Specialist palliative care* advice is available 24/7 to professionals delivering palliative care.	Numerator: -	Denominator: -	Exclusion: -	Performance standard: -	Excluded	Conceptualisation and/or Operationalisation	N/A	N/A
471	van Riet Paap, 2014	Access to palliative care<br>Access and availability<br><br>Bereaved relatives and/or professionals involved in care of a person in need of palliative care are offered support during the bereavement process if they need or wish to have support.	Numerator: -	Denominator: -	Exclusion: -	Performance standard: -	Excluded	Conceptualisation and/or Operationalisation	N/A	N/A
472	van Riet Paap, 2014	Access to palliative care<br>Out of hours care<br><br>Opioids are accessible and available for persons in need of palliative care 24/7	Numerator: -	Denominator: -	Exclusion: -	Performance standard: -	Excluded	Conceptualisation and/or Operationalisation	N/A	N/A

473	van Riet Paap, 2014	Access to palliative care<br>Out of hours care<br><br>Co-analgesics* for symptom control are available to treat persons in need of palliative care 24/7	Numerator: -	Denominator: -	Exclusion: -	Performance standard: -	Excluded	Conceptualisation and/or Operationalisation	N/A	N/A
474	van Riet Paap, 2014	Access to care<br>Continuity of care<br><br>An (electronic) file of a person in need of palliative care is accessible to professionals in charge of the person 24/7	Numerator: -	Denominator: -	Exclusion: -	Performance standard: -	Excluded	Conceptualisation and/or Operationalisation	N/A	N/A
475	van Riet Paap, 2014	Access to care<br>Continuity of care<br><br>At each transition between care settings, comprehensive information (including care goals and care plan) of a person in need of palliative care is be transferred to the professional(s) in charge in the next setting	Numerator: -	Denominator: -	Exclusion: -	Performance standard: -	Excluded	Conceptualisation and/or Operationalisation	N/A	N/A
476	van Riet Paap, 2014	Access to care<br>Continuity of care<br><br>The professional in charge of the person is informed before a person in need of palliative care is discharged home or sent to the next setting	Numerator: -	Denominator: -	Exclusion: -	Performance standard: -	Excluded	Conceptualisation and/or Operationalisation	N/A	N/A
477	van Riet Paap, 2014	Access to care<br>Continuity of care<br><br>Persons in need of palliative care have an assigned contact person who maintains regular contact with the person and their families, and ensures coordinated delivery of health and social care	Numerator: -	Denominator: -	Exclusion: -	Performance standard: -	Excluded	Conceptualisation and/or Operationalisation	N/A	N/A
478	van Riet Paap, 2014	Infrastructure<br><br>Specialised equipment (e.g. anti decubitus mattresses, suction equipment, stoma care, oxygen delivery, drug administration pumps, hospital beds, etc.) is available to persons in need of palliative care	Numerator: -	Denominator: -	Exclusion: -	Performance standard: -	Excluded	Conceptualisation and/or Operationalisation	N/A	N/A
479	van Riet Paap, 2014	Infrastructure<br><br>Single bedrooms are available for persons who are dying and who wish to have one	Numerator: -	Denominator: -	Exclusion: -	Performance standard: -	Excluded	Conceptualisation and/or Operationalisation	N/A	N/A

480	van Riet Paap, 2014	Infrastructure<br><br>Family members and friends are able to visit the dying person without restrictions of visiting hours	Numerator: -	Denominator: -	Exclusion: -	Performance standard: -	Excluded	Conceptualisation and/or Operationalisation	N/A	N/A
481	van Riet Paap, 2014	Infrastructure<br><br>There are facilities for relatives to stay overnight with their dying relative	Numerator: -	Denominator: -	Exclusion: -	Performance standard: -	Excluded	Conceptualisation and/or Operationalisation	N/A	N/A
482	van Riet Paap, 2014	Infrastructure<br><br>There is a private area for saying goodbye to the deceased, nearby or on the ward/unit where the person died	Numerator: -	Denominator: -	Exclusion: -	Performance standard: -	Excluded	Conceptualisation and/or Operationalisation	N/A	N/A
483	van Riet Paap, 2014	Assessment tools<br><br>There is a regular assessment of pain and other symptoms using a validated instrument	Numerator: -	Denominator: -	Exclusion: -	Performance standard: -	Excluded	Conceptualisation and/or Operationalisation	N/A	N/A
484	van Riet Paap, 2014	Personnel<br>Team<br><br>The multidisciplinary team that delivers palliative care services consists of at least:<br>a) a physician and nurse;<br>b) and has access to one or more of the following professionals: physiotherapist, psychologist, occupational therapist, social worker, chaplain, dietician.	Numerator: -	Denominator: -	Exclusion: -	Performance standard: -	Excluded	Conceptualisation and/or Operationalisation	N/A	N/A
485	van Riet Paap, 2014	Personnel<br>Team<br><br>There is a weekly multidisciplinary meeting with at least the physician and nurse in charge of the person in need of palliative care to review treatment and care plans	Numerator: -	Denominator: -	Exclusion: -	Performance standard: -	Excluded	Conceptualisation and/or Operationalisation	N/A	N/A

486	van Riet Paap, 2014	Personnel<br>Sharing information<br><br>The file of the person in need of palliative care contains documentation of a discussion with the person or representative (if the person lacks capacity e.g. is unable to communicate) about:<br>a) medical condition;<br>b) goals for treatment;<br>c) the physical*, psychosocial and spiritual needs of the person and family caregiver;<br>d) an advance directive or advanced care plan;<br>e) end-of-life decisions*;<br>f) the intention to return home or to another facility from the place where the person is currently staying.	Numerator: -	Denominator: -	Exclusion: -	Performance standard: -	Excluded	Conceptualisation and/or Operationalisation	N/A	N/A
487	van Riet Paap, 2014	Documentation of clinical data<br>Clinical records<br><br>The file of the person in need of palliative care contains a medication list that is accessible to the professionals caring for the person	Numerator: -	Denominator: -	Exclusion: -	Performance standard: -	Excluded	Conceptualisation and/or Operationalisation	N/A	N/A
488	van Riet Paap, 2014	Documentation of clinical data<br>Timely documentation<br><br>Within 48 hours of admission to the service, the file of the person in need of palliative care contains documentation of the initial assessment of:<br>a) pain and other symptoms, using a validated instrument*;<br>b) psychosocial and spiritual needs;<br>c) persons preferences, wishes and needs;<br>d) capacity to be involved in the decision making process.	Numerator: -	Denominator: -	Exclusion: -	Performance standard: -	Excluded	Conceptualisation and/or Operationalisation	N/A	N/A
489	van Riet Paap, 2014	Quality<br><br>Family and caregiver experiences of the palliative care service are assessed/evaluated/recorded	Numerator: -	Denominator: -	Exclusion: -	Performance standard: -	Excluded	Conceptualisation and/or Operationalisation	N/A	N/A

490	van Riet Paap, 2014	Quality<br><br>An end-of-life care pathway (such as the Liverpool Care Pathway) was used for the last 3 days of life of a person in need of palliative care	Numerator: -	Denominator: -	Exclusion: -	Performance standard: -	Excluded	Conceptualisation and/or Operationalisation	N/A	N/A
491	van Riet Paap, 2014	Education<br><br>All professionals that deliver palliative care services receive accredited training in palliative care, appropriate to their discipline	Numerator: -	Denominator: -	Exclusion: -	Performance standard: -	Excluded	Conceptualisation and/or Operationalisation	N/A	N/A
492	Walling, 2017	Information and Care planning<br><br>IF a patient has advanced end-stage liver disease and hepatocellular carcinoma (HCC) with a BCLC<br>Stage D and is not a candidate for transplant, THEN the patient should be offered palliative and/or hospice services or a reason why not should be documented BECAUSE survival is likely less than<br>6 months and hospice provides additional support for patients and caregivers/family	Numerator: -	Denominator: -	Exclusion: -	Performance standard: -	Excluded	Procedural Relevance	N/A	N/A
493	Walling, 2017	Information and Care planning<br><br>IF a patient has advanced end-stage liver disease and is diagnosed with hepatorenal syndrome and is<br>not a liver transplant candidate, THEN the patient should be offered palliative and/or hospice services BECAUSE these patients have a poor prognosis and would benefit from the additional supportive services	Numerator: -	Denominator: -	Exclusion: -	Performance standard: -	Excluded	Procedural Relevance	N/A	N/A
494	Walling, 2017	Information and Care planning<br><br>IF a patient with advanced end-stage liver disease dies an expected death, THEN there should be<br>evidence that he or she has been offered palliative care or hospice within 6 months before death BECAUSE palliative care teams can improve a range of patient and caregiver outcomes	Numerator: -	Denominator: -	Exclusion: -	Performance standard: -	Excluded	Procedural Relevance	N/A	N/A

495	Walling, 2017	Information and Care planning<br><br>IF a patient has advanced end-stage liver disease and is being considered for transplant, THEN the patient should be offered advance care planning within 3 months of consideration of transplant or a reason why such a discussion did not occur BECAUSE effective communication about goals of care is important to ensure that patient preferences are followed during the course of their illness	Numerator: -	Denominator: -	Exclusion: -	Performance standard: -	Excluded	Procedural Relevance	N/A	N/A
496	Walling, 2017	Information and Care planning<br><br>IF a patient is newly diagnosed with advanced end-stage liver disease and is not considered for transplant, THEN the patient should be offered advance care planning within 3 months or a reason why such a discussion did not occur BECAUSE effective communication about goals of care is important to ensure that patient preferences are followed during the course of their illness	Numerator: -	Denominator: -	Exclusion: -	Performance standard: -	Excluded	Procedural Relevance	N/A	N/A
497	Walling, 2017	Information and Care planning<br><br>IF a patient with advanced end-stage liver disease dies an expected death, THEN there should be documentation of advance care planning in the medical record within 6 months before death BECAUSE a patient's goals and values should guide treatment and life-sustaining care decisions	Numerator: -	Denominator: -	Exclusion: -	Performance standard: -	Excluded	Procedural Relevance	N/A	N/A

498	Walling, 2017	Information and Care planning<br><br>IF a patient with advanced end-stage liver disease is admitted to a hospital or nursing home, THEN within 48 h of admission the medical record should contain the name of the patient's surrogate decision maker, or documentation of a discussion to identify/search for a surrogate decision maker BECAUSE patient's values and preferences should guide life-sustaining care and patients with end-stage liver disease often cannot make decisions for themselves	Numerator: -	Denominator: -	Exclusion: -	Performance standard: -	Excluded	Procedural Relevance	N/A	N/A
499	Walling, 2017	Information and Care planning<br><br>IF a patient with advanced end-stage liver disease is newly diagnosed with hepatic encephalopathy<br>and does not have advance care planning previously documented, THEN the medical record should document advance care planning or lack of ability to do so within 1 month BECAUSE patients<br>with a history of hepatic encephalopathy are at higher risk of lacking capacity to make decisions for<br>themselves and care should be guided by their goals and preferences	Numerator: -	Denominator: -	Exclusion: -	Performance standard: -	Excluded	Procedural Relevance	N/A	N/A
500	Walling, 2017	Information and Care planning<br><br>IF a patient with advanced end-stage liver disease is admitted to the ICU and survives 48 h, THEN within 48 h, the medical record should document that the patient's preferences for care have been considered or an attempt was made to identify them BECAUSE patients with end-stage liver disease are at a high risk of requiring life-sustaining treatment decisions	Numerator: -	Denominator: -	Exclusion: -	Performance standard: -	Excluded	Procedural Relevance	N/A	N/A

501	Walling, 2017	Information and Care planning<br><br>IF a patient with advanced end-stage liver disease requires mechanical ventilation for greater than<br>48 h, THEN within 48 h of the initiation of mechanical ventilation, the medical record should document the goals of care and the patient's preference for mechanical ventilation or why this information is not available BECAUSE patients' values and preferences should guide lifesustaining<br>care	Numerator: -	Denominator: -	Exclusion: -	Performance standard: -	Excluded	Procedural Relevance	N/A	N/A
502	Walling, 2017	Information and Care planning<br><br>IF a patient with advanced end-stage liver disease has orders in the hospital or nursing home to withhold or withdraw a life-sustaining treatment (e.g., DNR, no tube feeding, no hospital transfer),<br>THEN these treatment preferences should be followed BECAUSE patients' values and<br>preferences should guide end-of-life care	Numerator: -	Denominator: -	Exclusion: -	Performance standard: -	Excluded	Procedural Relevance	N/A	N/A
503	Walling, 2017	Information and Care planning<br><br>IF a patient with advanced end-stage liver disease who is not a candidate for transplant has the following: new hemodialysis or placement of a pacemaker or ICD, THEN within one month prior<br>to the procedure, the medical record should document the goals of care and the patient's preference<br>for the intervention, BECAUSE a patient's treatment should reflect his or her goals of care	Numerator: -	Denominator: -	Exclusion: -	Performance standard: -	Excluded	Procedural Relevance	N/A	N/A

504	Walling, 2017	Information and Care planning<br><br>IF an inpatient with advanced end-stage liver disease has an advance directive or DNR at the time of discharge and the patient receives care in a second venue, THEN the advance directive and/or DNR should be present in the medical record of the second venue or documentation should acknowledge its existence, its contents, and the reason it is not in the medical record, BECAUSE an advance directive can guide care only if its existence is recognized and its contents are known	Numerator: -	Denominator: -	Exclusion: -	Performance standard: -	Excluded	Procedural Relevance	N/A	N/A
505	Walling, 2017	Supportive care<br><br>IF a patient with advanced end-stage liver disease is receiving acetaminophen, THEN the total daily dose should not exceed 3 grams or a reason why the patient is receiving more should be documented BECAUSE acetaminophen-induced hepatotoxicity may have serious consequences in cirrhosis	Numerator: -	Denominator: -	Exclusion: -	Performance standard: -	Excluded	Procedural Relevance	N/A	N/A
506	Walling, 2017	Supportive care<br><br>IF a patient has advanced end-stage liver disease, THEN the patient should not be prescribed NSAIDS BECAUSE of the increased risk of renal toxicity and bleeding	Numerator: -	Denominator: -	Exclusion: -	Performance standard: -	Excluded	Procedural Relevance	N/A	N/A
507	Walling, 2017	Supportive care<br><br>IF a patient with advanced end-stage liver disease who is not enrolled in hospice presents with ascites with moderate to severe symptoms (pain, shortness of breath, or non-reducible hernia), THEN the patient should be offered a paracentesis within 48 h or a reason why not should be documented BECAUSE treatment can improve patients' symptoms	Numerator: -	Denominator: -	Exclusion: -	Performance standard: -	Excluded	Procedural Relevance	N/A	N/A

508	Walling, 2017	Supportive care<br><br>IF a patient with advanced end-stage liver disease is not expected to survive and a mechanical ventilator is withdrawn or withheld, THEN the medical chart should document whether the patient has dyspnea BECAUSE dyspnea can be controlled in the setting of comfort care when mechanical ventilation is withdrawn or withheld	Numerator: -	Denominator: -	Exclusion: -	Performance standard: -	Excluded	Procedural Relevance	N/A	N/A
509	Walling, 2017	Supportive care<br><br>IF a patient with advanced end-stage liver disease is not expected to survive and a mechanical ventilator is withdrawn or withheld, THEN the medical chart should document whether the patient should receive (or have orders available for) dyspnea management BECAUSE dyspnea can be controlled in the setting of comfort care when mechanical ventilation is withdrawn or withheld	Numerator: -	Denominator: -	Exclusion: -	Performance standard: -	Excluded	Procedural Relevance	N/A	N/A
510	Walling, 2017	Supportive care<br><br>IF a patient with advanced end-stage liver disease who had dyspnea in the last 3 days of life died an expected death, THEN the chart should document dyspnea care and follow-up BECAUSE dyspnea can be effectively treated with pharmacological agents	Numerator: -	Denominator: -	Exclusion: -	Performance standard: -	Excluded	Procedural Relevance	N/A	N/A
511	Woitha, 2012	Definition of a palliative care service<br>All the services below are part of a comprehensive palliative care service:Palliaitve day care, Palliaitve home care support team, Hospice beds, Palliaitve hospital support team, Inpatiente palliative care hospital beds, Palliaitve care outpatient clinic, Bereavement support	Numerator: -	Denominator: -	Exclusion: -	Performance standard: -	Excluded	Conceptualisation and/or Operationalisation	N/A	N/A
512	Woitha, 2012	Definition of a palliative care service<br>All the services below are part of a comprehensive palliative care service: Palliative day care	Numerator: -	Denominator: -	Exclusion: -	Performance standard: -	Excluded	Conceptualisation and/or Operationalisation	N/A	N/A

513	Woitha, 2012	Definition of a palliative care service All the services below are part of a comprehensive palliative care service: Palliative home care support team	Numerator: -	Denominator: -	Exclusion: -	Performance standard: -	Excluded	Conceptualisation and/or Operationalisation	N/A	N/A
514	Woitha, 2012	Definition of a palliative care service All the services below are part of a comprehensive palliative care service: Hospice beds	Numerator: -	Denominator: -	Exclusion: -	Performance standard: -	Excluded	Conceptualisation and/or Operationalisation	N/A	N/A
515	Woitha, 2012	Definition of a palliative care service All the services below are part of a comprehensive palliative care service: Palliative hospital support team	Numerator: -	Denominator: -	Exclusion: -	Performance standard: -	Excluded	Conceptualisation and/or Operationalisation	N/A	N/A
516	Woitha, 2012	Definition of a palliative care service All the services below are part of a comprehensive palliative care service: Inpatient palliative care hospital beds (e.g. palliative care unit)	Numerator: -	Denominator: -	Exclusion: -	Performance standard: -	Excluded	Conceptualisation and/or Operationalisation	N/A	N/A
517	Woitha, 2012	Definition of a palliative care service All the services below are part of a comprehensive palliative care service: Palliative care outpatient clinic	Numerator: -	Denominator: -	Exclusion: -	Performance standard: -	Excluded	Conceptualisation and/or Operationalisation	N/A	N/A
518	Woitha, 2012	Definition of a palliative care service All the services below are part of a comprehensive palliative care service: Bereavement support	Numerator: -	Denominator: -	Exclusion: -	Performance standard: -	Excluded	Conceptualisation and/or Operationalisation	N/A	N/A
519	Woitha, 2012	Access to palliative care Access and availability (All settings) A palliative care team is available at the request of the treating professional/team in all of the following settings: Day care, at home, Hospital, Hospice, Nursing home, Outpatient clinic, Day care	Numerator: -	Denominator: -	Exclusion: -	Performance standard: -	Excluded	Conceptualisation and/or Operationalisation	N/A	N/A
520	Woitha, 2012	Access to palliative care Access and availability (All settings) A palliative care team is available at the request of the treating professional/team in all of the following settings: Day care (excluding palliative day care)	Numerator: -	Denominator: -	Exclusion: -	Performance standard: -	Excluded	Conceptualisation and/or Operationalisation	N/A	N/A

521	Woitha, 2012	Access to palliative care Access and availability (All settings) A palliative care team is available at the request of the treating professional/team in all of the following settings: At home (or home replacing institution s.a mental institution, prison)	Numerator: -	Denominator: -	Exclusion: -	Performance standard: -	Excluded	Conceptualisation and/or Operationalisation	N/A	N/A
522	Woitha, 2012	Access to palliative care Access and availability (All settings) A palliative care team is available at the request of the treating professional/team in all of the following settings: Hospital	Numerator: -	Denominator: -	Exclusion: -	Performance standard: -	Excluded	Conceptualisation and/or Operationalisation	N/A	N/A
523	Woitha, 2012	Access to palliative care Access and availability (All settings) A palliative care team is available at the request of the treating professional/team in all of the following settings: Hospice	Numerator: -	Denominator: -	Exclusion: -	Performance standard: -	Excluded	Conceptualisation and/or Operationalisation	N/A	N/A
524	Woitha, 2012	Access to palliative care Access and availability (All settings) A palliative care team is available at the request of the treating professional/team in all of the following settings: Care home	Numerator: -	Denominator: -	Exclusion: -	Performance standard: -	Excluded	Conceptualisation and/or Operationalisation	N/A	N/A
525	Woitha, 2012	Access to palliative care Access and availability (All settings) A palliative care team is available at the request of the treating professional/team in all of the following settings: Outpatient clinic (excluding palliative care outpatient clinic)	Numerator: -	Denominator: -	Exclusion: -	Performance standard: -	Excluded	Conceptualisation and/or Operationalisation	N/A	N/A
526	Woitha, 2012	Access to palliative care Access and availability (All settings) For every professional/team specialised palliative care advice is available 24 hours a day, 7 days a week	Numerator: -	Denominator: -	Exclusion: -	Performance standard: -	Excluded	Conceptualisation and/or Operationalisation	N/A	N/A
527	Woitha, 2012	Access to palliative care Access and availability (All settings) Patients in need of palliative care and their families have access to palliative care facilities: Throughout the entire duration of their disease	Numerator: -	Denominator: -	Exclusion: -	Performance standard: -	Excluded	Conceptualisation and/or Operationalisation	N/A	N/A
528	Woitha, 2012	Access to palliative care Access and availability (All settings) Patients in need of palliative care and their families have access to palliative care facilities: With no extra financial consequences for the patient	Numerator: -	Denominator: -	Exclusion: -	Performance standard: -	Excluded	Conceptualisation and/or Operationalisation	N/A	N/A

529	Woitha, 2012	Access to palliative care Access and availability (All settings) Patients receiving palliative care have access to diagnostic investigations (e.g. X-rays, blood samples) regardless of their setting	Numerator: -	Denominator: -	Exclusion: -	Performance standard: -	Excluded	Conceptualisation and/or Operationalisation	N/A	N/A
530	Woitha, 2012	Access to palliative care Primary care (Home, Nursing home) Palliative care is available for the patient and their family by:Phone	Numerator: -	Denominator: -	Exclusion: -	Performance standard: -	Excluded	Conceptualisation and/or Operationalisation	N/A	N/A
531	Woitha, 2012	Access to palliative care Primary care (Home, Nursing home) Palliative care is available for the patient and their family by:Visiting the patient	Numerator: -	Denominator: -	Exclusion: -	Performance standard: -	Excluded	Conceptualisation and/or Operationalisation	N/A	N/A
532	Woitha, 2012	Access to palliative care Primary care (Home, Nursing home) Palliative care is available for the patient and their family by:Bringing the patient to the service	Numerator: -	Denominator: -	Exclusion: -	Performance standard: -	Excluded	Conceptualisation and/or Operationalisation	N/A	N/A
533	Woitha, 2012	Access to palliative care Primary care (Home, Nursing home) For a palliative patient in a crisis , the following can be arranged within 24 hours: Admission	Numerator: -	Denominator: -	Exclusion: -	Performance standard: -	Excluded	Conceptualisation and/or Operationalisation	N/A	N/A
534	Woitha, 2012	Access to palliative care Primary care (Home, Nursing home) For a palliative patient in a crisis , the following can be arranged within 24 hours: An urgent discharge to patients home	Numerator: -	Denominator: -	Exclusion: -	Performance standard: -	Excluded	Conceptualisation and/or Operationalisation	N/A	N/A
535	Woitha, 2012	Access to palliative care Primary care (Home, Nursing home) For a palliative patient in a crisis , the following can be arranged within 24 hours: Transfer to another setting of care	Numerator: -	Denominator: -	Exclusion: -	Performance standard: -	Excluded	Conceptualisation and/or Operationalisation	N/A	N/A
536	Woitha, 2012	Access to palliative care Out of hours (All settings) A member of a palliative care team is available 24 hours a day, 7 days a week: For palliative care consultation by phone	Numerator: -	Denominator: -	Exclusion: -	Performance standard: -	Excluded	Conceptualisation and/or Operationalisation	N/A	N/A
537	Woitha, 2012	Access to palliative care Out of hours (All settings) A member of a palliative care team is available 24 hours a day, 7 days a week: To provide bedside care in a crisis	Numerator: -	Denominator: -	Exclusion: -	Performance standard: -	Excluded	Conceptualisation and/or Operationalisation	N/A	N/A

538	Woitha, 2012	Access to palliative care Out of hours (All settings) The following treatments are available for a palliative patient 24 hours a day, 7 days a week: Opioids and other controlled drugs	Numerator: -	Denominator: -	Exclusion: -	Performance standard: -	Excluded	Conceptualisation and/or Operationalisation	N/A	N/A
539	Woitha, 2012	Access to palliative care Out of hours (All settings) The following treatments are available for a palliative patient 24 hours a day, 7 days a week: Anticipatory medication for the dying patient	Numerator: -	Denominator: -	Exclusion: -	Performance standard: -	Excluded	Conceptualisation and/or Operationalisation	N/A	N/A
540	Woitha, 2012	Access to palliative care Out of hours (All settings) The following treatments are available for a palliative patient 24 hours a day, 7 days a week: Syringe drivers	Numerator: -	Denominator: -	Exclusion: -	Performance standard: -	Excluded	Conceptualisation and/or Operationalisation	N/A	N/A
541	Woitha, 2012	Access to palliative care Continuity of care (All settings) There is a procedure for exchange of clinical information across caregivers, disciplines and settings	Numerator: -	Denominator: -	Exclusion: -	Performance standard: -	Excluded	Conceptualisation and/or Operationalisation	N/A	N/A
542	Woitha, 2012	Access to palliative care Continuity of care (All settings) Before discharge/ transfer/ admission there is information transfer to the caregivers in the next setting regarding care and treatment	Numerator: -	Denominator: -	Exclusion: -	Performance standard: -	Excluded	Conceptualisation and/or Operationalisation	N/A	N/A
543	Woitha, 2012	Access to palliative care Continuity of care (All settings) There is a professional caregiver per individual palliative patient nominated as responsible 'key worker' who coordinates care	Numerator: -	Denominator: -	Exclusion: -	Performance standard: -	Excluded	Conceptualisation and/or Operationalisation	N/A	N/A
544	Woitha, 2012	Access to palliative care Continuity of care (All settings) The responsible 'key worker' pays special attention to continuity of care within and across settings	Numerator: -	Denominator: -	Exclusion: -	Performance standard: -	Excluded	Conceptualisation and/or Operationalisation	N/A	N/A
545	Woitha, 2012	Access to palliative care Inpatient setting (Hospital, Palliative care unit, Hospice) General practitioners (GP's) are routinely called when a patient is being discharged home or transferred to another setting	Numerator: -	Denominator: -	Exclusion: -	Performance standard: -	Excluded	Conceptualisation and/or Operationalisation	N/A	N/A
546	Woitha, 2012	Access to palliative care Inpatient setting (Hospital, Palliative care unit, Hospice) The discharge/ transfer letter of palliative care patients contains a multidimensional diagnosis,	Numerator: -	Denominator: -	Exclusion: -	Performance standard: -	Excluded	Conceptualisation and/or Operationalisation	N/A	N/A

		prognosis and treatment plan (see indicator 48 Clinical record)								
547	Woitha, 2012	Access to palliative care<br>Primary care The primary care out-of-hours service has handover forms (written or -electronic) with clinical information of all palliative care patients in the terminal phase at home	Numerator: -	Denominator: -	Exclusion: -	Performance standard: -	Excluded	Conceptualisation and/or Operationalisation	N/A	N/A
548	Woitha, 2012	Infrastructure All settings<br>Infrastructure<br>Specialist equipment (e.g. anti decubitus mattresses, aspiration material, stoma care, oxygen delivery, special drug administration pumps, hospital beds, etc) is available for the nursing care of palliative care patients in each specific setting	Numerator: -	Denominator: -	Exclusion: -	Performance standard: -	Excluded	Conceptualisation and/or Operationalisation	N/A	N/A
549	Woitha, 2012	Infrastructure All settings<br>Infrastructure<br>There is a dedicated room where multidisciplinary team meetings within one setting takes place	Numerator: -	Denominator: -	Exclusion: -	Performance standard: -	Excluded	Conceptualisation and/or Operationalisation	N/A	N/A
550	Woitha, 2012	Infrastructure All settings<br>Infrastructure<br>There are dedicated facilities for multidisciplinary communications across settings: A dedicated room for meetings	Numerator: -	Denominator: -	Exclusion: -	Performance standard: -	Excluded	Conceptualisation and/or Operationalisation	N/A	N/A
551	Woitha, 2012	Infrastructure All settings<br>Infrastructure<br>There are dedicated facilities for multidisciplinary communications across settings: Facilities for video or telephone conferences	Numerator: -	Denominator: -	Exclusion: -	Performance standard: -	Excluded	Conceptualisation and/or Operationalisation	N/A	N/A
552	Woitha, 2012	Infrastructure All settings<br>Information<br>about care There is an up to date directory of local caregivers and organisations that can have a role in palliative care	Numerator: -	Denominator: -	Exclusion: -	Performance standard: -	Excluded	Conceptualisation and/or Operationalisation	N/A	N/A
553	Woitha, 2012	Infrastructure All settings<br>Information about care<br>There are dedicated information about the palliative care service: A website	Numerator: -	Denominator: -	Exclusion: -	Performance standard: -	Excluded	Conceptualisation and/or Operationalisation	N/A	N/A
554	Woitha, 2012	Infrastructure All settings<br>Information about care<br>There are dedicated information	Numerator: -	Denominator: -	Exclusion: -	Performance standard: -	Excluded	Conceptualisation and/or Operationalisation	N/A	N/A

		about the palliative care service: Leaflets or brochures								
555	Woitha, 2012	Infrastructure All settings Information about care Patient information should be available in relevant foreign languages	Numerator: -	Denominator: -	Exclusion: -	Performance standard: -	Excluded	Conceptualisation and/or Operationalisation	N/A	N/A
556	Woitha, 2012	Infrastructure All settings Information about care Appropriately trained translators should be available if professional caregivers and patient or family members do not speak the same language	Numerator: -	Denominator: -	Exclusion: -	Performance standard: -	Excluded	Conceptualisation and/or Operationalisation	N/A	N/A
557	Woitha, 2012	Infrastructure All settings Information about care There is a computerised medical record , to which all professional caregivers involved in the care of palliative care patients have access: Within one setting	Numerator: -	Denominator: -	Exclusion: -	Performance standard: -	Excluded	Conceptualisation and/or Operationalisation	N/A	N/A
558	Woitha, 2012	Infrastructure All settings It systems There is a computerised medical record , to which all professional caregivers involved in the care of palliative care patients have access: Across different settings	Numerator: -	Denominator: -	Exclusion: -	Performance standard: -	Excluded	Conceptualisation and/or Operationalisation	N/A	N/A
559	Woitha, 2012	Infrastructure Inpatient setting (Hospital, Palliative care unit, Hospice, Nursing home) Consultations with the patient and/or family / informal caregivers are done in an environment where privacy is guaranteed (e.g. there is a dedicated room)	Numerator: -	Denominator: -	Exclusion: -	Performance standard: -	Excluded	Conceptualisation and/or Operationalisation	N/A	N/A
560	Woitha, 2012	Infrastructure Inpatient setting (Hospital, Palliative care unit, Hospice, Nursing home) Dying patients are able to have a single bedroom if they want to	Numerator: -	Denominator: -	Exclusion: -	Performance standard: -	Excluded	Conceptualisation and/or Operationalisation	N/A	N/A
561	Woitha, 2012	Infrastructure Inpatient setting (Hospital, Palliative care unit, Hospice, Nursing home) There are facilities for a relative to stay overnight	Numerator: -	Denominator: -	Exclusion: -	Performance standard: -	Excluded	Conceptualisation and/or Operationalisation	N/A	N/A
562	Woitha, 2012	Infrastructure Inpatient setting (Hospital, Palliative care unit, Hospice, Nursing home) Family members and friends are able to visit the dying patient without restrictions of visiting hours	Numerator: -	Denominator: -	Exclusion: -	Performance standard: -	Excluded	Conceptualisation and/or Operationalisation	N/A	N/A

563	Woitha, 2012	Infrastructure Inpatient setting (Hospital, Palliative care unit, Hospice, Nursing home)<br>There is a private place (e.g. dedicated room) for saying goodbye to the deceased	Numerator: -	Denominator: -	Exclusion: -	Performance standard: -	Excluded	Conceptualisation and/or Operationalisation	N/A	N/A
564	Woitha, 2012	Infrastructure Home care<br>For a palliative care patient staying at home there is the possibility, if needed, to provide someone (a volunteer or professional) to stay overnight if needed	Numerator: -	Denominator: -	Exclusion: -	Performance standard: -	Excluded	Conceptualisation and/or Operationalisation	N/A	N/A
565	Woitha, 2012	Assessment tools<br>There is a holistic assessment of palliative care needs of patients and their family caregivers (e.g. SPARC)	Numerator: -	Denominator: -	Exclusion: -	Performance standard: -	Excluded	Conceptualisation and/or Operationalisation	N/A	N/A
566	Woitha, 2012	Assessment tools<br>There is an assessment of pain and other symptoms using a validated instrument	Numerator: -	Denominator: -	Exclusion: -	Performance standard: -	Excluded	Conceptualisation and/or Operationalisation	N/A	N/A
567	Woitha, 2012	Personnel palliative care services Staff<br>The multidisciplinary team that provides palliative care consists of at least one of the following disciplines: Physician	Numerator: -	Denominator: -	Exclusion: -	Performance standard: -	Excluded	Conceptualisation and/or Operationalisation	N/A	N/A
568	Woitha, 2012	Personnel palliative care services Staff<br>The multidisciplinary team that provides palliative care consists of at least one of the following disciplines:Nurse	Numerator: -	Denominator: -	Exclusion: -	Performance standard: -	Excluded	Conceptualisation and/or Operationalisation	N/A	N/A
569	Woitha, 2012	Personnel palliative care services Staff<br>The multidisciplinary team that provides palliative care consists of at least one of the following disciplines:Spiritual/ religious caregiver	Numerator: -	Denominator: -	Exclusion: -	Performance standard: -	Excluded	Conceptualisation and/or Operationalisation	N/A	N/A
570	Woitha, 2012	Personnel palliative care services Staff<br>The multidisciplinary team that provides palliative care consists of at least one of the following disciplines: Psychologist/Psychiatrist	Numerator: -	Denominator: -	Exclusion: -	Performance standard: -	Excluded	Conceptualisation and/or Operationalisation	N/A	N/A
571	Woitha, 2012	Personnel palliative care services Staff<br>The multidisciplinary team that provides palliative care consists of at least one of the following disciplines: Social worker	Numerator: -	Denominator: -	Exclusion: -	Performance standard: -	Excluded	Conceptualisation and/or Operationalisation	N/A	N/A

572	Woitha, 2012	Personnel palliative care services Staff The multidisciplinary team that provides palliative care consists of at least one of the following disciplines: Physiotherapist	Numerator: -	Denominator: -	Exclusion: -	Performance standard: -	Excluded	Conceptualisation and/or Operationalisation	N/A	N/A
573	Woitha, 2012	Personnel palliative care services Staff The multidisciplinary team that provides palliative care consists of at least one of the following disciplines: Occupational therapist	Numerator: -	Denominator: -	Exclusion: -	Performance standard: -	Excluded	Conceptualisation and/or Operationalisation	N/A	N/A
574	Woitha, 2012	Personnel palliative care services Staff The multidisciplinary team that provides palliative care consists of at least one of the following disciplines: Dietitian	Numerator: -	Denominator: -	Exclusion: -	Performance standard: -	Excluded	Conceptualisation and/or Operationalisation	N/A	N/A
575	Woitha, 2012	Personnel palliative care services Staff The multidisciplinary team that provides palliative care consists of at least one of the following disciplines: Bereavement counselor	Numerator: -	Denominator: -	Exclusion: -	Performance standard: -	Excluded	Conceptualisation and/or Operationalisation	N/A	N/A
576	Woitha, 2012	Personnel palliative care services Staff The multidisciplinary team that provides palliative care consists of at least one of the following disciplines: Pharmacist	Numerator: -	Denominator: -	Exclusion: -	Performance standard: -	Excluded	Conceptualisation and/or Operationalisation	N/A	N/A
577	Woitha, 2012	Personnel palliative care services Staff New staff receives a standardised induction training	Numerator: -	Denominator: -	Exclusion: -	Performance standard: -	Excluded	Conceptualisation and/or Operationalisation	N/A	N/A
578	Woitha, 2012	Personnel palliative care services Staff All team members have certified (accredited?) training in palliative care, appropriate to their discipline	Numerator: -	Denominator: -	Exclusion: -	Performance standard: -	Excluded	Conceptualisation and/or Operationalisation	N/A	N/A
579	Woitha, 2012	Personnel palliative care services Staff All volunteers have training in palliative care.	Numerator: -	Denominator: -	Exclusion: -	Performance standard: -	Excluded	Conceptualisation and/or Operationalisation	N/A	N/A
580	Woitha, 2012	Personnel palliative care services Support Systems All team members have an annual appraisal	Numerator: -	Denominator: -	Exclusion: -	Performance standard: -	Excluded	Conceptualisation and/or Operationalisation	N/A	N/A
581	Woitha, 2012	Personnel palliative care services Support Systems All team members who professionally deal with loss have access to a program for care for the carers	Numerator: -	Denominator: -	Exclusion: -	Performance standard: -	Excluded	Conceptualisation and/or Operationalisation	N/A	N/A

582	Woitha, 2012	Personnel palliative care services Support Systems Satisfaction with working in the team is assessed (e.g. Team Climate Inventory)	Numerator: -	Denominator: -	Exclusion: -	Performance standard: -	Excluded	Conceptualisation and/or Operationalisation	N/A	N/A
583	Woitha, 2012	Personnel palliative care services Organisation of care Palliative care services work in conjunction with the referring professional/ team	Numerator: -	Denominator: -	Exclusion: -	Performance standard: -	Excluded	Conceptualisation and/or Operationalisation	N/A	N/A
584	Woitha, 2012	Personnel palliative care services Organisation of care There is a regular interdisciplinary/ multi-professional meeting to discuss palliative care patients: daily meetings to discuss day-to-day management of palliative care patients	Numerator: -	Denominator: -	Exclusion: -	Performance standard: -	Excluded	Conceptualisation and/or Operationalisation	N/A	N/A
585	Woitha, 2012	Personnel palliative care services Organisation of care There is a regular interdisciplinary/ multi-professional meeting to discuss palliative care patients:weekly (inter- and multidisciplinary) meeting to review palliative care patients referrals and care plans	Numerator: -	Denominator: -	Exclusion: -	Performance standard: -	Excluded	Conceptualisation and/or Operationalisation	N/A	N/A
586	Woitha, 2012	Personnel palliative care services Information sharing All relevant team members are informed about patients who have died	Numerator: -	Denominator: -	Exclusion: -	Performance standard: -	Excluded	Conceptualisation and/or Operationalisation	N/A	N/A
587	Woitha, 2012	Documentation of clinical data Clinical record (All settings) For patients receiving palliative care a structured palliative care clinical record is used	Numerator: -	Denominator: -	Exclusion: -	Performance standard: -	Excluded	Conceptualisation and/or Operationalisation	N/A	N/A
588	Woitha, 2012	Documentation of clinical data Clinical record (All settings) The palliative care clinical record contains evidence of documentation of the following items: Clinical summary	Numerator: -	Denominator: -	Exclusion: -	Performance standard: -	Excluded	Conceptualisation and/or Operationalisation	N/A	N/A
589	Woitha, 2012	Documentation of clinical data Clinical record (All settings) The palliative care clinical record contains evidence of documentation of the following items: Physical aspects of care	Numerator: -	Denominator: -	Exclusion: -	Performance standard: -	Excluded	Conceptualisation and/or Operationalisation	N/A	N/A
590	Woitha, 2012	Documentation of clinical data Clinical record (All settings) The palliative care clinical record contains evidence of documentation of the following	Numerator: -	Denominator: -	Exclusion: -	Performance standard: -	Excluded	Conceptualisation and/or Operationalisation	N/A	N/A

		items: Psychological and psychiatric aspects of care								
591	Woitha, 2012	Documentation of clinical data Clinical record (All settings) The palliative care clinical record contains evidence of documentation of the following items: Social aspects of care	Numerator: -	Denominator: -	Exclusion: -	Performance standard: -	Excluded	Conceptualisation and/or Operationalisation	N/A	N/A
592	Woitha, 2012	Documentation of clinical data Clinical record (All settings) The palliative care clinical record contains evidence of documentation of the following items:Spiritual, religious, existential aspects of care	Numerator: -	Denominator: -	Exclusion: -	Performance standard: -	Excluded	Conceptualisation and/or Operationalisation	N/A	N/A
593	Woitha, 2012	Documentation of clinical data Clinical record (All settings) The palliative care clinical record contains evidence of documentation of the following items: Cultural aspects of care	Numerator: -	Denominator: -	Exclusion: -	Performance standard: -	Excluded	Conceptualisation and/or Operationalisation	N/A	N/A
594	Woitha, 2012	Documentation of clinical data Clinical record (All settings) The palliative care clinical record contains evidence of documentation of the following items: Care of imminently dying patient	Numerator: -	Denominator: -	Exclusion: -	Performance standard: -	Excluded	Conceptualisation and/or Operationalisation	N/A	N/A
595	Woitha, 2012	Documentation of clinical data Clinical record (All settings) The palliative care clinical record contains evidence of documentation of the following items: Ethical, legal aspects of care	Numerator: -	Denominator: -	Exclusion: -	Performance standard: -	Excluded	Conceptualisation and/or Operationalisation	N/A	N/A
596	Woitha, 2012	Documentation of clinical data Clinical record (All settings) The palliative care clinical record contains evidence of documentation of the following items: Multidimensional treatment plan	Numerator: -	Denominator: -	Exclusion: -	Performance standard: -	Excluded	Conceptualisation and/or Operationalisation	N/A	N/A
597	Woitha, 2012	Documentation of clinical data Clinical record (All settings) The palliative care clinical record contains evidence of documentation of the following items: Follow up assessment	Numerator: -	Denominator: -	Exclusion: -	Performance standard: -	Excluded	Conceptualisation and/or Operationalisation	N/A	N/A

598	Woitha, 2012	Documentation of clinical data Timley documentation Inpatient setting (Hospital, Palliative care unit, Hospice, Nursing home) Within 24 hours of admission there is documentation of the initial assessment of: Prognosis, Functional status, Pain and other symptoms, Psychosocial symptoms, The patient's capacity to make decisions	Numerator: -	Denominator: -	Exclusion: -	Performance standard: -	Excluded	Conceptualisation and/or Operationalisation	N/A	N/A
599	Woitha, 2012	Documentation of clinical data Timley documentation Inpatient setting (Hospital, Palliative care unit, Hospice, Nursing home) There is documentation that patients reporting pain or other symptoms at the time of admission, had their pain or other symptoms relieved or reduced to a level of their satisfaction within 48 hours of admission	Numerator: -	Denominator: -	Exclusion: -	Performance standard: -	Excluded	Conceptualisation and/or Operationalisation	N/A	N/A
600	Woitha, 2012	Documentation of clinical data Timley documentation Inpatient setting (Hospital, Palliative care unit, Hospice, Nursing home) There is documentation about the discussion of patient preferences within 48 hours of admission	Numerator: -	Denominator: -	Exclusion: -	Performance standard: -	Excluded	Conceptualisation and/or Operationalisation	N/A	N/A
601	Woitha, 2012	Documentation of clinical data Timley documentation Inpatient setting (Hospital, Palliative care unit, Hospice, Nursing home) A discharge/ transfer summary is available in the medical record within 48 hours after discharge/ transfer	Numerator: -	Denominator: -	Exclusion: -	Performance standard: -	Excluded	Conceptualisation and/or Operationalisation	N/A	N/A
602	Woitha, 2012	Documentation of clinical data Timley documentation All settings There is documentation of pain assessment at 4 hour intervals	Numerator: -	Denominator: -	Exclusion: -	Performance standard: -	Excluded	Conceptualisation and/or Operationalisation	N/A	N/A
603	Woitha, 2012	Documentation of clinical data Timley documentation All settings The discussion of patient's preferences is reviewed on a regular basis (in parallel with disease progression) or on request of the patient	Numerator: -	Denominator: -	Exclusion: -	Performance standard: -	Excluded	Conceptualisation and/or Operationalisation	N/A	N/A
604	Woitha, 2012	Documentation of clinical data Timley documentation All settings There is documentation that within 24 hours after patient transfer, the responsible physician in the receiving setting has visited the patient	Numerator: -	Denominator: -	Exclusion: -	Performance standard: -	Excluded	Conceptualisation and/or Operationalisation	N/A	N/A

605	Woitha, 2012	Documentation of clinical data Timley documentation All settings There is documentation that within 24 hours after patient transfer, the new palliative care team in the receiving setting has visited the patient	Numerator: -	Denominator: -	Exclusion: -	Performance standard: -	Excluded	Conceptualisation and/or Operationalisation	N/A	N/A
606	Woitha, 2012	Quality and safety issues Quality policies The palliative care service has a quality improvement program	Numerator: -	Denominator: -	Exclusion: -	Performance standard: -	Excluded	Conceptualisation and/or Operationalisation	N/A	N/A
607	Woitha, 2012	Quality and safety issues Quality policies There is documentation whether targets set for quality improvement have been met	Numerator: -	Denominator: -	Exclusion: -	Performance standard: -	Excluded	Conceptualisation and/or Operationalisation	N/A	N/A
608	Woitha, 2012	Quality and safety issues Quality policies Clinical audit are part of the quality improvement program	Numerator: -	Denominator: -	Exclusion: -	Performance standard: -	Excluded	Conceptualisation and/or Operationalisation	N/A	N/A
609	Woitha, 2012	Quality and safety issues Quality policies The setting uses a program about early initiation of palliative care (e.g. the Gold Standards Framework)	Numerator: -	Denominator: -	Exclusion: -	Performance standard: -	Excluded	Conceptualisation and/or Operationalisation	N/A	N/A
610	Woitha, 2012	Quality and safety issues Adverse events There is a register for adverse events	Numerator: -	Denominator: -	Exclusion: -	Performance standard: -	Excluded	Conceptualisation and/or Operationalisation	N/A	N/A
611	Woitha, 2012	Quality and safety issues Adverse events There is a documented procedure to analyse and follow up adverse events	Numerator: -	Denominator: -	Exclusion: -	Performance standard: -	Excluded	Conceptualisation and/or Operationalisation	N/A	N/A
612	Woitha, 2012	Quality and safety issues Complaint procedure There is a patient complaints procedure	Numerator: -	Denominator: -	Exclusion: -	Performance standard: -	Excluded	Conceptualisation and/or Operationalisation	N/A	N/A
613	Woitha, 2012	Reporting clinical activity of palliative care services The palliative care service uses a database for recording clinical activity	Numerator: -	Denominator: -	Exclusion: -	Performance standard: -	Excluded	Conceptualisation and/or Operationalisation	N/A	N/A
614	Woitha, 2012	Reporting clinical activity of palliative care services The following is part of the database: Diagnosis, Date of diagnosis, Date of referral, Date of admission to the palliative care service, Date of death, Place of death, Preferred place of death	Numerator: -	Denominator: -	Exclusion: -	Performance standard: -	Excluded	Conceptualisation and/or Operationalisation	N/A	N/A

615	Woitha, 2012	Reporting clinical activity of palliative care services From the database the service is able to derive: Time from diagnosis to referral to palliative care, Time from referral to initiation of palliative care, Time from initiation of palliative care to death, Frequency of unplanned consultations with the out-of-hours service for palliative care patients who are at home, Frequency of unplanned hospital admissions of palliative care patients, Percentage of non-oncological patients receiving palliative care	Numerator: -	Denominator: -	Exclusion: -	Performance standard: -	Excluded	Conceptualisation and/or Operationalisation	N/A	N/A
616	Woitha, 2012	Reporting clinical activity of palliative care services Based on the database, an annual report is made about the service	Numerator: -	Denominator: -	Exclusion: -	Performance standard: -	Excluded	Conceptualisation and/or Operationalisation	N/A	N/A
617	Woitha, 2012	Research There is evidence that the palliative care service is involved in research in palliative care (e.g. authorship of publications, research grants)	Numerator: -	Denominator: -	Exclusion: -	Performance standard: -	Excluded	Conceptualisation and/or Operationalisation	N/A	N/A
618	Woitha, 2012	Education All health and social care students have standardised learning objectives for basic training in palliative care	Numerator: -	Denominator: -	Exclusion: -	Performance standard: -	Excluded	Conceptualisation and/or Operationalisation	N/A	N/A
619	Woitha, 2012	Education All health and social care professionals have standardised learning objectives for continuing basic training in palliative care	Numerator: -	Denominator: -	Exclusion: -	Performance standard: -	Excluded	Conceptualisation and/or Operationalisation	N/A	N/A
620	Woitha, 2012	Education There is a program for specialised training in palliative care for professionals working in a service that provides specialised palliative care	Numerator: -	Denominator: -	Exclusion: -	Performance standard: -	Excluded	Conceptualisation and/or Operationalisation	N/A	N/A
621	ASCO QOPI, 2018	Pain assessed on either of the last two visits before death	Numerator: -	Denominator: -	Exclusion: -	Performance standard: -	Included	N/A	07-DOMAIN 7 OPTIMAL TREATMENT OF SYMPTOMS AND PROVIDING COMFORT	Process
622	ASCO QOPI, 2018	Pain intensity quantified on either of the last two visits before death	Numerator: -	Denominator: -	Exclusion: -	Performance standard: -	Included	N/A	07-DOMAIN 7 OPTIMAL TREATMENT OF SYMPTOMS AND PROVIDING COMFORT	Process

623	ASCO QOPI, 2018	Plan of care for moderate severe pain documented on either of the last two visits before death	Numerator: -	Denominator: -	Exclusion: -	Performance standard: -	Included	N/A	07-DOMAIN 7 OPTIMAL TREATMENT OF SYMPTOMS AND PROVIDING COMFORT	Process
624	ASCO QOPI, 2018	Pain addressed appropriately (defect- free on ASCO QOPI QI 295, 296, 297))	Numerator: -	Denominator: -	Exclusion: -	Performance standard: -	Included	N/A	07-DOMAIN 7 OPTIMAL TREATMENT OF SYMPTOMS AND PROVIDING COMFORT	Outcome
625	ASCO QOPI, 2018	Hospice enrollment	Numerator: -	Denominator: -	Exclusion: -	Performance standard: -	Excluded	Relevance to long-term care settings	N/A	N/A
626	ASCO QOPI, 2018	Hospice enrollment or palliative care referral services	Numerator: -	Denominator: -	Exclusion: -	Performance standard: -	Excluded	Relevance to long-term care settings	N/A	N/A
627	ASCO QOPI, 2018	Hospice enrollment within three days of death (Lower score -Better)	Numerator: -	Denominator: -	Exclusion: -	Performance standard: -	Excluded	Relevance to long-term care settings	N/A	N/A
628	ASCO QOPI, 2018	Hospice enrollment and enrolled more more than three days before death	Numerator: -	Denominator: -	Exclusion: -	Performance standard: -	Excluded	Relevance to long-term care settings	N/A	N/A
629	ASCO QOPI, 2018	Hospice enrollement within 7 days before death	Numerator: -	Denominator: -	Exclusion: -	Performance standard: -	Excluded	Relevance to long-term care settings	N/A	N/A
630	ASCO QOPI, 2018	Hospice enrollment and enrolled more more than seven days before death	Numerator: -	Denominator: -	Exclusion: -	Performance standard: -	Excluded	Relevance to long-term care settings	N/A	N/A
631	ASCO QOPI, 2018	For patients not referred, hospice or pallitive care discussed within the last two months of life	Numerator: -	Denominator: -	Exclusion: -	Performance standard: -	Included	N/A	05-DOMAIN 5 PROGNOSTICATIO N AND TIMELY RECOGNITION OF DYING	Process
632	ASCO QOPI, 2018	Hospice enrollement, palliative care referral/services, or documented discussion	Numerator: -	Denominator: -	Exclusion: -	Performance standard: -	Excluded	Relevance to long-term care settings	N/A	N/A
633	ASCO QOPI, 2018	Chemotherapy administered within the last two weeks of life (Lower score better)	Numerator: -	Denominator: -	Exclusion: -	Performance standard: -	Excluded	Procedural Relevance	N/A	N/A
634	ASCO QOPI, 2018	Percentage of patients who died from cancer with more than one emergency visit in the last 30 days of life (Lower score - Better)	Numerator: -	Denominator: -	Exclusion: -	Performance standard: -	Excluded	Procedural Relevance	N/A	N/A
635	ASCO QOPI, 2018	Percentage of patients who died from cancer admitted to the ICU in the	Numerator: -	Denominator: -	Exclusion: -	Performance standard: -	Excluded	Procedural Relevance	N/A	N/A

		last 30<br>dasy of life (Lower score - Better)								

Appendix 3 - References – 2018 update

Earle, 2006

- National Quality Forum. National voluntary consensus standards for symptom management and end-of-life care in cancer patients. Washington DC: National Quality Forum; 2006 Available from: http://www.qualityforum.org
- Earle CC, Park ER, Lai B, Weeks JC, Ayanian JZ, Block S. Identifying potential indicators of the quality of end-of-life cancer care from administrative data. *J Clin Oncol*. 2003 Mar 15;21(6):1133-8
- Earle CC, Neville BA, Landrum MB, Souza JM, Weeks JC, Block SD, Grunfeld E, Ayanian JZ. Evaluating claims-based indicators of the intensity of end-of-life cancer care. *Int J Qual Health Care*. 2005 Dec;17(6):505-9

We only used the quality indicators which are accepted by the National Quality Forum, 2006.

Subject quality indicator set: Palliative cancer care

Grunfeld, 2008

- Grunfeld E, Urquhart R, Mykhalovskiy E, Folkes A, Johnston G, Burge FI, Earle CC, Dent S. Toward population-based indicators of quality end-of-life care: testing stakeholder agreement. *Cancer.* 2008 May 15;112(10):2301-8.
- Grunfeld E, Lethbridge L, Dewar R, Lawson B, Paszat LF, Johnston G, Burge F, McIntyre P, Earle CC. Towards using administrative databases to measure population-based indicators of quality of end-of-life care: testing the methodology. *Palliat Med*. 2006 Dec;20(8):769-77

Subject quality indicator set: Palliative cancer care

One of the references by Grunfeld et al. (2006) found by Pasman et al. seems to regard a new set of indicators, and is in this update considered as describing a new set of indicators, together with a newly retrieved reference.

Yabroff, 2004

- Yabroff KR, Mandelblatt JS, Ingham J. The quality of medical care at the end-of-life in the USA: existing barriers and examples of process and outcome measures. *Palliat Med*. 2004 Apr;18(3):202-16

Subject quality indicator set: Palliative cancer care
Not all proposed quality indicators in the publication met the inclusion criteria for a quality indicator.

Lorenz, 2009

- Lorenz KA, Dy SM, Naeim A, Walling AM, Sanati H, Smith P, Shanman R, Roth CP, Asch SM. Quality measures for supportive cancer care: the Cancer Quality-ASSIST Project. *J Pain Symptom Manage.* 2009 Jun;37(6):943-64.
- Dy SM, Lorenz KA, O'Neill SM, Asch SM, Walling AM, Tisnado D, et al. Cancer Quality-ASSIST supportive oncology quality indicator set: feasibility, reliability, and validity testing. Cancer 2010 Jul 1;116(13):3267-75.

Subject quality indicator set: Palliative cancer care

Miyashita, 2008

- Miyashita M, Nakamura A, Morita T, Bito S. Identification of quality indicators of end-of-life cancer care from medical chart review using a modified Delphi method in Japan. *Am J Hosp Palliat Care.* 2008 Feb-Mar;25(1):33-8.
- Sato K, Miyashita M, Morita T, Sanjo M, Shima Y, Uchitomi Y. Reliability assessment and findings of a newly developed quality measurement instrument: quality indicators of end-of-life cancer care from medical chart review at a Japanese regional cancer center. *J Palliat Med.* 2008 Jun;11(5):729-37.

Subject quality indicator set: Palliative cancer care

Peruselli, 1997

- Peruselli C, Marinari M, Brivio B, Castagnini G, Cavana M, Centrone G, Magni C, Merlini M, Scaccabaro GL, Paci E. Evaluating a home palliative care service: development of indicators for a continuous quality improvement program. *J Palliat Care*. 1997 Autumn;13(3):34-42.

Subject quality indicator set: Home palliative care

Not all proposed quality indicators in the publication met the inclusion criteria for a quality indicator.

Keay, 1994

- Keay TJ, Fredman L, Taler GA, Datta S, Leverson SA. Indicators of quality medical care for the terminally ill in nursing homes. *J Am Geriatr Soc*. 1994 Aug;42(8):853-60

Subject quality indicator set: Palliative nursing home care

Not all proposed quality indicators in the publication met the inclusion criteria for a quality indicator.

Lorenz, 2007; Saliba, 2004

- Wenger NS, Rosenfeld K. Quality indicators for end-of-life care in vulnerable elders. *Ann Intern Med.* 2001 Oct 16;135(8 Pt 2):677-852
- Saliba D, Solomon D, Rubenstein L, Young R, Schnelle J, Roth C, Wenger N. Feasibility of quality indicators for the management of geriatric syndromes in nursing home residents. *J Am Med Dir Assoc*. 2004 Sep-Oct;5(5):310-9
- Lorenz KA, Rosenfeld K, Wenger N. Quality indicators for palliative and end-of-life care in vulnerable elders. *J Am Geriatr Soc*. 2007 Oct;55 Suppl 2:S318-26.
- Anonymous. Assessing care of vulnerable elders-3 quality indicators*. J Am Geriatr Soc* 2007 Oct;55 Suppl 2:S464-S487.

Subject quality indicator set: Vulnerable elderly in end-of-life care

We used the quality indicators described in the updated (2007) version of the ACOVE set. Saliba et al. used the ACOVE set of 2001 to test feasibility of the quality indicators for nursing home patients. Two indicators were not used in the 2007 version, and are separately included in this review.

van der Ploeg, 2008

- van der Ploeg E, Depla MF, Shekelle P, Rigter H, Mackenbach JP. Developing quality indicators for general practice care for vulnerable elders; transfer from US to The Netherlands. Qual Saf Health Care. 2008 Aug;17(4):291-5.

Subject quality indicator set: Vulnerable elderly in end-of-life care

Shortened and adapted version of the ACOVE end-of-life care subset. Overlapping quality indicators are only mentioned once.

National Hospice and Palliative Care Organization (NHPCO), 2006

- National Quality Forum. National voluntary consensus standards for symptom management and end-of-life care in cancer patients. Washington DC: National Quality Forum; 2006 Available from: http://www.qualityforum.org

Subject quality indicator set: Family evaluation of hospice care

We only selected the quality indicators which are accepted by the National Quality Forum.

****UPDATE**
After 2015, the Family Evaluation of Hospice Care survey (FEHC) submitted to the National Hospice and Palliative Care Organization (NHPCO), was replaced by the Consumer Assessment of Healthcare Providers and Systems Hospice Survey aka CAHPS® Hospice survey, developed by the Centers for Medicare and Medicaid services (CMS). Survey results are used to calculate eight (8) patient experience measures

NEW Centers for Medicare and Medicaid services, 2015
Centers for Medicare and Medicaid services: Hospice Quality Reporting Program – Current Measures 2018 Available from https://www.cms.gov/Medicare/Quality-Initiatives-Patient-Assessment-Instruments/Hospice-Quality-Reporting/Current-Measures.html (accessed September 7, 2018)

Price et al. Hospice experience of care survey Development and Field Test 2014. Available from http://www.hospicecahpssurvey.org/en/ (accessed September 7, 2018)

Schenck, 2010

- Schenck AP, Rokoske FS, Durham DD, Cagle JG, Hanson LC. The PEACE Project: identification of quality measures for hospice and palliative care. *J Palliat Med.* 2010 Dec;13(12):1451-9.

Subject quality indicator set: Hospice or palliative care

****UPDATE**
Of the 34 PEACE quality measures developed in 2010, eight (8) are used in the Centers for Medicare and Medicaid services (CMS) Hospice Quality Reporting Program and endorsed by the National Quality Forum (NQF)

Schenck AP, Rokoske FS, Durham DD, Cagle JG, Hanson LC. Quality Measures for Hospice and Palliative care: Piloting the PEACE measures *J Palliat Med.* 2014 Jul;17(7):769-75

Centers for Medicare and Medicaid services: Hospice Quality Reporting Program – Current Measures 2018 Available from https://www.cms.gov/Medicare/Quality-Initiatives-Patient-Assessment-Instruments/Hospice-Quality-Reporting/Current-Measures.html (accessed September 7, 2018)

Mularski, 2006; Nelson, 2006

- Mularski RA, Curtis JR, Billings JA, Burt R, Byock I, Fuhrman C, Mosenthal AC, Medina J, Ray DE, Rubenfeld GD, Schneiderman LJ, Treece PD, Truog RD, Levy MM. Proposed

quality measures for palliative care in the critically ill: a consensus from the Robert Wood Johnson Foundation Critical Care Workgroup. *Crit Care Med*. 2006 Nov;34(11 Suppl):S404-11
- Nelson JE, Mulkerin CM, Adams LL, Pronovost PJ. Improving comfort and communication in the ICU: a practical new tool for palliative care performance measurement and feedback. *Qual Saf Health Care*. 2006 Aug;15(4):264-71
- Clarke EB, Curtis JR, Luce JM, Levy M, Danis M, Nelson J, Solomon MZ; Robert Wood Johnson Foundation Critical Care End-Of-Life Peer Workgroup Members. Quality indicators for end-of-life care in the intensive care unit. *Crit Care Med*. 2003 Sep;31(9):2255-62

Subject quality indicator set: ICU end-of-life care

Mularski and Nelson used the same preliminary set of quality indicators for the ICU described by Clarke et al, 2003, but selected different quality indicators. We selected the quality indicators which are described in both publication and the quality indicators which are only described by Mularski or Nelson.

Twaddle, 2007
- Twaddle ML, Maxwell TL, Cassel JB, Liao S, Coyne PJ, Usher BM, Amin A, Cuny J. Palliative care benchmarks from academic medical centers. *J Palliat Med*. 2007 Feb;10(1):86-98

Subject quality indicator set: Hospital-based palliative care

Claessen, 2011
- Claessen SJ, Francke AL, Belarbi HE, Pasman HR, van der Putten MJ, Deliens L. A new set of quality indicators for palliative care: process and results of the development trajectory. *J Pain Symptom Manage.* 2011 Aug;42(2):169-82.
- Brandt HE, Francke AL, Pasman HRW, et al. Indicatoren voor palliatieve zorg: ontwikkeling en toetsing van een set kwaliteitsindicatoren voor de palliatieve zorgverlening. (Quality indicators for palliative care: development and testing of a set of quality indicators for palliative care [in Dutch]). Utrecht: NIVEL, 2009. Available from http://www.nivel.nl/sites/default/files/bestanden/Rapport-indicatoren-palliatieve-zorg.pdf.

Subject quality indicator set: Palliative care for adult patients in all settings where palliative care is provided

Not all proposed quality indicators in the publication met the inclusion criteria for a quality indicator.

Eagar, 2010
- Eagar K, Watters P, Currow DC, Aoun SM, Yates P. The Australian Palliative Care Outcomes Collaboration (PCOC)--measuring the quality and outcomes of palliative care on a routine basis. *Aust Health Rev.* 2010 May;34(2):186-92.
- Palliative Care Outcomes Collaboration (PCOC): National Benchmarks and Targets. 2009. Available from: http://ahsri.uow.edu.au/pcoc/benchmarkmeasures/index.html.

Subject quality indicator set: Specialist palliative care settings

Not all proposed quality indicators in the publication met the inclusion criteria for a quality indicator.

****UPDATE**
After feedback from services attending the 2012 PCOC benchmarking workshops, the benchmark for Outcome Measure 2 (time in the unstable phase) was revised, with three benchmarks amalgamated into one. Following on from the implementation of the Version 3 dataset, in 2014, Benchmark 1 was changed from 'Time from referral to first contact' to 'Time from date ready for care to episode start'. In 2015, PCOC introduced three new patient outcome measures and associated benchmarks. These three outcome measures relate to distress due to fatigue and breathing problems (both rated by the SAS) and family / carer problems (as rated by the PCPSS).

NEW Palliative Care Outcomes Collaboration, 2015
Palliative Care Outcomes Collaboration (PCOC): National Outcome Measures and Benchmarks 2015. Available from: https://ahsri.uow.edu.au/pcoc/4clinicians/outcomes-and-benchmarks/index.html (February 25, 2019)

Quality Markers, 2009

- Department of Health. End of Life Care Strategy. Quality Markers and measures for end of life care, Available from: http://www.dh.gov.uk/health/2011/07/end-of-life-care-strategy/

Subject quality indicator set: Several settings where palliative care is provided

Not all proposed quality indicators in the publication met the inclusion criteria for a quality indicator.

****UPDATE**
National Institute for Health and Care Excellence (NICE) End-of-life care for Adults – Quality Standard 2011 Available from https://www.nice.org.uk/guidance/qs13/resources/end-of-life-care-for-adults-pdf-2098483631557 (accessed September 7, 2018)

October 2013: Following the independent review of the use of the Liverpool Care Pathway (LCP) for the dying patient and the subsequent announcement of the phasing out of the LCP, interim amendments have been made to this quality standard. Direct and indirect references to the LCP have been removed to reflect the gradual phasing out of the LCP and ensure that this quality standard remains current.

March 2017: Statement 11 on care in the last days of life has been removed. This is because NICE has published a quality standard for care of dying adults in the last days of life, which covers this topic in detail and replaces this statement.

NEW National Institute for Health and Care Excellence, 2017
National Institute for Health and Care Excellence (NICE) Care of Dying Adults in the last days of life – Quality Standard 2017 Available from https://www.nice.org.uk/guidance/qs144 (accessed September 7, 2018)

ELCQuA, 2011

- End of Life Care Quality Assessment (ELCQuA), 2011. Available from: http://www.elcqua.nhs.uk

Subject quality indicator set: Several settings where palliative care is provided

Not all proposed quality indicators in the publication met the inclusion criteria for a quality indicator.

****UPDATE**
Following a 2015 review by the National End-of-life care Intelligence Network (NEoLCIN), the ELCQuA (End of Life Care Quality Assessment tool) was withdrawn from 31 March 2016.

****

NEW Hui, 2015
Hui, D., et al. (2015). "Indicators of integration of oncology and palliative care programs: an international consensus." Annals of Oncology **26**(9): 1953-1959.

NEW Leemans, 2017
Leemans, K., et al. (2017). "Systematic quality monitoring for specialized palliative care services: development of a minimal set of quality indicators for palliative care study (QPAC)." American Journal of Hospice and Palliative Medicine® **34**(6): 532-546.

NEW Neuss, 2005
Neuss, M. N., et al. (2005). "A process for measuring the quality of cancer care: The Quality Oncology Practice Initiative." Journal of Clinical Oncology **23**(25): 6233-6239.

American Society of Clinical Oncology - Quality Oncology Practice Initiative (ASCO – QOPI) https://practice.asco.org/sites/default/files/drupalfiles/QOPI-2018-Reporting-Tracks-Public-Posting.pdf (accessed September 7, 2018)

We only selected the quality indicators which are accepted by the National Quality Forum

NEW Odenheimer, 2013
Odenheimer, G., et al. (2013). "Quality improvement in neurology Dementia management quality measures." Neurology **81**(17): 1545-1549.

NEW Raijjmakers, 2012
Raijmakers, N., et al. (2012). "Quality indicators for care of cancer patients in their last days of life: literature update and experts' evaluation." Journal of Palliative Medicine **15**(3): 308-316.

NEW Martin-Khan, 2013
Martin-Khan, M. (2013) "Methodology for developing quality indicators for the care of older people in the Emergency Department" BMC Emergency Medicine 13(23)

NEW Schnitker, 2015
Schnitker, L. M., et al. (2015). "Structural quality indicators to support quality of care for older people with cognitive impairment in emergency departments." Academic Emergency Medicine **22**(3): 273-284.

NEW Schnitker, L. M., et al. (2015). "Process quality indicators targeting cognitive impairment to support quality of care for older people with cognitive impairment in emergency departments." Academic Emergency Medicine **22**(3): 285-298.

NEW Sinuff, 2015
Sinuff, T., et al. (2015). "Improving end-of-life communication and decision making: the development of a conceptual framework and quality indicators." Journal of Pain and Symptom Management **49**(6): 1070-1080.

NEW van Riet Paap, 2014
van Riet Paap, J., et al. (2014). "Consensus on quality indicators to assess the organisation of palliative cancer and dementia care applicable across national healthcare systems and selected by international experts." BMC Health Services Research **14**(1): 396.

NEW Walling, 2017
Walling, A., et al. (2017). "Palliative care quality indicators for patients with end-stage liver disease due to cirrhosis." Digestive Diseases and Sciences **62**(1): 84-92.

NEW Woitha, 2012
Woitha, K., et al. (2012). "Development of a set of process and structure indicators for palliative care: the Europall project." BMC Health Services Research **12**(1): 381.
